# Identification of a Notch transcriptomic signature for breast cancer

**DOI:** 10.1186/s13058-023-01757-7

**Published:** 2024-01-03

**Authors:** Eike-Benjamin Braune, Felix Geist, Xiaojia Tang, Krishna Kalari, Judy Boughey, Liewei Wang, Roberto A. Leon-Ferre, Antonino B. D’Assoro, James N. Ingle, Matthew P. Goetz, Julian Kreis, Kang Wang, Theodoros Foukakis, Anita Seshire, Dirk Wienke, Urban Lendahl

**Affiliations:** 1 Department of Cell and Molecular Biology, Karolinska Institutet, Stockholm, Sweden; 2 Merck Healthcare KGaA, Darmstadt, Germany; 3 Department of Quantitative Health Sciences, Mayo Clinic, Rochester, MN USA; 4 Department of Surgery, Mayo Clinic, Rochester, MN USA; 5 Department of Molecular Pharmacology and Experimental Therapeutics, Mayo Clinic, Rochester, MN USA; 6 Department of Oncology, Mayo Clinic, Rochester, MN USA; 7 Department of Oncology and Pathology, Karolinska Institutet, Stockholm, Sweden; 8 Merck KGaA, Darmstadt, Germany

**Keywords:** Breast cancer, Basal-like, Breast cancer stem cell, Notch signalling, Transcriptomic signature, Therapy, Therapy resistance, Triple-negative, Diagnostics

## Abstract

**Background:**

Dysregulated Notch signalling contributes to breast cancer development and progression, but validated tools to measure the level of Notch signalling in breast cancer subtypes and in response to systemic therapy are largely lacking. A transcriptomic signature of Notch signalling would be warranted, for example to monitor the effects of future Notch-targeting therapies and to learn whether altered Notch signalling is an off-target effect of current breast cancer therapies. In this report, we have established such a classifier.

**Methods:**

To generate the signature, we first identified Notch-regulated genes from six basal-like breast cancer cell lines subjected to elevated or reduced Notch signalling by culturing on immobilized Notch ligand Jagged1 or blockade of Notch by *γ*-secretase inhibitors, respectively. From this cadre of Notch-regulated genes, we developed candidate transcriptomic signatures that were trained on a breast cancer patient dataset (the TCGA-BRCA cohort) and a broader breast cancer cell line cohort and sought to validate in independent datasets.

**Results:**

An optimal 20-gene transcriptomic signature was selected. We validated the signature on two independent patient datasets (METABRIC and Oslo2), and it showed an improved coherence score and tumour specificity compared with previously published signatures. Furthermore, the signature score was particularly high for basal-like breast cancer, indicating an enhanced level of Notch signalling in this subtype. The signature score was increased after neoadjuvant treatment in the PROMIX and BEAUTY patient cohorts, and a lower signature score generally correlated with better clinical outcome.

**Conclusions:**

The 20-gene transcriptional signature will be a valuable tool to evaluate the response of future Notch-targeting therapies for breast cancer, to learn about potential effects on Notch signalling from conventional breast cancer therapies and to better stratify patients for therapy considerations.

**Supplementary Information:**

The online version contains supplementary material available at 10.1186/s13058-023-01757-7.

## Background

Breast cancer is the leading cause of cancer death in women in the world [1, 2]. Breast cancer classification is based on immunohistochemical expression of specific receptors (the oestrogen (ER), progesterone (PR) and HER2 receptors). Using a molecular classification scheme, subtypes may be classified based on gene expression profiles such as the PAM50. With this molecular classifier, five major subtypes are recognized: luminal A, luminal B, HER2-enriched, normal-like and basal-like [3]. A sixth subtype, claudin-low, was added later, although this subtype remains somewhat controversial [4]. The basal-like and claudin-low tumours best represent triple negative breast cancer (TNBC), which lacks expression of ER, PR and without amplification of HER2 (for review, see [5]).

Endocrine therapies either alone or with targeted therapies such as CDK 4/6 inhibitors are standard of care for ER^+^ breast cancer. Patients with HER2^+^ tumours are treated with antibodies/antibody drug conjugates and tyrosine kinase inhibitors directed against HER2. Additional therapeutic options are PARP inhibitors in BRCA-mutant breast cancers. There are limited options for tailored therapies for TNBC, which is the most aggressive subtype and is, therefore, treated either by chemotherapy with or without immune checkpoint inhibitors, and recently, with novel antibody–drug conjugates, e.g. sacituzumab govitecan or trastuzumab deruxtecan for HER2-low tumours. The limited options for specific therapies for TNBC combined with the development of resistance to therapies for ER+, PR+ and HER2 + breast cancer highlight that therapeutic challenges still are considerable [1], and that a deeper understanding of the molecular mechanisms driving development and progression of breast cancer is warranted.

**Fig. 1:**
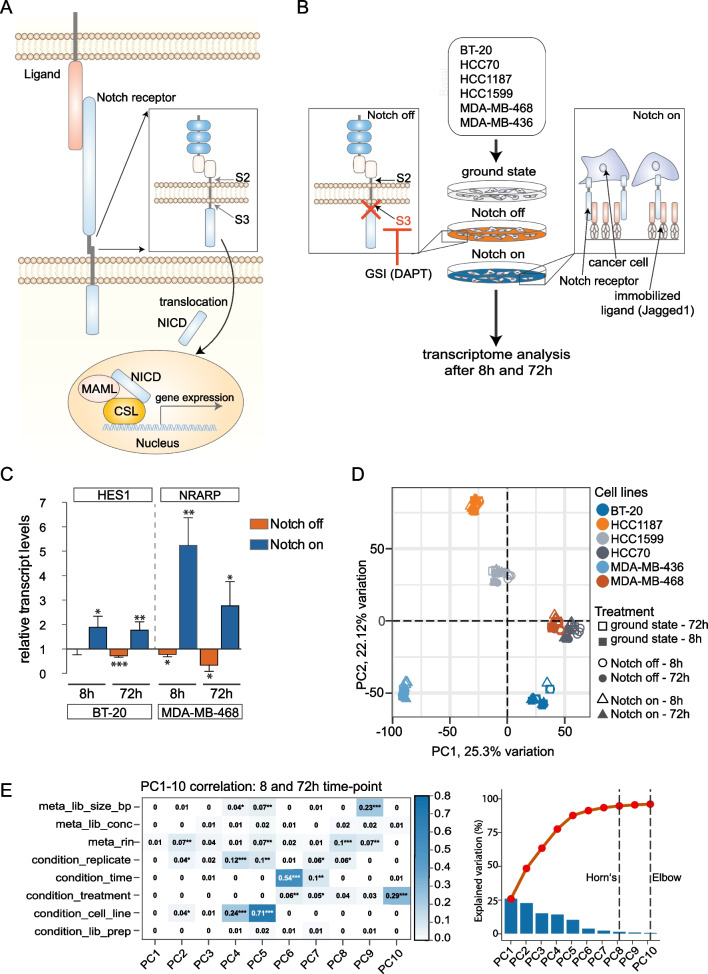
An assay to identify Notch-regulated genes in basal-like breast cancer cell lines. **A** A schematic representation of the Notch signalling pathway. Binding of the Notch receptor to a ligand presented on the neighbouring cell leads to S2 proteolytic processing (S2), which is followed by S3 processing in the membrane (S3) by the *γ*-secretase complex. S3 processing results in the liberation of the Notch intracellular domain (NICD) and its translocation into the nucleus. In the nucleus, NICD together with CSL and MAML forms a ternary transcriptional complex that regulates expression of Notch downstream genes. **B** Schematic depiction of the assay to identify Notch-regulated genes to establish a Notch transcriptomic signature. Six basal-like breast cancer cell lines (as indicated) were seeded in three replicates. Notch activation (“Notch on”) was achieved by cultivating the cell lines on immobilized ligands, and inhibition of Notch signalling (“Notch off”) was achieved by adding the y-secretase inhibitor (GSI) DAPT, which blocks S3-cleavage. Cultivation of the cells after addition of DMSO was carried out as control (“ground state”). Bulk transcriptomes were captured after 8 and 72 h of culturing under the different conditions. **C** Expression of known Notch target genes (HES1, NRARP) was evaluated after 8 or 72 h in the BT-20 and MDA-MB-468 cell lines, as indicated (*p < 0.05, **p < 0.01 and ***p < 0.001). **D** Principal component analysis of the transcriptomic data from the six cell lines under “Notch on”, “Notch off” or “ground state” conditions at 8 or 72 h, as indicated. A total of 12,459 genes were used for the PCA. **E** Correlation of the principal components to different experimental parameters in the transcriptomic dataset from the six basal-like cell lines. The Horn’s and Elbow represent metrices to identify the optimal number of principal components [10, 11]

In addition to frequent mutations in a smaller set of genes, including BRCA and p53, it is increasingly recognized that dysregulation of the Notch signalling pathway plays a role in breast cancer [6]. Notch signalling is an evolutionarily highly conserved cell–cell communication mechanism, which regulates cell fate decisions and tissue homeostasis in most organs, including the mammary gland [7]. Ligand–receptor interaction leads to proteolytic processing events culminating in the liberation of the Notch receptor intracellular domain (Notch ICD) through cleavage (site 3 (S3) cleavage) conducted by the *γ*-secretase complex (see Fig. 1A for a schematic depiction of Notch receptor signalling). Upon translocation to the cell nucleus, Notch ICD forms a trimeric complex with MAML and the DNA-binding protein CSL (a.k.a. RBPJ or CBF-1) to regulate downstream gene activation [8, 9] (Fig. 1A).

Notch signalling is important for normal mammary development and promotes commitment to the luminal lineage [12], which generates ductal and alveolar cells [13]. Loss of Notch signalling in the mouse compromises myoepithelial differentiation, which normally generates cells forming the basal layer, while promoting luminal cell differentiation [14]. Several lines of evidence support a role for hyperactivated Notch signalling contributing to breast cancer initiation and progression. Approximately 5–10% of TNBC tumours harbour mutations in the negative regulatory region or PEST domain of the NOTCH1 and 2 receptor genes, resulting in gain-of-function versions of the receptors [15, 16]. Moreover, elevated expression of JAGGED1, NOTCH1, NOTCH3 or NOTCH4 is observed in TNBC and associated with poor clinical prognosis [17–22]; for review see [23]. NOTCH3 gene amplifications are also found in TNBC [24]. It has been proposed that the level of Notch signalling is higher in basal-like breast cancer as compared to, for example ER^+^ and HER2^+^ breast cancer [6]. Notch signalling may, however, be elevated in ER^+^ and HER2^+^ breast cancer following endocrine or HER2-blocking therapies [25–29]. Mechanistically, Notch is an inducer of MYC (for review see [30]) and NOTCH1 and MYC expression positively correlates in breast cancer biopsies [31]. Furthermore, in a screen for genes promoting mammary tumour development in BRCA1-deficient mice, Notch1 was identified as a strong driver [32], and there are several reports indicating that elevated Notch signalling induces and accelerates mammary tumour formation [33–38]. A role for Notch signalling in tumour–stroma interaction was recently revealed in breast ductal cell carcinoma in situ (DCIS) [39], and blocking of Notch signalling led to reduced growth of DCIS tumour spheres [40].

Collectively, these observations support the notion that hyperactivated Notch signalling contributes to breast cancer and that the level of Notch signalling may be altered in response to first-line therapies [6]. Development of tools by which the level of Notch signalling can be determined is, however, still largely work in progress, and to have access to a robust Notch transcriptomic signature that can read out the level of Notch signalling in breast cancer would be important, both to obtain better insights into which breast cancer forms show hyperactivated Notch signalling and to understand how the level of Notch signalling is subsequently affected by existing therapies. Notch-targeting therapy development represents an active area of research and although no Notch-targeting therapies are yet in clinical use, there are preclinical progress and ongoing clinical trials for some therapy candidates, for review see [41, 42]. y-secretase inhibitors (GSI) that prevent the receptor from being cleaved at the S3 site (Fig. 1A) have been intensively researched, and a recently developed GSI, nirogacestat, has shown promising effects in desmoid tumours, and a randomized phase III clinical is currently being conducted (see review [41]). The small molecule inhibitor CB-103, which targets the Notch transcriptional complex [43], proved to be well-tolerated in a phase I study of adenoid cystic carcinoma. While showing limited antitumour activity as monotherapy [44], a complete clinical response in a patient with relapsed and refractory T-ALL was achieved when used in combination with ongoing therapy [45]. Notch receptor- or ligand-specific antibodies represent another potential therapy avenue, which have the advantage of blocking the functions of individual receptors or ligands, thus avoiding potential safety issues associated with pan-Notch inhibition[41, 42].

In the light of this progress towards Notch-targeting therapies, there is an obvious need for more refined readouts to monitor the effects of therapy candidates on Notch signalling, as well as to enable better patient stratification for Notch-targeting therapies. Improved Notch readouts are also warranted to understand whether currently used therapies in various ways affect the level of Notch signalling in the patient, which may have unwanted consequences. In this report, we establish a 20-gene Notch transcriptomic signature by analysing Notch-related transcriptomic changes in a panel of basal-like breast tumour cell lines, followed by tuning in a breast cancer patient dataset. We also report the use of the signature in patient datasets from different breast cancer subtypes and following first-line treatment.

## Methods

### Cell culture

The breast cancer cell lines (BT-20, HCC70, HCC1187, HCC1599, MDA-MB-468 and MDA-MB-436) used for establishing the Notch transcriptomic signature were purchased fresh from the American Type Culture Collection (ATCC) and maintained in RPMI 1640 medium (ATCC’s modification, cat #A1049101, Thermo Fisher Scientific) with 10% fetal bovine serum (FBS, heat inactivated, cat #A3840402, Thermo Fisher Scientific) and 1% penicillin–streptomycin (cat #15140-122, Thermo Fisher Scientific). The 19 breast cancer cell lines used to select the final signature were maintained as described [46]. Accutase (cat # A6964-100ML, Sigma-Aldrich) was used to detach the cells prior to seeding on coated plates and during maintenance. All cell lines were cultivated with 5% CO2 at 37 °C.

### Activation and blockage of Notch signalling

Activation of Notch signalling in the cell lines used to select the signature was achieved as previously described [47] with minor modifications: 1µg rat Jagged1-Fc (cat #599-JG, R and D systems) or ChromPure Human IgG, Fc (cat #009-000-008, Jackson ImmunoResearch) was used instead of 2 µg. Activation of Notch signalling in the cell lines used to establish a Notch transcriptomic signature was performed as described above with minor modifications: Tissue culture-treated plates were incubated over night at 4 °C or for 1 h at room temperature with 5-µg/ml Protein G (cat #21193, Thermo Fisher Scientific) in PBS. Non-specific binding was blocked by incubating coated plates with 1 × blocking buffer (Thermo Fisher Scientific, cat #37525), and plates were incubated with immobilized ligand or Fc control for 1.5 h at room temperature. Blockage of Notch signalling was in both cases achieved by adding 10-µM DAPT (InSolution™ *γ*-Secretase Inhibitor IX, cat #565784, MerckMillipore) directly to the cell culture medium, and cells were incubated with DAPT for 8 h and 72 h. Non-GSI-treated cells (Fc control, Jagged1-Fc) were treated with equimolar volumes of carrier control (DMSO, cat #D8418-50ML, Sigma-Aldrich).

### RNA preparation

Total RNA from cell lines used to establish the Notch transcriptomic signature was isolated using the RNeasy Mini kit (cat #74104, Qiagen) according to the manufacturer’s instructions. RNA concentration was determined using Qubit RNA High sensitivity kit (cat #Q32852, Thermo Fisher Scientific), and RNA integrity score was determined using Bioanalyzer RNA 6000 Nano Kit (cat # 5067–1511, Agilent Technologies) for at least one sample per group. All samples were in addition controlled for quality at the NGI facility at the SciLifeLab laboratory (Stockholm, Sweden), and samples that failed the internal reception control were excluded from library preparation.

### qRT-PCR

qRT-PCR was performed as described elsewhere [76] with some minor modifications: RNA prepared from the cell lines to select the final signature was reverse-transcribed using Maxima First-Strand cDNA synthesis kit (cat #EP0741, Thermo Scientific), and RNA extracted from cell lines used for determining the Notch signature was prepared using iScript Reverse Transcription Supermix (cat # 1708841, Bio-rad). cDNA was then diluted 1:4 in nuclease-free H_2_0. qRT-PCR was performed either on Applied Biosystems 7500 Real-Time PCR System (ABI 7500; Applied Biosystems) or on a C1000 Touch thermal cycler (Bio-rad), and gene expression was detected with Power SYBR Green (cat # 4367659, Thermo Fisher Scientific) or SsoAdvanced Universal SYBR Green Supermix (cat # 1725274, Bio-rad). Gene expression was determined by normalizing first to GAPDH mRNA expression, and fold expression change was calculated using the ΔΔCT method (ΔΔCT = ΔCt sample-Δct control). Primers used in qRT-PCR are listed in Additional file 2: Table S1.

### Statistical analysis for qRT-PCR, BEAUTY and PROMIX data

Statistical analysis of relative transcript levels of Notch target genes in the six basal-like breast cancer cell lines was performed by analysing column means of DMSO control to Notch “off” (DAPT) or Notch “on” (Jagged1) condition using parametrical, unpaired t-test assuming standard distribution of data. At least three biological samples were analysed per condition (DAPT treatment of HCC70 cells at the 8-h timepoint did not generate CT values for Notch target genes). Mean GSVA scores of breast cancer subtype cohorts of the BEAUTY dataset were compared using one-way ANOVA Tukey's multiple comparison test. TNBC responder and non-responder groups were compared using unpaired, parametrical t-test. Standard distribution of data was assessed using Shapiro–Wilk and D'Agostino and Pearson omnibus normality test, and all datasets, except the HER2 + responder cohort, passed the normality test (alpha = 0.05). The sample size for the LumB responder group was too small to test for data distribution (*n* = 2). Graphs and statistical analysis were generated and carried out using Graphpad’s Prism (V10). Analysis of data from the PROMIX dataset was conducted as described [48].

### Software

Figures were generated using Adobe Illustrator V6 and V28.0. Plots were polished to enhance visual experience and readability of data (incl. change of colour, outlines and text). No data were changed, and original plots can be extracted from the provided code.

### RNA-library preparation and sequencing

RNA-library construction and sequencing were performed at the National Genomics Infrastructure (NGI) facility at the SciLifelab (Stockholm, Sweden). RNA libraries of samples used for Notch signature gene determination were constructed using Illumina TruSeq Stranded mRNA protocol (poly-A-selection). Clustering was done by “cBot”, and samples were sequenced on NovaSeq6000 (NovaSeq Control Software 1.7.5/RTA v3.4.4) with a 151nt(Read1)-10nt(Index1)-10nt(Index2)-151nt(Read2) setup using “NovaSeqXp” workflow in “S4” mode flowcell. The Bcl to FastQ conversion was performed using bcl2fastq_v2.20.0.422 from the CASAVA software suite. The quality scale used is Sanger/phred33/Illumina 1.8+. Three biological replicates were sequenced per condition.

RNA libraries of the samples to select the final signature were constructed as described above, and samples were sequenced on HiSeq2500 (HiSeq Control Software 2.2.58/RTA 1.18.64) with a 1 × 51 setup using “HiSeq SBS Kit v4” chemistry. The Bcl to FastQ conversion was performed using None from the CASAVA software suite. The quality scale used is Sanger/phred33/Illumina 1.8+. One biological sample has been sequenced per condition.

### Bioinformatics

The RNASeq-derived counts and TPM normalized data were analysed using R 4.1.1 (R Core Team 2021) with the extension of the tidyverse [49]. For the transcriptomes from the six basal-like cell lines used to identify a robust Notch signature, gene symbols were annotated using Genecode v32 of the human genome assembly GRCh38. The genes annotated in an intersection of the datasets TCGA-BRCA, Oslo2 and METABRIC were selected, and lowly expressed genes (< 10 counts per gene), immunoglobulin gene segments, readthrough transcripts, ribosomal genes, sex-linked genes (Y chromosome and XIST), uncharacterized locations, uncharacterized long intergenic non-protein coding RNA and obsolete Entrez Gene IDs were excluded. Both eigencorplot, calculated for the first 20 principal components, and PCA plots were derived from variance-stabilized counts [50]. Differential gene expression analysis has been performed for the training cohort by applying generalized linear models using DESeq2 [51] with cell culture condition, timepoint, cell line background, library preparation batch, RIN number and library size as model covariates. For each contrast of interest, *p* value distributions of the results have been plotted, and Volcano plots generated by plotting ashr shrunken log2 fold changes [52]. Gene expression profiles of the cell lines were clustered using hierarchical clustering with complete linkage, where the Euclidian distance served as the distance metric to quantify the expression differences between each of the analysed cell lines.

For the 19-cell line dataset, which was used to select the final signature, gene symbols were annotated using Genecode v19 of the human genome assembly GRCh37, and genes from the gene universe of the training cohort were selected, and lowly expressed genes excluded (cpm < 1). As only one replicate per cell line had been analysed per treatment and timepoint, differential expression analysis was performed by fitting linear models with limma [53] and cell culture condition, timepoint, cell line subtype and library preparation batch as model covariates. Breast cancer subtypes were annotated as previously described [54]. A schematic pipeline for the data processing is provided in Additional file 1: Fig. S1.

### Calculation of signature scores

Signature scores were calculated estimating the mean of the gene-wise expression *Z*-scores of the trimmed mean of *M*-values (TMM) normalized expression values of the signature genes for the RNASeq-derived datasets and scaled log2 MFI for the microarray patient cohorts. Relative signature scores were calculated by the respective difference of signature scores at ground-state and treatment condition.

### Evaluation of gene expression signatures

The coherence score was evaluated by calculating the average gene-to-gene expression correlation for each number of genes in the candidate comparisons in the respective patient cohort, and the empirical *p* value has been derived from calculating the coherence score of 100,000 randomly chosen gene sets of a size between 3 and 100 genes from the same gene universe used to derive the differentially expressed genes. For visualization, the coherence scores and the empirical *P* values were plotted for signatures sizes between 3 and 50 and significance levels of *p* = 0.5, 0.05 and 0.0001 drawn [55, 56].

### Gene set enrichment analysis

Gene set enrichment analysis (GSEA) was performed both on the initial cohort by ranking the differentially expressed genes using the Wald statistic and on the second cohort by ranking the genes according to the −log10(*p* value)*log2 Fold Change [57]. The KEGG and Reactome pathway analysis were conducted as previously described by the Xia laboratory at McGill University: https://www.xialab.ca/tools.xhtml using *ExpressAnalyst* https://www.expressanalyst.ca/ [58].

### Gene set network centrality analysis

Gene set network centrality analysis was conducted utilizing the STRINGdb (version 12 for humans) and the igraph implementation in R. Gene signatures were mapped onto the STRING database, and the interaction network was extended by incorporating neighbouring genes, and all pair distances within this network were calculated, designating distances as "NA" for isolated genes or when the calculated distance was "Infinity”. The shortest path distances to CSL (selected as the most central hub for Notch signalling) from all other nodes were calculated and compared against a background distribution generated from 1000 random gene sets with sizes comparable to the tested gene signatures. Those gene sets served as a comparative baseline, allowing to distinguish whether the observed centrality was due to inherent network structures or a random occurrence. It also facilitated the calculation of the empirical p-value, computed as the proportion of random gene sets yielding a smaller mean distance to CSL than the actual gene signature and quantified the likelihood of observing a centrality score at least as extreme as the one obtained under the assumption of randomness.

### Receiver operating characteristic analysis

Receiver operating characteristic (ROC) and accuracy rates were calculated with the help of the R implementation pROC [59] with the relative signature score as predictor and the experimental condition as response.

### Analysis of signature scores in relation to Notch mutation status in the TCGA BRCA cohort

To assess the Notch mutation profile in the TCGA BRCA cohort as potentially activating or inactivating, we calculated a composite score integrating copy-number alterations, structural variants and mutations across the core set of NOTCH genes (NOTCH1-4, DLL1, DLL3, DLL4, JAG1 and JAG2; downloaded from cBioportal 15 October 2023). Patients were classified into three categories: "NOTCH activation alterations", "NOTCH inactivation alterations" or "NOTCH wild-type". A score of -1 was assigned to deleterious mutations (including truncating mutations, missense mutations and homozygous deletions; a score of + 1 to amplifications and a score of 0 to patients with no mutations, copy-number alterations or structural variations in Notch receptor or ligand genes (see [30] for review on Notch mutations in cancer). To assess the predictive power of the gene expression signatures, receiver operating characteristic (ROC) analysis was employed for three binary comparisons ("NOTCH activation alterations" versus "NOTCH wild-type"; "NOTCH wild-type" versus "NOTCH inactivation alterations" and "NOTCH activation alterations" vs. "NOTCH inactivation alterations"). The area under the ROC curve (AUC) together with its 95% confidence interval was then calculated for each signature. cBioportal annotations were conducted as previously described [60–62].

### The PROMIX and BEAUTY patient studies

The PROMIX trial (ClinicalTrials.gov identifier NCT00957125) and patients in this study have been previously described in detail [48]. In PROMIX, patients with newly diagnosed, locally advanced HER2 negative breast cancer were treated with neoadjuvant chemotherapy, six courses of epirubicin and docetaxel given every 3 weeks. In patients without a complete response after the first two cycles, bevacizumab was added to chemotherapy during cycles 3–6. The PROMIX study does not contain HER2 + patients. RNA was extracted from serial biopsies at baseline and cycle2 and surgical specimens (275 samples from 141 patients) and was then profiled on Illumina Human HT-12 v4.0 Expression BeadChip (Illumina Inc., San Diego, CA), as described previously (GSE87455) [48]. The GSVA algorithm [63] was employed to quantify Notch pathway score for each sample. Pair-wise patients’ scores were compared using Wilcoxon rank-sum test. Survival curves were constructed using the Kaplan–Meier method and compared with the log-rank test, where the optimal cutoff values of Notch pathway score were determined using the maximally selected rank statistics. The breast cancer genome-guided therapy study (BEAUTY) trial (ClinicalTrials.gov Identifier: NCT02022202) is a prospective study wherein 132 stages I–III breast cancer patients were treated with 12 weeks of weekly paclitaxel (with trastuzumab and pertuzumab for HER2 + tumours), followed by four cycles of an anthracycline-based regimen [64]. From the BEAUTY cohort, clinical response was classified at surgery as either non-responders (residual disease) or pCR (responders), and signatures were obtained at baseline and at surgery (for those with residual disease). RNA sequencing was performed as previously reported [64].

## Results

### An assay to identify candidate genes for a Notch transcriptomic signature

To identify a Notch transcriptional signature for breast cancer, we first set out to identify genes regulated by Notch signalling in a breast cancer context. To this end, we established an assay in which modulation of the level of Notch signalling was transcriptomically read out in a panel of six basal-like cell lines (BT20, HCC1187, HCC1599, HCC70, MDAMB436 and MDAMB468) that express NOTCH receptors (Additional file 1: Fig. S2A). The cell lines were cultured and subjected to different levels of Notch activation: Elevated Notch signalling (“Notch on”) was accomplished by culturing cells on immobilized Jagged1 ligand, as Jagged1 has been implicated in basal-like breast cancer [17, 19, 20]; blockage of Notch signalling (“Notch off”) was achieved by culturing the cells in the presence of the *γ*-secretase inhibitor DAPT and DMSO was used for the “ground state” of Notch signalling (see Fig. 1B for a schematic presentation of the assay). The six cell lines were analysed by bulk transcriptomics at 8- and 72-h post-activation/inhibition to capture immediate as well as more long-term, possibly secondary, gene expression differences resulting from Notch modulation (Fig. 1B). The efficacy of activating or blocking Notch signalling was validated by qPCR expression analysis of the canonical Notch target genes HES1 and NRARP [65, 66]. In all six cell lines analysed, the expected up- and downregulation of at least one of the well-established Notch target genes HES1 and NRARP in each cell line was observed after 8 and/or 72 h (Fig. 1C, Additional file 1: Fig. S2B), confirming the validity of the assay.

In a principal component analysis (PCA) dimensional reduction of the transcriptomic data, the cell lines cluster distinctly from each other (Fig. 1D), and the activation or blockade of Notch signalling did not reset the entire transcriptomic landscape but induced more subtle differences. The main source of variance is thus transcriptomic differences between the cell lines, followed by treatment time, while the effect of Notch modulation is smaller, and significantly correlates to principal component 6, 7 and 10, which represent 3.5%, 2.1% and 0.5% of the total variance in the dataset (Fig. 1E, Additional file 1: Fig. S2C); for differences between the Notch-on, ground-state and Notch-off conditions, see Additional file 1: Fig. S2D. In sum, we have established an assay allowing us to identify Notch-induced transcriptomic differences in basal-like breast cancer cell lines and to establish candidate genes for a Notch signature.

### Identification of a robust and coherent 20-gene transcriptomic Notch signature

**Fig. 2:**
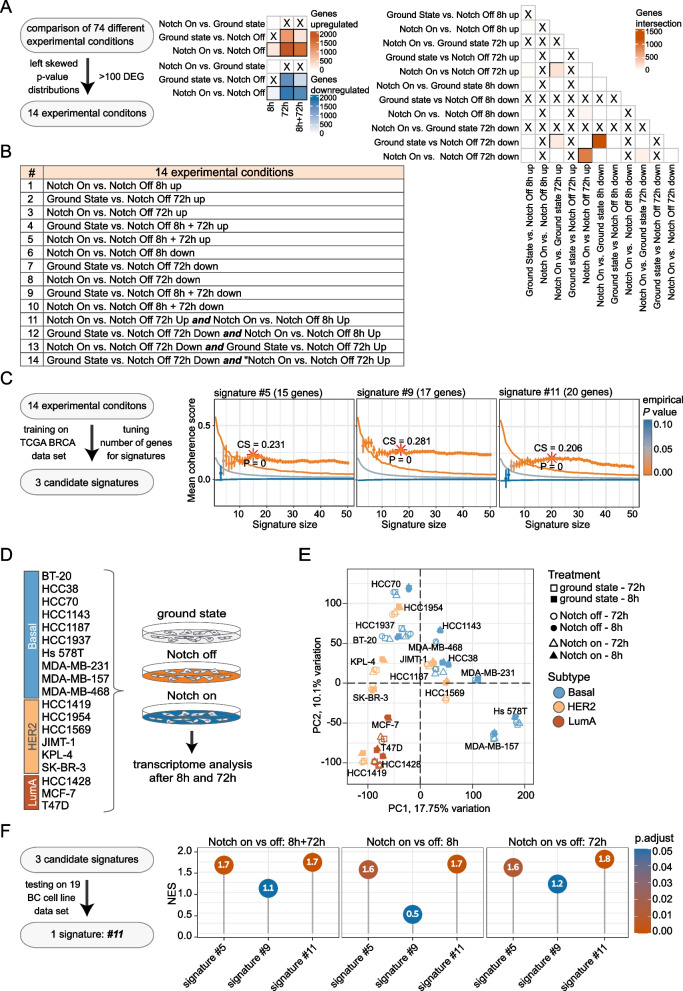
Identification of a 20-gene Notch transcriptomic signature. **A** Heatmap to represent the number of differentially expressed genes (DEGs) for all possible comparisons of the experimental conditions tested: NOTCH activation status: “Notch on”, “ground state” and “Notch off”; experimental timepoint: 8 h or 72 h (8 h or 72 h), or all samples analysed for differential gene expression analysis together, irrespectively of the experimental timepoint (8 + 72 h). Upregulated genes in brown; downregulated genes in blue. To the right: DEGs for various combinations of datasets as indicated. Gene intersections indicate the number of DEGs; a cross indicates comparisons with not-left skewed P value distributions or less than 100 DEGs. up = upregulation and down = downregulation*.* **B** The list of 14 candidate experimental conditions after left skewed *P value* analysis of the initial 74 experimental conditions. **C** Plots of coherence scores for three selected candidate signatures (signatures #5, #9 and #11) for different number of genes in each signature. The data show signature scores > 0.2 for the three signatures after training for optimal coherence scores for signature sizes between 10 and 30 genes on the TCGA-BRCA patient dataset. **D** Schematic depiction of generation of bulk transcriptomic datasets from 19 breast cancer cell lines (10 basal-like; 6 HER2-posivite and 3 Luminal A (LumA)). Transcriptomes were obtained after “Notch on”, “Notch off” or “ground state” conditions, as for the six cell lines in Fig. 1. **E** Principal component analysis of the transcriptomic data from the 19 cell lines under “Notch on”, “Notch off” or “ground state” conditions at 8 or 72 h, as indicated. A total of 12,038 genes were used for the PCA. **F** GSEA normalized enrichment score representations of the three candidate signatures in the transcriptomic dataset from the 19 cell lines in E tested against transcriptomes from 8, 72 or 8 + 72 h. Note that signature #11 outperforms the other signatures across the different datasets. For a corresponding analysis by ROC curves of the candidate signatures to classify Notch activation status in the 19-cell line dataset, see Additional file 1: Fig. S3E

With the datasets from activation or blockade of Notch signalling at the 8- and 72-h timepoints at hand, we next searched for differentially expressed genes (DEGs) between all possible conditions and timepoints of the six cell lines. As Notch signalling leads both to increased and decreased expression of downstream genes [67], we considered DEGs and potential signatures composed of either up- or downregulated genes across the different experimental points. Of the 74 possible comparisons, we considered only differential expression results with a left skewed *P* value distribution and a minimum of 100 DEGs (Fig. 2A), resulting in 14 selected comparisons with differentially expressed hits (Fig. 2B, and for the full list of differentially expressed genes, see Additional file 3: Table S2).

Gene expression signatures are often derived from methodical examination of samples under controlled conditions or from large-scale genomic cancer studies with the goal of pinpointing coordinated gene expression patterns. These are indicative of a systematic regulation or functional linkage among genes that relate to the conditions. Various approaches have been developed to generate gene expression signatures and their scores, among them penalized regression methods [68], cluster approaches like the weighted gene co-expression network analysis (WGCNA) [69] and ranked lists of differentially expressed genes [70]. Various strategies exist for calculating gene signature scores. One can simply average the expression values of the signature genes [71], apply single-sample gene set enrichment analysis (ssGSEA) [72], gene set variation analysis (GSVA) [73] or more complex approaches using gene weights or regression models. The underlying hypothesis is that a robust signature will maintain consistent correlations or relationships, irrespective of the dataset in question. If a signature is genuinely reflective of significant biological insights, then the expression profiles it comprises should manifest uniform patterns across diverse datasets. The coherence score evaluates the reproducibility of a signature by assessing the coherent (e.g. the pair-wise correlation) expression of its genes and has been used in several contexts [65]. For a signature of size n, the score ranges from 1 to $- \frac{1}{n - 1}$ and a high absolute score indicates a strong positive or negative relationship between the signature genes. Similarly, a high coherence score results in a convergence of signature scores, independent from the scoring approach that was used [56]. To the best of our knowledge, there is no other algorithm that can computationally evaluate the significance of a signature. It is, however, important to note that a high coherence score does not prove a causal relationship with the originally proposed phenomena [74], which is why a comprehensive cross-validation or comparative approaches are required [56].

To establish the optimal signature length, we calculated the average gene-to-gene correlation (coherence score) for each number of genes in the candidate comparisons in the TCGA-BRCA patient cohort as a training cohort [55, 56] (Additional file 1: Fig. S3A). The optimal number of genes within each signature was selected based on both the largest coherence score as well as smallest empirical *P* value with the smallest gene set size in the range between 10 and 30 genes. This analysis revealed three signatures having a coherence score > 0.2 [56]: signature #5 (15 genes); signature #9 (17 genes) and signature #11 (20 genes) (Fig. 2C; Additional file 1: Fig. S3A, B and Additional file 4: Table S3), and the three signatures were selected for further analysis.

To explore the accuracy and robustness of the three signatures further, we generated a novel transcriptomic dataset using the same principal experimental set-up for activation (Jagged1) and blockade (DAPT) of Notch signalling as described above, but this time from a larger panel of 19 breast cancer cell lines (Fig. 2D). The panel included 10 basal-like cell lines (BT-20, HCC38, HCC70, HCC1143, HCC1187, HCC1937, Hs-578T, MFA-MB-231, MDA-MB-157 and MDA-MB-468), three cell lines from Luminal A (HCC1428, MCF-7 and T-47D) and six cell lines from HER2-enriched (HCC1419, HCC1954, HCC1569, JUMT-1, KPL-4 and SK-BR-3) breast cancer (Fig. 2D). The transcriptomes from the 19 cell lines at ground state, i.e. without perturbation of Notch signalling, revealed that basal-like, Luminal A and HER2-enriched cell lines largely clustered distinctly according to tumour subtype in a PCA analysis (Fig. 2E). As for the transcriptome analysis of the six basal-like cell lines (Fig. 1D), the transcriptomic differences induced by elevated or reduced Notch signalling were smaller in comparison with the cell line-specific differences (Fig. 2E; for differences between the Notch-on, ground-state and Notch-off conditions, see Additional file 1: Fig. S3C). As a quality control, the transcriptomes from the 19 cell lines in ground-state conditions were compared to previously established transcriptomes from the same cell lines [75], and unsupervised clustering demonstrated a good correlation for all cell lines (Additional file 1: Fig. S3D), corroborating the validity of the experimental set-up. All cell lines also expressed at least one of the four Notch receptor genes (Additional file 1: Fig. S3E), suggesting that they may potentially be activated by immobilized ligand.

We next used both gene set enrichment analysis (GSEA) and receiver operating characteristic (ROC) curve analysis to test the performance of the three signatures on the transcriptomes from the 19 cell lines. Signature #11, derived from the intersection of upregulated DEGs from the 8- and 72-h timepoints for Notch on vs Notch off, consistently outperformed signatures #5 and #9 in the GSEA analyses from three different conditions (on the DEGs of the treatment conditions 8-h Notch on vs Notch off; 72-h Notch on vs Notch off and Notch on vs Notch off irrespectively of the timepoint, Fig. 2F). ROC curve analysis revealed that signature 11 showed the best and most robust performance for both timepoints in comparison with the other candidate signatures when classifying the relative response to the experimental activation or inhibition of the Notch pathway (AUC = 0.86 for all cell lines and timepoints, AUC = 0.86 for cell lines treated only for 8 h and AUC = 0.87 for 72 h treated cell lines) (Additional file 1: Fig. S3 F and G; for full list of gene set enrichment scores see Additional file 5: Table S4a and for AUC values of the ROC curves see Additional file 6: Table S4b). Collectively, signature #11, based on the data from training on the TCGA-BRCA patient dataset and from an independent Notch signalling-modulated cell line dataset, showed the best performance and was, therefore, used in the subsequent analyses. A flow chart for the pipeline leading to the 20-gene transcriptomic Notch signature is presented in Additional file 1: Fig. S1, and the code is provided at GitHub: https://github.com/KarolinskaMerck/NotchBRCASignature.

### The nature of the 20-gene transcriptomic Notch signature

**Fig. 3:**
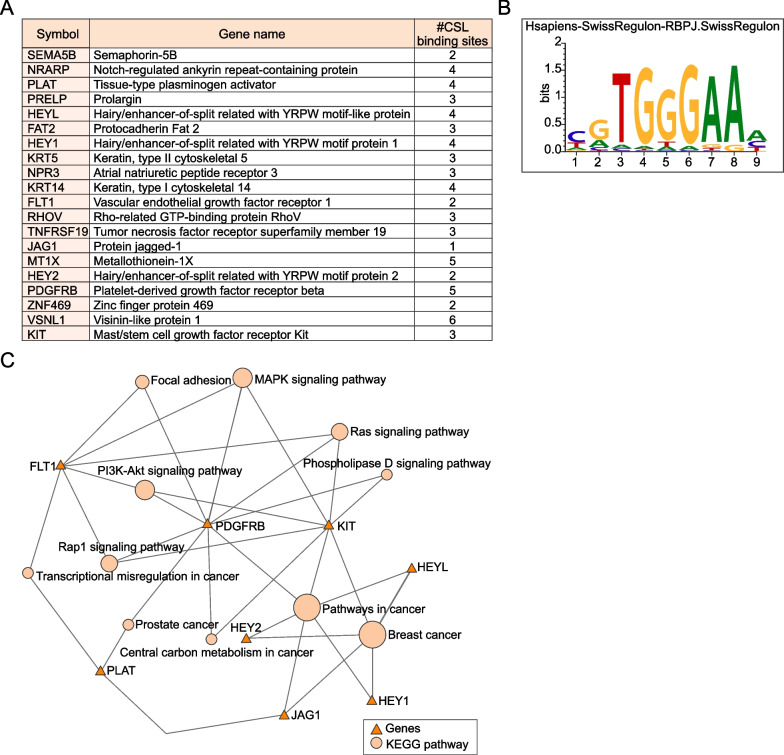
The 20-gene Notch transcriptomic signature. **A** A list of the 20 genes in the transcriptomic signature along with the number of CSL-binding sites in their promoter regions (defined as 3000-base pair (bp) up- and 500-bp downstream of the transcriptional start site (TSS)). **B** The CSL-binding site defined from the SwissRegulon database.** C** Gene enrichment map of genes in the 20-gene signature and their links to search terms in KEGG pathways. For a corresponding analysis of the REACTOME and string.db databases, see Additional file 1: Fig. S4

The selected transcriptomic signature #11 comprises the following 20 genes in rank order: *SEMA5B, NRARP, PLAT, PRELP, HEYL, FAT2, HEY1, KRT5, NPR3, KRT14, FLT1, RHOV, TNFRSF19, JAG1, MT1X, HEY2, PDGFRB, ZNF469, VSNL1 and KIT* (Fig. 3A). The *HEY1*, *HEY2*, *HEYL*, *NRARP*, *PDGFRB*, *JAG1, NPR3, FLT1 and PRELP* genes represent functionally validated Notch downstream target genes [66, 76–81]. All 20 genes contained at least one CSL-binding site (from the SwissRegulon database; https://swissregulon.unibas.ch/sr/ (Fig. 3B) in the promoter region defined as 3000-base pair (bp) up- and 500-bp downstream of the transcriptional start site (TSS)) (Fig. 3A), and 17 of the genes (*SEMA5B, NRARP, PRELP, HEYL, FAT2, HEY1, KRT5, NPR3, KRT14, FLT1, RHOV, TNFRSF19, JAG1, MT1X, HEY2, PDGFRB and ZNF469*) have previously been identified as CSL targets in ChIP-seq using cell lines from different models [79, 82–84]. When the genes were plotted in a KEGG graph-network a number of the genes in the signature (*JAG1*, *HEY1*, *HEY2* and *HEYL*) linked to the term “breast cancer”, while the same four genes plus *PDGFRB* and *KIT* linked to the term “pathways in cancer” (Fig. 3C; for full list of enriched pathways, GO terms and detailed CSL binding site information, see Additional file 7: Table S5). Corresponding Reactome graph-network analysis linked several of the genes to Notch-related pathway terms (Additional file 1: Fig S4A, Additional file 7: Table S5). String.db interaction enrichment analysis indicated that the proteins are at least partially biologically connected (avg. node degree 2.1, avg. local clustering coefficient 0.479) and linked to functionally enriched Notch or disease-related pathway terms (Additional file 1: Fig S4B, Additional file 7: Table S5). In conclusion, the data show that the 20 genes in the Notch signature may represent immediate Notch downstream genes and that several of the genes relate to different disease terms, such as breast cancer or to Notch in pathway enrichment analyses.

### Validation of the 20-gene Notch transcriptomic signature in two independent breast cancer datasets

With the 20-gene signature at hand, we explored whether it would show good coherence scores in two other breast cancer patient datasets: the METABRIC [85, 86] and Oslo2 [87] patient cohorts. The signature showed significant coherence scores in both METABRIC (0.16) and Oslo2 (0.16) with an empirical *p* value < 10^–5^ (Table 1 and Additional file 4: Table S3). The mean coherence score for the 20-gene signature was higher than for signatures #5 and #9, which showed coherence scores of 0.12 and 0.12 (signature #5) and 0.10 and 0.19 (signature #9), in keeping with the 19-cell line transcriptomic dataset (see Fig. 2F). Together, these data demonstrate that the 20-gene signature exhibits a robust coherence score also when validated in other, independent, breast cancer datasets.

**Table 1:** Validation of the 20-gene signature in the METABRIC and Oslo2 datasets

Signature name	Signature size	CS TCGA	CS METABRIC	CS Oslo2
Signature #5	15 genes	0.23	0.12	0.12
Signature #11	20 genes	0.2	0.16	0.16
Signature #9	17 genes	0.28	0.1	0.19

### Comparing the 20-gene Notch transcriptomic signature to previously established signatures

There have been previous efforts to establish Notch transcriptomic signatures in various physiological settings, including breast cancer, and we were next interested in comparing our 20-gene signature to already published signatures. For example, Notch-responsive gene sets have been established from myogenic cells [79], pericytes [88], keratinocytes [82], myeloid cells [89] and endothelial cells [90]. From the cancer field, Notch-responsive gene sets have been established from T-cell acute lymphoblastic leukemia and mantle cell lymphoma [91–93]. In addition, dedicated efforts to produce Notch signatures have been made from cells in the myelomonocytic lineage [89], from small B-cell lymphoma [94] and from a combined analysis of breast cancer, T-ALL and mantle cell lymphoma cell lines [95]. Additional Notch signatures have been generated and collected in the Molecular Signatures Database (MSigDB version 7.2) [70, 96] and were identified searching for the term NOTCH in the signature name. Signatures of a size between 3 and 100 genes were included for analysis, which qualified 15 of the 20 signatures identified by a literature search. From the MSigDB signature database with the term NOTCH in the name of the signature, 42 of 51 signatures remained. These 55 NOTCH-related signatures are shown in Additional file 8: Table S6a, and as control, we included two signatures that are related to Notch “in name only”, but instead stem from pelvic research (“sciatic Notch”) and thus should not be related to the Notch signalling pathway.

We calculated the mean overlap between the signatures and assessed mean similarity using Jaccard coefficient and a subset of commonly used gene sets, such as KEGG, WP Notch signalling pathway and REACTOME SIGNALLING BY NOTCH1-4. Expectedly, the Notch receptor-specific REACTOME datasets clustered more closely together, while the 20-gene signature, along with several other previously published Notch transcriptomic signatures, was more distantly related to the other gene sets (Additional file 1: Fig. S5A–E). The two “sciatic Notch” signatures as expectedly showed the lowest degree of similarity to the other signatures (Additional file 1: Fig. S5A–D, Additional file 9: Table S6b). We were next interested in taking a more gene-centric approach and exploring how centrally the genes in the 20-gene signature were positioned in the string.db protein–protein interaction network in relation to CSL, set as the central node of Notch signalling because of its central role for all canonical Notch signalling. The 20-gene Notch signature produced a mean signature score of 1.72, while the BIOCARTA NOTCH PATHWAY signature was most central to CSL (centrality score of 0.83), and the Abnormal Greater Sciatic Notch Morphology signature as expected was found to be most distant to CSL (centrality score of 2.37). The centrality score for the 20-gene signature was also significantly smaller than the centrality score for a random selection of genes (Additional file 1: Fig. S5F, Additional file 10: Table S6c).

We performed GSEA and ROC curve analyses [49] for the published signatures and the 20-gene signature both on the differentially expressed genes from the six basal-like cell line experiment used to train the 20-gene Notch transcriptomic signature and on the larger panel of 19 cell lines. Twenty of the 57 published signatures were significantly enriched by GSEA on the differentially expressed genes of all experimental timepoints (*p*.adj < 0.05), and 18 could classify the treatment conditions with an AUC > 0.7 in the six-cell line training cohort. Thirty-seven of the 57 published signatures were significantly enriched in the larger 19-cell line panel, and eight showed an AUC > 0.7 (Additional file 1: Fig S6A–E and Additional file 11: Table S7, Additional file 6: Table S4b). When performing both GSEA and ROC curve analyses on either of the experimental timepoints separately, we observed that many signatures showed worse performance to classify cell lines correctly after 72-h treatment in comparison with after 8 h of treatment.

**Fig. 4:**
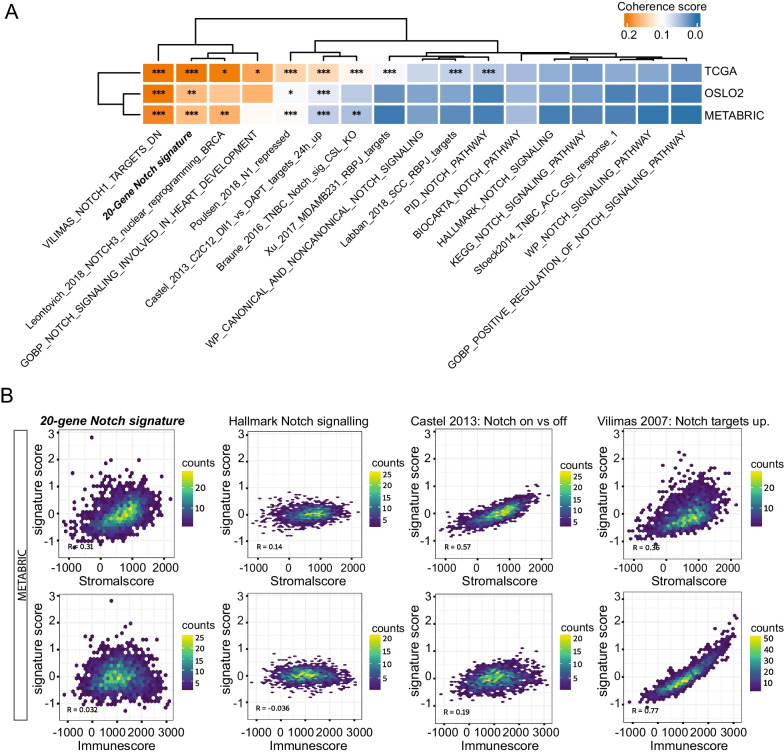
Comparing the 20-gene signature to previously established Notch signatures. **A** Coherence score and empirical *P* value calculated for the 20-gene signature along with a selection of previously established Notch signature in all three patient cohorts, as indicated. **B** Inferred immune and stromal content in the METABRIC dataset for the 20-gene signature and previously published Notch signatures, as indicated

We estimated the quality of the published signatures by calculating coherence score [55], as well as the empirical *P* value [56] in all three patient cohorts (Additional file 12: Table S8). In the TCGA-BRCA, METABRIC and Oslo2 datasets, nine, four and seven of the 57 analysed published signatures had coherence scores > 0.12, respectively. The adjusted empirical *p* value was < 0.0001 in 10, nine and six signatures in the TCGA, METABRIC and Oslo2 signatures, respectively. The “NOTCH1 targets down” signature by Vilimas et al. [93] showed higher coherence scores than the 20-gene signature (TCGA-BRCA = 0.28, METABRIC = 0.19 and Oslo2 = 0.22) (Fig. 4A, Additional file 1: Fig S7A). In addition, we analysed the PAM50 subtypes specific coherence scores in the three datasets, and most of the published signatures had low coherence scores (< 0.12) in the basal-like subtype (see Additional file 13, 14: Table S9; Signatures with CS > 0.12: TCGA: 7, METABRIC: 5, Oslo2: 5).

An important question is whether tumour purity, stromal and immune infiltration had an impact on the nature of the various gene signatures. To address this, we inferred the immune and stromal content using the ESTIMATE method [97]. Most signatures, including the 20-gene signature reported here, did not show significant correlation with the Immunescore or Stromalscore, but the signatures by Vilimas et al. and Klinakis et al. [89, 93] were highly correlated with immune infiltration, whereas two of the other signatures [79, 90] correlated with stromal content in all tested patient cohorts (Fig. 4B and Additional file 1: Fig. S7B; for full list of purity data, see Additional file 15: Table S10).

As expression of various genes in the Notch signalling pathway often has been used as a proxy for the level of Notch activation, we were also interested in exploring how the signature score from 20-gene signature correlated with expression of genes in the Notch pathway. To address this, we constructed a new gene expression signature, called the “Notch core signature”, and which is composed of the genes encoding the four Notch receptors (Notch 1–4) and the five Notch ligands (Jagged 1 and 2; Dll 1, 3 and 4) (Additional file 1: Fig. S8A and B). When comparing the signature scores in the dataset from the 19 cell lines (ground state) as well as in the TCGA, METABRIC and Oslo2 patient cohorts, there was a positive correlation score for both the cell line and the patient datasets (Additional file 1: Fig. S8A, B, Additional file 16: Table S11a). Several of the previously published signatures similarly showed a positive correlation score, while some signatures failed to reach a significant correlation score (Additional file 1: Fig. S8A and B). As a complementary approach, we were curious whether the 20-gene Notch signature classify patient samples with genomic alteration in the Notch pathway. To this end, we identified patients from the TCGA cohort that carried mutations likely to affect Notch signalling and calculated a composite score integrating copy-number alterations, structural variants and mutations or truncations across Notch receptor and ligand genes (NOTCH1-4, DLL1, DLL3, DLL4, JAG1 and JAG2). This score aimed to reflect the cumulative impact of genetic aberrations on pathway activity, assuming that such a composite measure would be more indicative of the pathway's functional status than individual alterations. To assess the predictive power of the 20-gene signature and previously described signatures, we employed ROC analysis for two binary comparisons ("NOTCH activation alterations" vs. "NOTCH wild-type", "NOTCH wild-type" versus "NOTCH wild-type" and "NOTCH activation alterations" vs. "NOTCH inactivation alterations") and calculated the area under the ROC curve (AUC) together with its 95% confidence interval for each signature. The ROC analysis revealed a low, but consistent, degree of classification accuracy for the 20-gene signature with an AUC of 0.56 (0.52–0.61) when distinguishing between samples with “Notch-core activating alterations” and “Notch-core wild-type” classifications (Additional file 1: Fig S8C; signature score changes are shown in S8D, Additional file 17: Table S11b). In conclusion, the 20-gene signature presented here scores well in all tests and validation steps, and in most situations outperforms previously described signatures.

### The 20-gene signature shows a higher signature score in basal-like breast cancer cell line and patient datasets

**Fig. 5:**
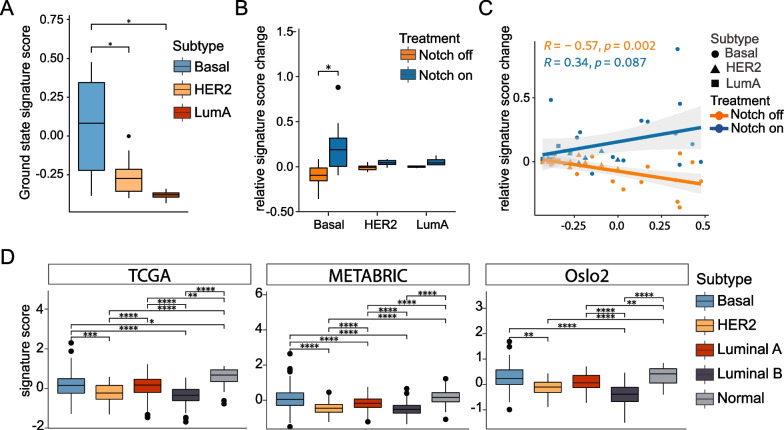
The signature score for the 20-gene signature in different subtypes of breast cancer. **A** Analysis of signature scores at the ground state in basal-like, HER2 and Luminal A (LumA) cell lines from the 19-cell line transcriptomic dataset. **B** Analysis of the relative signature score in the 19-cell line dataset under “Notch on” and “Notch off” conditions, as indicated. **C** The relative signature score changes under “Notch on” (blue) and “Notch off” (orange) conditions. **D** Analysis of signature scores for the 20-gene signature in basal-like/TNBC (Basal), HER2, Luminal A (LumA) and Luminal B (LumB) breast cancer, in the TCGA, METABRIC and Oslo2 datasets, as indicated. (*p < 0.05; **p < 0.001; ***p < 0.0001 and ****p < 0.00001)

Having validated the 20-gene signature for coherence score in patient and cell line datasets, we next used the signature to learn whether different breast cancer subtypes were endowed with different levels of Notch signalling. We first revisited the transcriptome analysis from the 19 cell lines representing basal-like, Luminal A and HER2-enriched subtypes. The absolute signature scores at ground state were significantly higher in the basal-like cell lines (Fig. 5A), and the relative signature score difference in relation to the ground state when comparing “Notch on” versus “Notch off” conditions was significant in the basal-like cell lines (Fig. 5B). Furthermore, the relative signature score increased under “Notch on” conditions and decreased under “Notch off” conditions, as compared to their ground-state signature scores (Fig. 5C, Additional file 18: Table S12). We next investigated signature scores in relation to the PAM50 molecular subtype classification [97] for the 20-gene signature in the three patient datasets. In both the TCGA, METABRIC and Oslo2 datasets, the mean signature score was highest in basal-like/TNBC and normal-type breast cancer, followed by the Luminal A subtype and with HER2 and Luminal B subtypes exhibiting lower signature scores (Fig. 5D). When the other signatures discussed above were tested in the same manner, they also largely showed higher scores for basal-like and normal subtypes (Additional file 1: Fig. S9, Additional file 13, 14: Table S9). Together, the results reveal a higher signature score in the basal-like subtype.

### Analysis of the 20-gene signature score in response to breast cancer therapy in the PROMIX and BEAUTY breast cancer cohorts

**Fig. 6:**
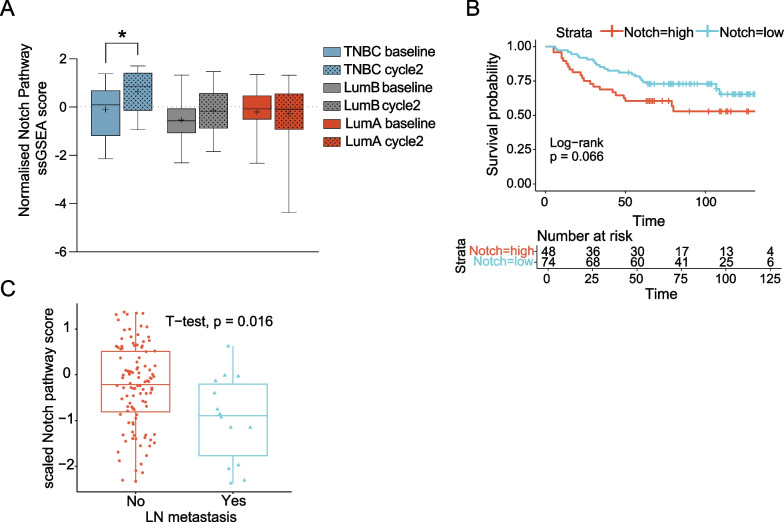
Analysis of the 20-gene signature score in the PROMIX breast cancer cohort. **A** The 20-gene signature score (ssGSEA) for three breast cancer subtypes (20 Luminal A, 27 Luminal B and 21 TNBC patients) at the onset (orange) and after two rounds of epirubicin and docetaxel treatment (blue). **B** Disease-free survival analysis (survival probability) of patients (*n* = 122 (28 TNBC; 42 LumA; 42 LumB)) according to Notch signature score (high (red) and low (blue) groups) at baseline. **C** Signature score in the primary tumours for TNBC patients with (Yes) or without (No) lymph node metastasis. (*p < 0.05)

To gain insights into how the level of Notch signalling is altered in response to breast cancer therapy and therapy outcome, we first analysed data from the PROMIX trial [98]. For a subset of patients with radiographic evidence of residual disease after two cycles of epirubicin and docetaxel (21 luminal A, 27 luminal B and 21 TNBC patients) and with paired biopsies available for transcriptomic analysis, the signature score (ssGSEA) increased after treatment in the TNBC patient group (*p* value for pair-wise Wilcoxon rank sum, 0.03) and to some extent in the Luminal B group (*p* = 0.10), while there was no increase in the Luminal A patient group (*p* = 0.86) (Fig. 6A). Furthermore, the highest baseline Notch signature, i.e. before the onset of treatment, was observed in the TNBC patients (Fig. 6A), in keeping with the results presented in Fig. 5 (for signature scores for previously published signatures, see Additional file 1: Fig. S10A; Additional file 19: Table S13). Patients with a lower Notch signature score at baseline (*n* = 74, scaled GSVA score < − 0.03) showed a tendency to better disease-free survival (DFS) than those with higher Notch signature score (*n* = 48; log-rank *p* = 0.066) (Fig. 6B), and this was also the case for the Notch core signature, while in contrast, for example, the Leontovich et al. signature [21] showed the opposite correlation (Additional file 1: Fig. S10B). Patients with lymph node metastasis (*n* = 14; 1 Luminal A, 8 Luminal B, 5 TNBC) showed a lower signature score in their primary tumours prior to treatment in comparison with those without lymph node metastasis (*n* = 108; 39 Luminal A, 45 Luminal B, 23 TNBC, 1 non-classified; *p* = 0.016) (Fig. 6C and Additional file 1: Fig. S10C).

**Fig. 7:**
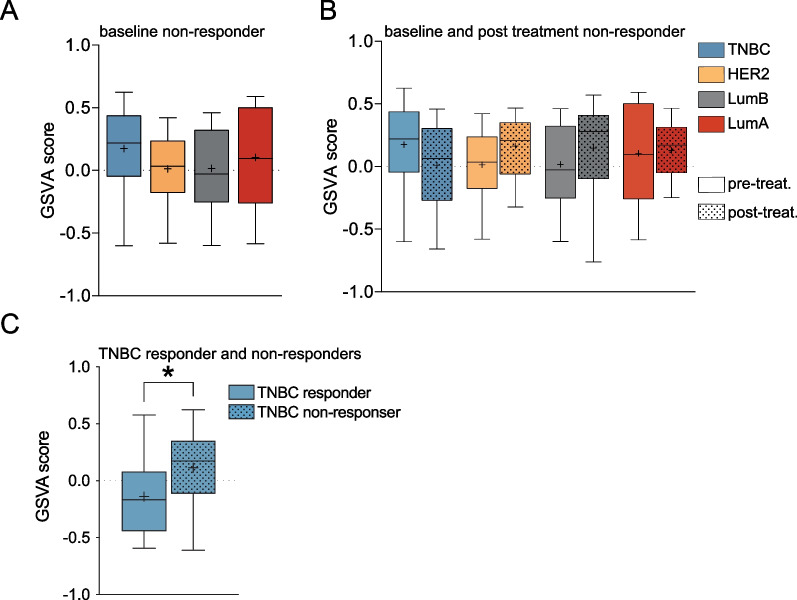
Analysis of the 20-gene signature score in the BEAUTY breast cancer cohort. **A** The 20-gene signature score analysed as gene set variation analysis (GSVA) in TNBC, HER2, Luminal A (LumA) and Luminal B (LumB) patients for the non-responder group before treatment from the BEAUTY cohort. **B** Analysis of the signature score before or after neoadjuvant chemotherapy (paclitaxel and anthracycline) for the non-responder patient group in A, as indicated in the figure. **C** The baseline pre-treatment GSVA score for TNBC responders and non-responders as indicated. (*p < 0.05)

We next addressed the level of Notch signalling in response to therapy in the prospective breast cancer genome-guided therapy study (BEAUTY) [64]. The 20-gene signature score, evaluated by gene set variation analysis (GSVA), was highest in the baseline TNBC subtype, followed by the Luminal A, HER2 + and the Luminal B subtypes (Fig. 7A). When comparing the GSVA at baseline and at surgery, the GSVA values increased in the HER2+, Luminal A and Luminal B patients, while there was a decrease in the TNBC group (Fig. 7B). When analysing the GSVA signature pre-treatment score in therapy responders versus non-responders in the TNBC group, the non-responder group showed a higher GSVA value prior to the onset of the treatment (Fig. 7C). Similarly, most previously published signatures, as well as the Notch core signature, produced a higher signature score in the non-responder group (Additional file 1: Fig. S10D, for comparison of the signature scores for the various signatures in the pre- and post-treatment groups, see Additional file 1: Fig. S10E, Additional file 19: Table S13).

Taken together, the data from the PROMIX and BEAUTY cohorts suggest that TNBC tumours prior to therapy have a higher level of Notch signalling, and that in certain cases, chemotherapy may induce an increase in Notch signalling, although the outcome in TNBC and Luminal A patients differed between the two cohorts in this regard. The data generally suggest that elevated Notch signalling at baseline may be prognostic, as it is associated with worse disease-free survival in the PROMIX cohort. Furthermore, in the BEAUTY cohort, patients with TNBC and residual disease after NAC showed a higher Notch signalling score in their pre-treatment tumours.

## Discussion

Dysregulated Notch signalling plays a role in breast cancer initiation and development [6], and development of Notch-targeting therapies is a very active area of research [41, 42]. To be able to accurately monitor the level of Notch signalling as part of therapy evaluations is, therefore, important. To gain insights into the level of Notch signalling activation will also be important to select patients for future Notch-targeting therapies and to understand whether conventional and currently used breast cancer therapies affect Notch signalling, which may be an unwanted side effect. To address the need for a tool to monitor Notch signalling, we describe in this report the identification of a 20-gene Notch transcriptomic signature (*NRARP*, *SEMA5B*, *PLAT*, *PRELP*, *HEYL*, *FAT2*, *HEY1*, *NPR3*, *KRT5*, *FLT1*, *KRT14*, *RHOV*, *TNFRSF19*, *JAG1*, *MT1X*, *HEY2*, *PDGFRB*, *ZNF469*, *VSNL1* and *KIT*), which shows a very robust coherence score for breast cancer. To arrive at this signature, we first identified genes showing a significant transcriptomic response to modulation of Notch signalling in six basal-like cell lines, by performing an analysis of comparisons with left skewed *P value* distributions and at least 100 DEGs. This resulted in 14 candidate comparisons from originally 74 possible comparisons. Through a machine learning approach assessing coherence scores for various gene combinations, using the TCGA-BRCA patient dataset as the training dataset, three candidate signatures were identified. When the three signatures were tested against transcriptomes from 19 different breast cancer cell lines where the level of Notch signalling had been experimentally modulated, the 20-gene signature (signature #11) emerged as the best signature. Subsequently, the 20-gene signature was validated in two other, independent datasets: the METABRIC and Oslo2 datasets.

Nine of the 20 genes (*HEY1*, *HEY2*, *HEYL*, *NRARP*, *PDGFRB*, *JAG1, NPR3, FLT1 and PRELP*) represent functionally validated Notch downstream target genes [66, 76, 77, 79, 81, 99], while the others have not been proven to be Notch target genes, i.e. genes with altered expression in response to experimentally modulated levels of Notch signalling. Some of the genes, such as *VSNL1*, *SEMA5B* and *TNFSR19*, have been shown to be upregulated upon genetic removal of CSL, indirectly linking them to Notch signalling [76]. The notion that all 20 genes harboured CSL-binding sites in proximity to their transcription start sites suggests that all genes in the signature are direct Notch downstream genes, and for most genes, CSL binding to the promotor of the genes of the identified signature has indeed been demonstrated in chromatin immunoprecipitation (ChIP-seq) studies [79, 82–84]. Upregulation of JAG1 and FAT2 has furthermore been linked to poor prognosis in breast cancer [17, 100], and a Jagged-1/Notch2/PDGFR stroma–epithelial mechanism has been described in a poor prognosis fibroblast subset [39]. It is, however, of note that the signature does not contain some genes that previously have been implicated as Notch downstream genes, such as *c-MYC* [101–104]. Expression of c-MYC was significantly increased upon elevation of Notch signalling, but not to an extent that c-MYC met the formal criteria for inclusion in the signature.

The 20-gene signature compares well with previously generated Notch signatures for basal-like breast cancer and when it comes to the more long-term (72 h) induction in cell lines, which may be of relevance for assessing Notch signalling in patients, who likely have been exposed to long-term Notch activation. A signature developed from T-ALL transcriptomes [93] displayed a very good coherence score for the TCGA BRCA cohort (0.323) and the other two datasets (METABRIC: 0.140, Oslo2: 0.193) some of the other signatures likewise showed acceptable coherence scores (REACTOME_NEGATIVE_REGULATION_OF_NOTCH4_SIGNALLING = 0.313 TCGA-BRCA, 0.119 METABRIC, 0.218 Oslo2; Leontovich_2018_NOTCH3_nuclear_reprogramming_BRCA = 0.195, 0.167 METABRIC, 0.150 Oslo2). Furthermore, some of the previous signatures, including the Vilimas et al. and the Klinakis et al. signatures [89, 93], were strongly correlated to an immune cell rich stroma. In keeping with this notion, the Vilimas et al. signature did not show the expected response to the Notch-on and Notch-off conditions when challenged against the transcriptomic data from our cell line experiments.

The 20-gene signature showed the highest signature score for the basal-like and normal subtypes of breast cancer, followed by Luminal A, while the scores for Luminal B and HER2 + breast cancer were lower in the datasets from the TCGA, METABRIC and Oslo2 breast cancer patient cohorts. Similarly, the signature was highest in the TNBC subgroup in the pre-treatment samples from the PROMIX and BEAUTY cohorts. This corroborates the view that the Notch signalling level is comparatively higher in basal-like than in Luminal B and HER2 + breast cancer [105, 106]; for review see [107]. There were, however, also some signature score differences between the PROMIX and BEAUTY cohorts; the signature score for luminal A is, for example, almost at par with TNBC in PROMIX, while there is it considerably lower than TNBC in the BEAUTY cohort at baseline, i.e. prior to treatment. It is also of note that the analysis of 16 previously published signature revealed that some but not all the 16 previous signatures showed signature score distributions similar to the 20-gene signature across the various patient cohorts as well as in the cell lines (Additional file 1: Fig. S10 A, D and E). Also, we found a positive correlation score between the 20-gene signature and the “Notch core signature”, which reads out expression levels of Notch receptors and ligands. Notably, for both the 20-gene signature and the Notch core signature, a high signature score correlated with a worse outcome in terms of disease-free survival in the PROMIX study, whereas, for example, a poor outcome was correlated with a low signature score from the Leontovich et al. signature [21].

Regarding the signature score in response to neoadjuvant chemotherapy (NAC), analysis of the BEAUTY residual disease remaining after 20 weeks of taxane, anthracycline and cyclophosphamide therapy (along with HER2 directed therapy in the HER2 + subset) revealed elevated Notch signalling for the HER2+, Luminal A and Luminal B groups, while the score was reduced in the TNBC group. In contrast, analysis of the PROMIX cohort after 2 cycles of epirubicin and docetaxel-based chemotherapy showed an increase in signature score in the Luminal B and TNBC subtypes, but not in Luminal A patients. The difference in post-treatment TNBC signature scores comparing the PROMIX and BEAUTY studies is not understood but may be a consequence of which type of chemotherapy was used and for how long. Notably, the PROMIX study utilized epirubicin and docetaxel for 2 cycles prior to biopsy. In contrast, in the BEAUTY study, patients were treated with a 20-week neoadjuvant regimen of paclitaxel followed by doxorubicin and cyclophosphamide, and the second biopsy sample obtained at surgery (only in those with residual disease). It will be of interest to explore in additional TNBC patient cohorts with pre- and post-treatment transcriptomic data whether increased or decreased signature scores from the 20-gene signature are observed. An increase in signature score following treatment would be in line with the previous reports, which have suggested that Notch signalling may be re-activated in response to endocrine therapies for ER^+^ breast cancer [28, 108] and to trastuzumab treatment for HER2^+^ breast cancer [109, 110]; for review see [6]. The finding that a high Notch signature score showed a tendency towards lower survival probability may implicate that elevated Notch signalling is detrimental, which is in agreement with a role for hyperactivated Notch signalling as a driver of breast cancer progression and therapy resistance. It is also of note that the 20-gene signature showed some potential with regard to distinguishing between patients with potentially Notch activating mutations versus patients with no overt Notch pathway mutations in the TCGA patient cohort.

While we believe our data suggest that our Notch signature will prove useful in breast cancer research, it is important to note that the study also has some limitations. For example, we have not had access to, and consequently not analysed, transcriptomic data from completed or ongoing clinical studies with different Notch inhibitors (GSI or antibodies), and analysis of such data will be key to further test the validity of the signature. Furthermore, in the collection of cell line used to train and validate the 20-gene signature, some of the cell lines (HCC1187 and HCC1959) carry mutations in the Notch pathway which constitutively increase Notch signalling, which may obscure the effects of experimental Notch inhibition by GSI. Finally, the notion that the signature score behaved differently in the two TNBC cohorts which had received different therapy regiments suggest that the therapy effects on Notch signalling may be complex and treatment regimen-dependent. Thus, caution should be exerted when using signature scores to read out secondary effects on Notch signalling from various conventional treatment regiments and to rush to the use of transcriptional signatures in the clinic. With this said, we, however, still believe the 20-gene signature holds promise when it comes to gaining insights into the level of Notch signalling in various breast cancer subtypes and to determine the outcome of future Notch-targeting therapies for breast cancer as well as to stratify patients for such therapies.

### Supplementary Information

**Additional file 1. Fig. S1**. A schematic pipeline of data processing for the 20-gene transcriptomic Notch signature. The code for the pipeline is provided at GitHub at the link: https://github.com/KarolinskaMerck/NotchBRCASignature. **Figure S2. A**. Expression of the four NOTCH receptors (NOTCH1-4), the androgen receptor (AR), ERBB2 (HER2), ERBB3 (HER3), ESR1 and pGR1 in the six basal-like cell lines. **B**. Expression of HES1 and NRARP in the six basal-like cell lines under “Notch on” and “Notch off” conditions. **C**. Principal component analysis (P1-20) of the transcriptomic data from the six cell lines under “Notch on”, “Notch off” or “ground state”, as indicated for 8-h (upper) and 72-h (lower) treatment. (*p < 0.05, **p < 0.01, ***p < 0.001 and ****p < 0.0001). Data for PC6 versus PC7 and PC7 versus PC10 PCA combinations are also shown. **D**. Euclidian distances between the Notch-on, ground-state and Notch-off conditions for each of the six cell lines and biological replicates. **Figure S3. A**. Analysis of left skewed P value distribution of the 14 candidate signatures from Fig. 1B. The coherence scores for each signature length in the TCGA-BRCA patient cohort for the candidate signatures are also presented. **B**. Selection of optimal signatures based on both the largest coherence score as well as smallest empirical P value with the smallest gene set size in the range between 10 and 30 genes. **C**. Euclidian distances between the Notch-on, ground-state and Notch-off conditions for each of the six cell lines. **D**. Comparison of the transcriptomes from the 19 cell lines in ground-state conditions to previously established transcriptomes from the same cell lines. **E**. Expression of at least one of the four Notch receptor genes in the 19 cell lines. F. The performance of the candidate signatures to classify Notch activation status in the 19-cell line dataset was evaluated by ROC curves and their AUC (+/− 95% Confidence Interval). **G**. ROC analysis of the 19 breast cancer cell line dataset for signatures #5, #9 and #11; AUC with 95% confidence interval. **Figure S4. A**. Reactome graph-network analysis of the genes in the 20-gene signature to Notch-related pathway terms. **B**. String.db interaction enrichment analysis for the 20-gene signature to Notch-related pathway terms. **Figure S5: A**. Calculation of mean overlap between signatures and assessment of mean similarity using Jaccard coefficient. **B**. Overlap dendrogram of the signatures from A. **C**. Jaccard dendrogram of the signatures from A. **D**. Mean overlap coefficient of the signatures from **A**. **E**. Mean Jaccard similarities from the signatures from A. **F**. Mean centrality score from the signatures from A. **Figure S6: A**. ROC analysis of published Notch signatures analysed in the six cell line datasets. **B**. ROC analysis curves corresponding to the data in A. **C**. GSEA analysis of published Notch signatures analysed in the 19-cell line dataset for Notch on vs off for combined 8+72-h, 8-h and 72-h timepoints, as indicated. **D**. Corresponding ROC analysis for the published Notch signatures corresponding to data in C. **E**. ROC analysis curves for the published Notch signatures analysed in the 19-cell line dataset. **Figure S7**. **A**. Calculation of mean signature scores for the 20-gene signature and previously published signatures in the TCGA, METABRIC and Oslo2 patient cohorts, as indicated. **B**. Inferred immune and stromal content in the TCGA and Oslo2 datasets for the 20-gene signature and previously published Notch signatures, as indicated. **Figure S8: A**. Correlation of the Notch core signature (encompassing the Notch 1-4 receptors and the Jag1,2 and Dll13,4 ligands) to the 20-gene signature and previously published Notch signatures, as indicated. The correlation scores were calculated for six cell line (left) and the 19 cell line (right) datasets. **B**. Correlation of the Notch core signature to previously published Notch signatures for the TCGA, METABRIC and Oslo2 patient datasets, as indicated. **C**. ROC analysis for two binary comparisons of 57 published signatures indicating Notch alterations in the TCGA dataset (“Notch activation alteration” vs “Notch wild type” and “Notch inactivation alteration" vs “Notch wild type”) **D**. Signature score changes for the two binary comparisons in C in 16 previously published signatures, as indicated. **Figure S9**. Calculation of signature scores for the 20-gene signature and previously published signatures in the TCGA, METABRIC and Oslo2 patient cohorts, with basal, HER2, LumA, LumB and normal subtypes, as indicated. **Figure S10**. **A**. Comparison of signature scores (ssGSEA) for three breast cancer subtypes (21 Luminal A, 27 Luminal B and 21 TNBC patients) at the onset (orange) and after two rounds of epirubicin and docetaxel treatment (blue) for previously published Notch signatures, as indicated. **B**. Disease-free survival analysis (survival probability) of patients (*n* = 122 (28 TNBC; 42 LumA and 42 LumB)) at baseline according to signature scores from previously published Notch signatures, as indicated (high (red) and low (blue) groups) at baseline. **C**. Signature score changes in patients with and without lymph node metastasis from previously published signatures and the Notch core signature. **D**. Heatmap comparing the GSVA signature scores from non-responders and responders from the BEAUTY cohort for the 20-gene signature and previously published signatures, as indicated. **E**. Comparison GSVA scores in BEAUTY TNBC responder and non-responder patient cohorts for the 20-gene signature, the Notch core signature and previously published signatures, as indicated.

**Additional file 2. Table S1**: List of Primers used in qRT-PCR.

**Additional file 3. Table S2**: List of all DEGs (up- and downregulated) from the 8- and 72-h timepoints for all breast cancer cell line datasets.

**Additional file 4. Table S3**: The full list of coherence scores of 14 experimental conditions.

**Additional file 5. Table S4: A**. The full list of gene set enrichment scores of 14 experimental conditions (corresponding to Fig. S3E).

**Additional file 6. Table S4: B**. AUC data corresponding to Fig. S3F.

**Additional file 7. Table S5**: List of CSL binding sites GO annotation and KEGG pathways analysis of the 20-gene signature.

**Additional file 8. Table S6: A**. List of all identified Notch signatures used and the corresponding signature genes.

**Additional file 9. Table S6: B**. Similarity scores for previously published Notch signatures, as indicated.

**Additional file 10. Table S6: C**. Centrality scores for previously published Notch signatures, as indicated and distance measurements for individual genes of each signature.

**Additional file 11. Table S7**: A list of the signatures significantly enriched by GSEA (p.adj < 0.05) in the six-cell line training cohort and the larger 19-cell line cohort.

**Additional file 12. Table S8**: List of coherence scores of published signatures in the various patient cohorts (TCGA-BRCA, METABRIC and Oslo2).

**Additional file 13. Table S9: A**. Signature and coherence scores of the 20-gene signature and previously published signatures in all PAM50 subtypes in the various patient cohorts (TCGA-BRCA, METABRIC and Oslo2).

**Additional file 14. Table S9: B**. Signature scores of the 20-gene signature and previously published signatures in individual samples in the six- and 19-cell line panel and in individual samples of the various patient cohorts (TCGA-BRCA, METABRIC and Oslo2) as well as patient characteristics.

**Additional file 15. Table S10**: Full list of analysis of tumour purity, stromal and immune infiltration for the different Notch signatures.

**Additional file 16. Table S11: A**. Correlation of signature score of the Notch core signature to published signatures in the cell line and TCGA, METABRIX and Oslo2 patient datasets.

**Additional file 17. Table S11: B**. cBioportal annotations of Notch mutations in the TCGA cohort, ROC analysis and pair-wise correlation results of published signatures in relation to Notch alteration state.

**Additional file 18. Table S12**: Ground-state signature score changes of the 20-gene signature and published signatures in the 19 breast cancer cell line cohort.

**Additional file 19. Table S13**: Averaged GSVA scores for 16 previously published Notch signatures for the BEAUTY patient cohort of responders and non-responders of the TNBC subtype and averaged ssGSEA scores for the PROMIX patient cohort at baseline and cycle2.

## Data Availability

Transcriptomic data have been uploaded to Gene Expression Omnibus (accession number GSE236749 for the panel of six breast cancer cell lines and accession number GSE235052 for the breast cancer cell line validation dataset). Both datasets have been unified under SuperSeries GSE236750). The code of the bioinformatic analysis is provided at GitHub: https://github.com/KarolinskaMerck/NotchBRCASignature. Data from the PROMIX and BEAUTY studies are available from the authors (TK for PROMIX and MG for BEAUTY) upon reasonable requests.
