# Supplementary material for: Identification of a Notch transcriptomic signature for breast cancer
Source: Breast Cancer Res. 2024 Jan 3;26:4. doi: 10.1186/s13058-023-01757-7 (PMC10765899; doi:10.1186/s13058-023-01757-7)

Fig. S1

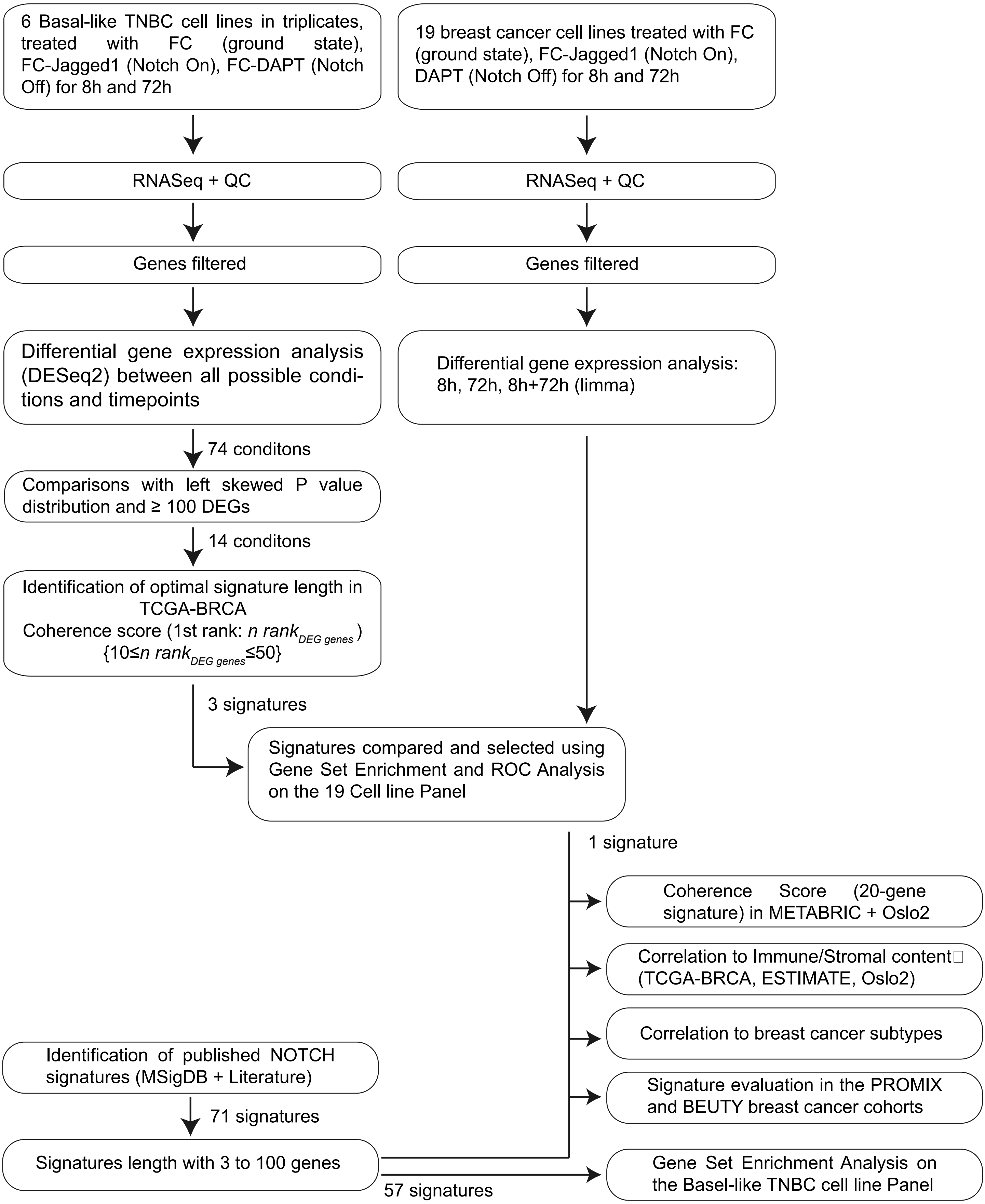
6 Basal-like TNBC cell lines in triplicates, treated with FC (ground state), FC-Jagged1 (Notch On), FC-DAPT (Notch Off) for 8h and 72h
19 breast cancer cell lines treated with FC (ground state), FC-Jagged1 (Notch On), DAPT (Notch Off) for 8h and 72h
RNASeq + QC
RNASeq + QC
Genes filtered
Genes filtered
Differential gene expression analysis (DESeq2) between all possible conditions and timepoints
Differential gene expression analysis: 8h, 72h, 8h+72h (limma)
74 conditons
Comparisons with left skewed P value distribution and ≥ 100 DEGs
14 conditons
Identification of optimal signature length in TCGA-BRCA
Coherence score (1st rank: n rankDEG genes)
{10≤n rankDEG genes≤50}
3 signatures
Signatures compared and selected using Gene Set Enrichment and ROC Analysis on the 19 Cell line Panel
1 signature
Coherence Score (20-gene signature) in METABRIC + Oslo2
Correlation to Immune/Stromal content (TCGA-BRCA, ESTIMATE, Oslo2)
Correlation to breast cancer subtypes
Identification of published NOTCH signatures (MSigDB + Literature)
Signature evaluation in the PROMIX and BEUTY breast cancer cohorts
71 signatures
Signatures length with 3 to 100 genes
57 signatures
Gene Set Enrichment Analysis on the Basel-like TNBC cell line Panel

Fig. S2

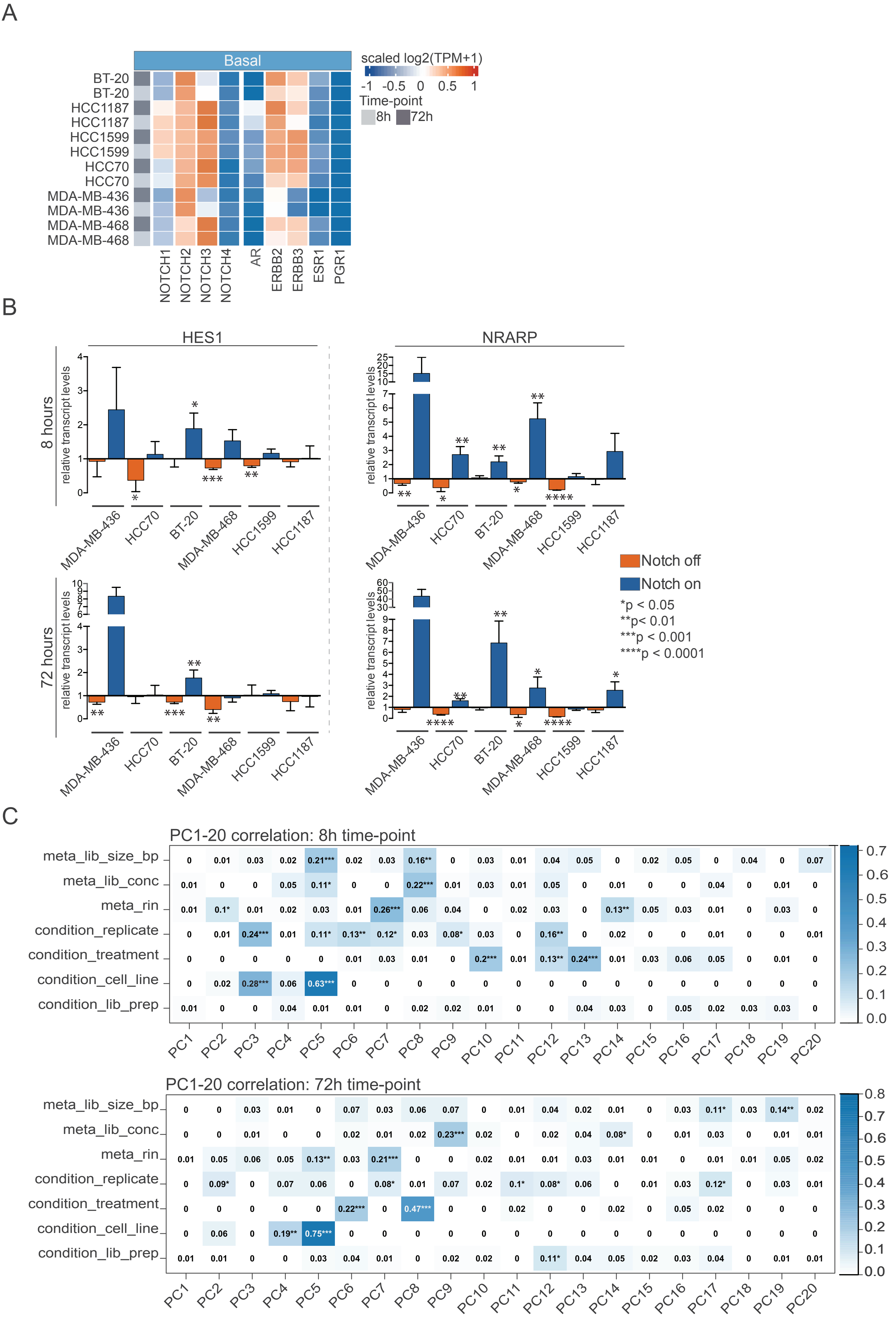
A
Basal
scaled log2(TPM+1)
-1 -0.5 0 0.5 1
Time-point
8h 72h
BT-20
BT-20
HCC1187
HCC1187
HCC1599
HCC1599
HCC70
HCC70
MDA-MB-436
MDA-MB-436
MDA-MB-468
MDA-MB-468
NOTCH1 NOTCH2 NOTCH3 NOTCH4 AR ERBB2 ERBB3 ESR1 PGR1
B
HES1
NRARP
8 hours
72 hours
relative transcript levels
MDA-MB-436 HCC70 BT-20 MDA-MB-468 HCC1599 HCC1187
Notch off
Notch on
*p < 0.05
**p< 0.01
***p < 0.001
****p < 0.0001
C
PC1-20 correlation: 8h time-point
meta_lib_size_bp 0 0.01 0.03 0.02 0.21*** 0.02 0.03 0.16** 0 0.03 0.01 0.04 0.05 0 0.02 0.05 0 0.04 0 0.07
meta_lib_conc 0.01 0 0 0.05 0.11* 0 0 0.22*** 0.01 0.03 0.01 0.05 0 0.01 0 0 0.04 0 0.01 0
meta_rin 0.01 0.1* 0.01 0.02 0.03 0.01 0.26*** 0.06 0.04 0 0.02 0.03 0 0.13** 0.05 0.03 0.01 0 0.03 0
condition_replicate 0 0.01 0.24*** 0.01 0.11* 0.13** 0.12* 0.03 0.08* 0.03 0 0.16** 0 0.02 0 0 0.01 0 0 0.01
condition_treatment 0 0 0 0 0 0.01 0.03 0.01 0 0.2*** 0.01 0.13** 0.24*** 0.01 0.03 0.06 0.05 0 0.01 0
condition_cell_line 0 0.02 0.28*** 0.06 0.63*** 0 0 0 0 0 0 0 0 0 0 0 0 0 0 0
condition_lib_prep 0.01 0 0 0.04 0.01 0.01 0 0.02 0.02 0.01 0 0 0.04 0.03 0 0.05 0.02 0.03 0.03 0
PC1 PC2 PC3 PC4 PC5 PC6 PC7 PC8 PC9 PC10 PC11 PC12 PC13 PC14 PC15 PC16 PC17 PC18 PC19 PC20
PC1-20 correlation: 72h time-point
meta_lib_size_bp 0 0 0.03 0.01 0 0.07 0.03 0.06 0.07 0 0.01 0.04 0.02 0.01 0 0.03 0.11* 0.03 0.14** 0.02
meta_lib_conc 0 0 0.01 0 0 0.02 0.01 0.02 0.23*** 0.02 0 0.02 0.04 0.08* 0 0.01 0.03 0 0.01 0.01
meta_rin 0.01 0.05 0.06 0.05 0.13** 0.03 0.21*** 0 0 0.02 0.01 0.01 0.03 0.01 0.01 0 0.01 0.01 0.05 0.02
condition_replicate 0 0.09* 0 0.07 0.06 0 0.08* 0.01 0.07 0.02 0.1* 0.08* 0.06 0 0.01 0.03 0.12* 0 0.03 0.01
condition_treatment 0 0 0 0 0 0.22*** 0 0.47*** 0 0 0.02 0 0 0.02 0 0.05 0.02 0 0 0
condition_cell_line 0 0.06 0 0.19** 0.75*** 0 0 0 0 0 0 0 0 0 0 0 0 0 0 0
condition_lib_prep 0.01 0.01 0 0 0.03 0.04 0.01 0 0.02 0.02 0 0.11* 0.04 0.05 0.02 0.03 0.04 0 0.01 0.01
PC1 PC2 PC3 PC4 PC5 PC6 PC7 PC8 PC9 PC10 PC11 PC12 PC13 PC14 PC15 PC16 PC17 PC18 PC19 PC20

Fig. S2C continued

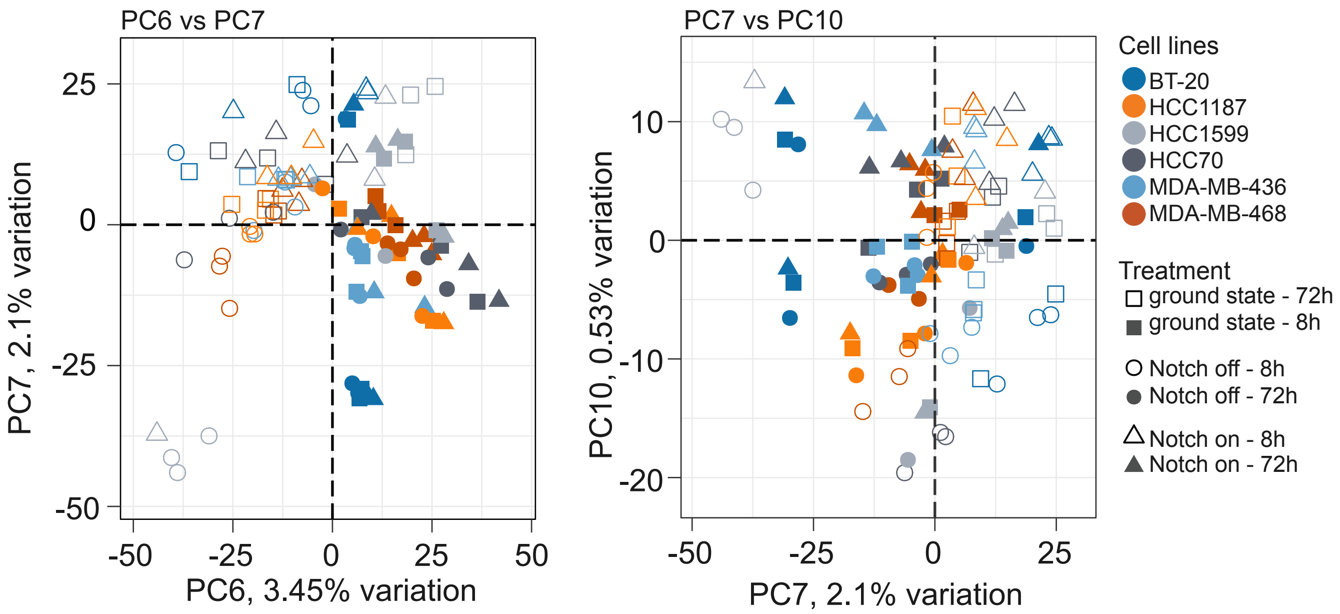

Fig. S2 continued

D

Euclidean Distance between the samples with complete clustering of the six cell line dataset

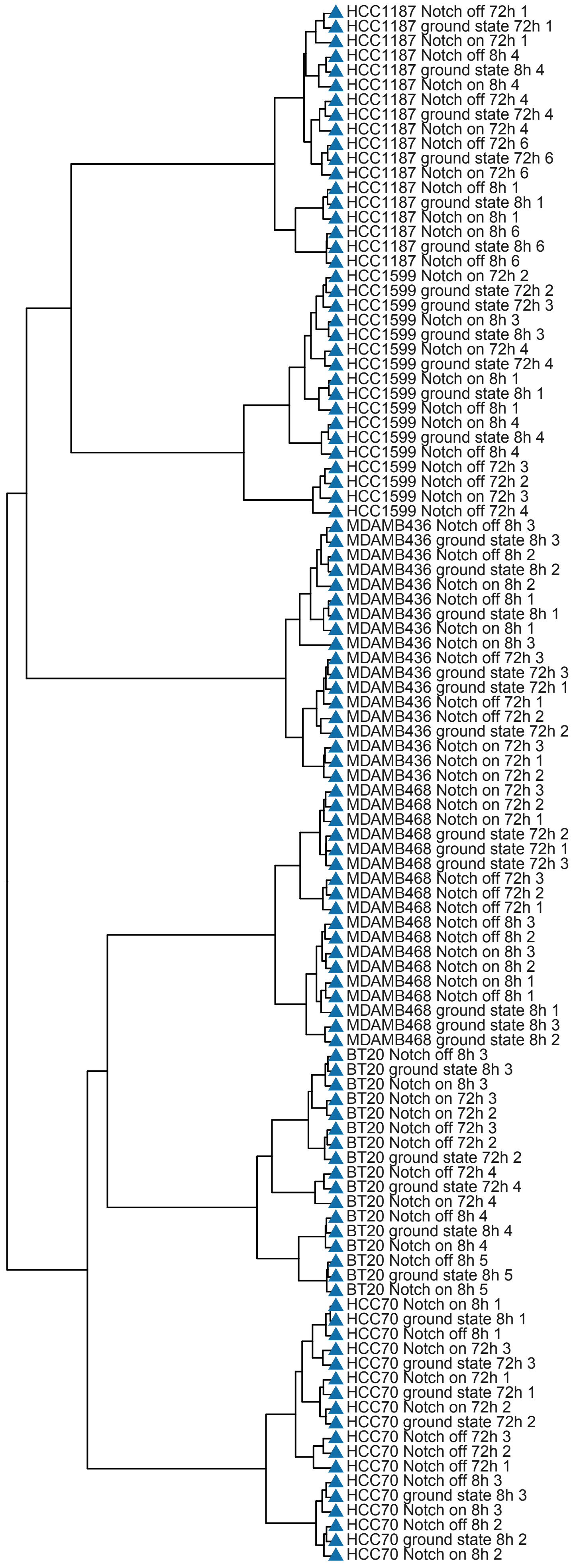

Fig. S3

A

## Tuning the number of signature genes

Signature 1:
Notch On vs. Notch Off 8h up

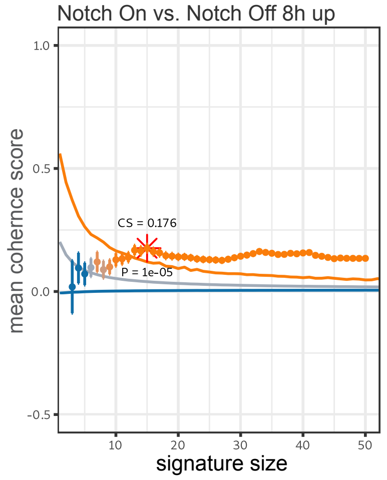


signature genes

NRARP, RHOV, FLT1, ID3 , PLAT, SEMA5B, KCNN3 , ADAMTS15, RGS4 , JAG1, PRELP, HEY1, HEYL,PDZD2, FAM43A

Signature 2:
Ground State vs. Notch Off 72h up

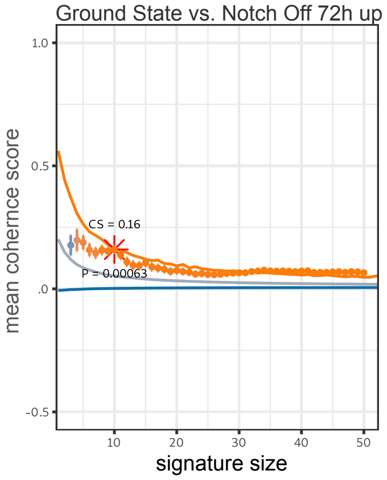


signature genes

PIP, BCAS1, CDH5, BPIFB1, MGAM, DHRS9, PIGR, VSIG2, LPAR1, COL17A1

Signature 3:
Notch On vs. Notch Off 72h up

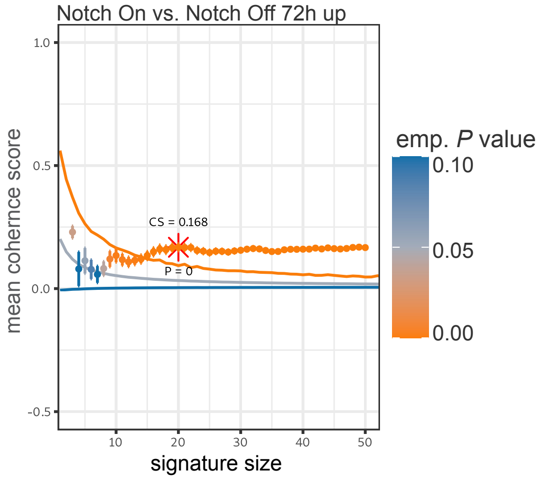


signature genes

SEMA5B, ASB2, PRELP, SERPINA3, PLA2G2A, NRARP, PLAT, FAT2, KRT5, LRRC32, TMC3, KLK14, FOXS1, COL5A3, SNAI2, KRT14, OLFML1, KRT6A, HEYL, DNASE1L3

Signature 4:
Ground State vs. Notch Off 8h + 72h up

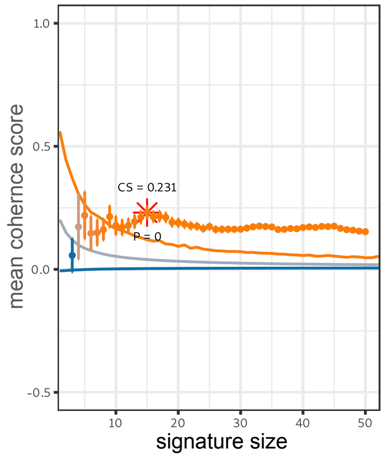


signature genes

PIP, BPIFB1, BCAS1, CDH5, DHRS9, ENTPD8, TMPRSS4, IGFBP5, MGAM, CACNA1I, PSCA , TSHZ2, CPEB1, ACHE, PTPRH, FUT2, ADRA2B, MUC3A, CYP4F12, SVEP1, DLEC1, LDHD, TXLNB, CD68, PLA2G4F, COL21A1

Signature 5:
Notch On vs. Notch Off 8h + 72h up

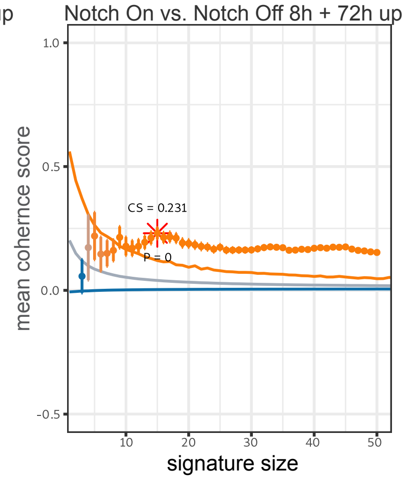


signature genes

NRARP, POSTN, SEMA5B, OLFML1, SLC1A7, TMC3, PLAT, PRELP,
LRRC32, RHOV, FAT2, KRT14, COL5A3, HEYL, SNAI2

Signature 6:
Notch On vs. Notch Off 8h down

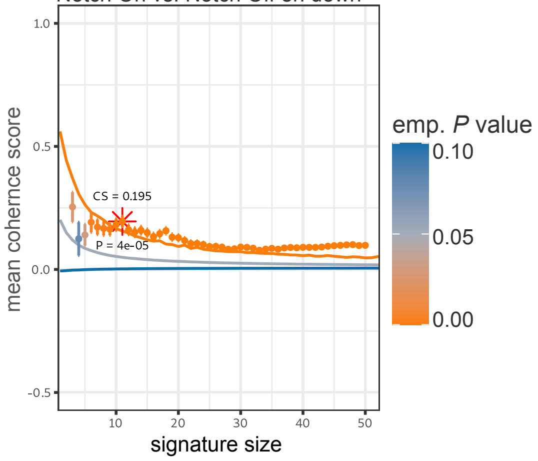


signature genes

RIT1, ARHGEF5, PNPLA8, MEIS2, CTDSP2, SECISBP2L, DISP1, RALGPS1, BCL10, ATP8B1, BCOR

Signature 7:
Ground State vs. Notch Off 72h down

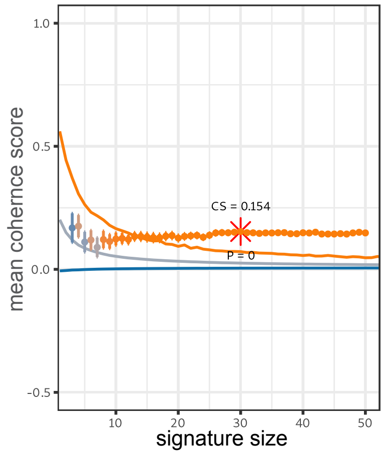


signature genes

CDK5RAP1, SMU1, PLAGL2, ABCB7, RNF220, PCNP, MRPL20, PSMB2, EIF4ENIF1, C18orf21, DDB1, GABPB1, PPP2R5D, NUP98, PPP4R2, CCNT1, PEX26, ABCF1, HNRNPUL1, NDUFA10, POLR3A , YWHAE, HADHA, MRFAP1, POLR2B, KHDRBS1, SF3B2, CNOT7, CELF1, RBBP5

Signature 8:
Ground State vs. Notch Off 8h + 72h down

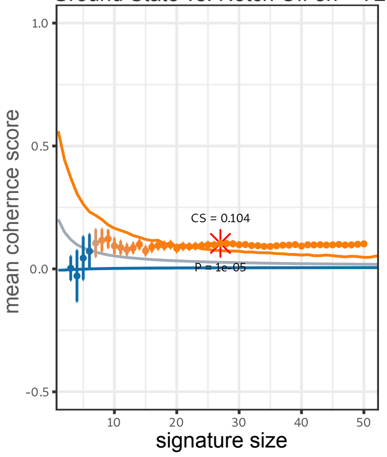


signature genes

CLP1, DRG1, EIF3K, CIAPIN1, SF3B3, SSB, PCID2, USP7, CDC123, PRCC, EIF3I, LCMT1, ERI3, TARDBP, RWDD1, PSMA5, SSBP1

Signature 9:
Notch On vs. Notch Off 8h + 72h down

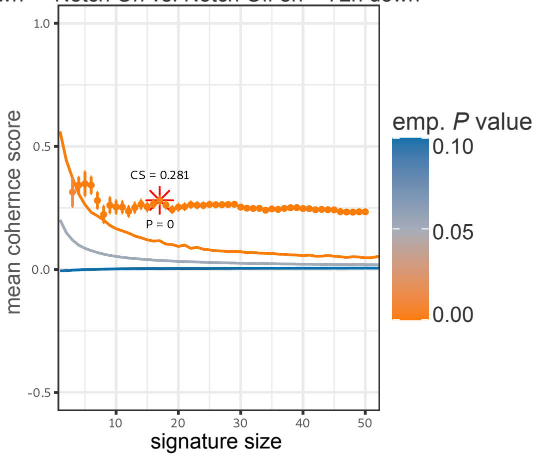


signature genes

ARL8A, ZBTB34, P4HB, ESYT1, USP6NL, SMURF1, ATP6V1B2, FBXO11, SHOC2, FKBP15, ABL1, ZNF322, TRAPPC8, AKAP10

Fig. S3A continued

Signature 10:
Notch On vs. Notch Off 8h + 72h down

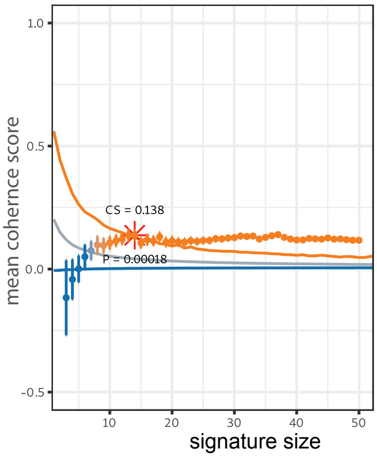


signature genes

ARL8A, ZBTB34, P4HB, ESYT1, USP6NL, SMURF1, ATP6V1B2, FBXO11, SHOC2, FKBP15, ABL1, ZNF322, TRAPPC8, AKAP10

Signature 11: Notch On vs. Notch Off 72h Up" ***and*** "Notch On vs. Notch Off 8h Up"

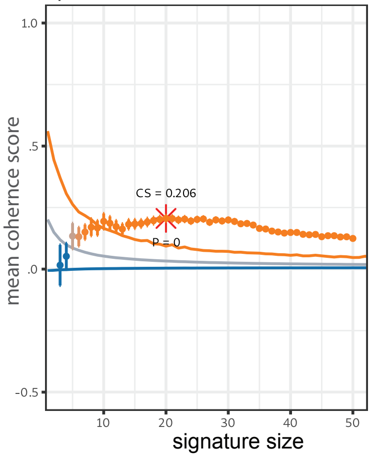


signature genes

SEMA5B, NRARP, PLAT, PRELP, HEYL, FAT2, HEY1, KRT5, NPR3, KRT14, FLT1, RHOV, TNFRSF19, JAG1, MT1X, HEY2, PDGFRB , ZNF469, VSNL1, KIT

Signature 12: Ground State vs. Notch Off 72h Down" ***and*** "Notch On vs. Notch Off 8h Up"

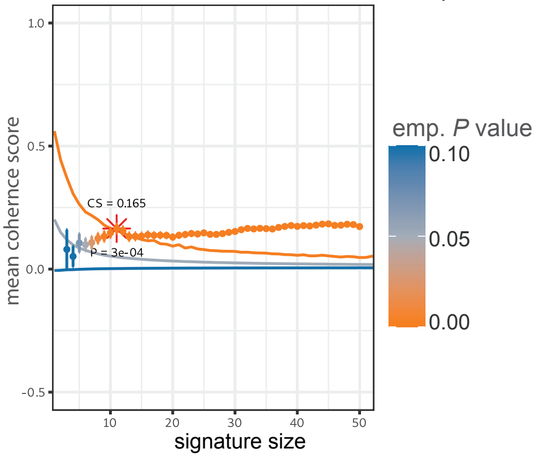


signature genes

C18orf21, DDX24, SRRD, MAP3K4, ZC3H15, ZNF343, WRNIP1, DNAAF2, TRIT1, APEX1 , EIF5B

Signature 13:
Notch On vs. Notch Off 72h Down" ***and*** "Ground State vs. Notch Off 72h Up"

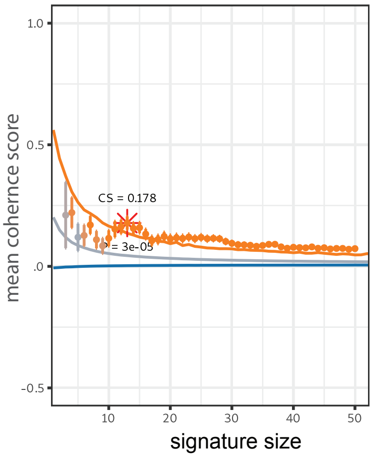


signature genes

NFKBIL1, CHFR, WDR45, SMOX, NAGA, RHBDD2, HS1BP3, PCGF3, IL17RA, ZNHIT1, FAM3A, DEF8, TMEM120A

Signature 14: Ground State vs. Notch Off 72h Down" ***and*** "Notch On vs. Notch Off 72h Up"

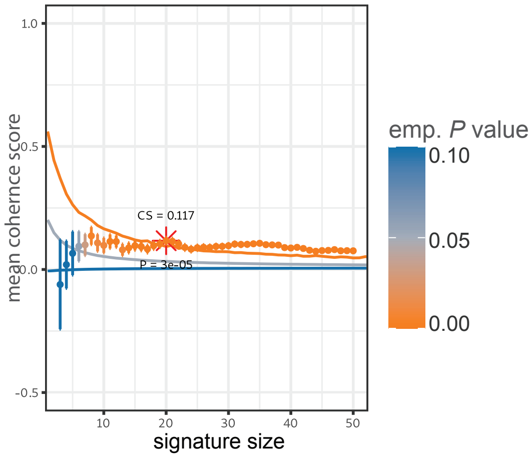


signature genes

ROBO1, PRKX, EPB41L5, AP3M2, PDE7A, RPAP2, ZNF343, GTF3C4, CCNB1IP1, ABCC5, TRDMT1, COX10, METTL17, DSP, LSM11, CNKSR3, METTL3, ZBTB39, SP4, LRP6

B

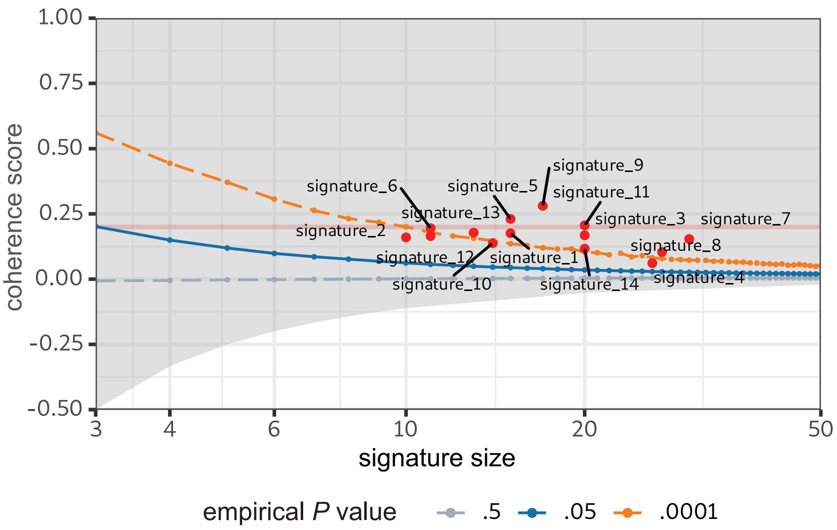

Fig. S3 continued
C

Euclidean Distance between the samples with complete clustering of the 19 cell line dataset

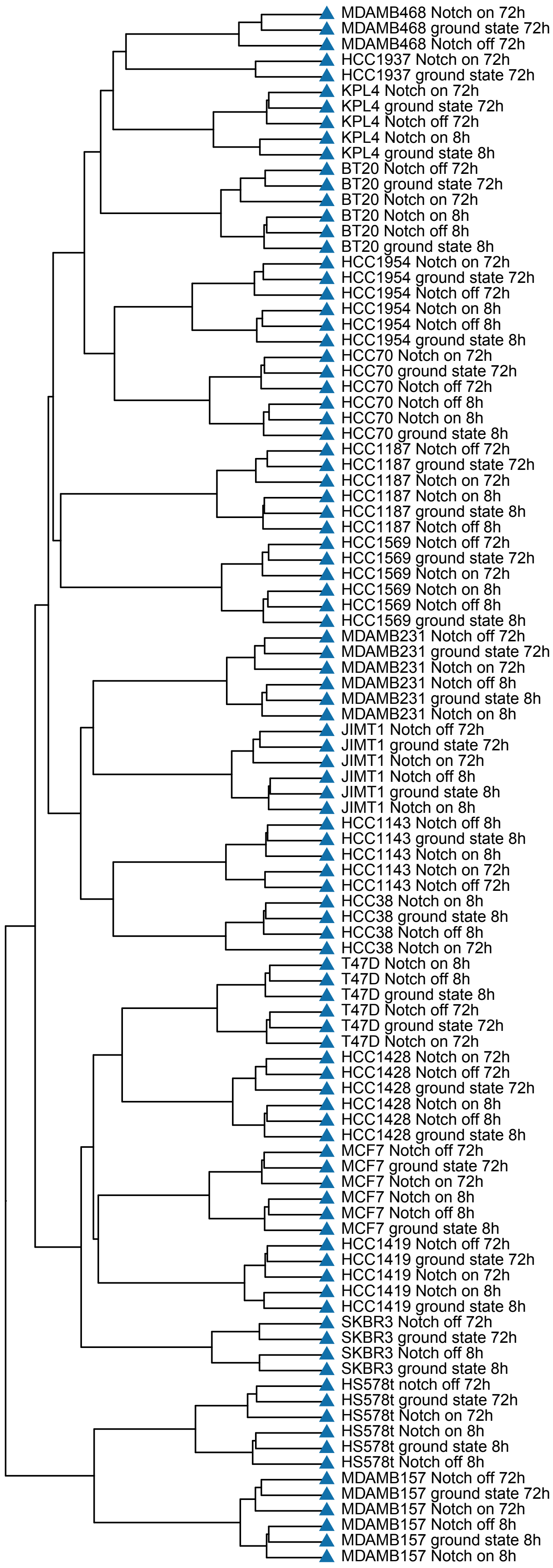

Fig. S3 continued

D

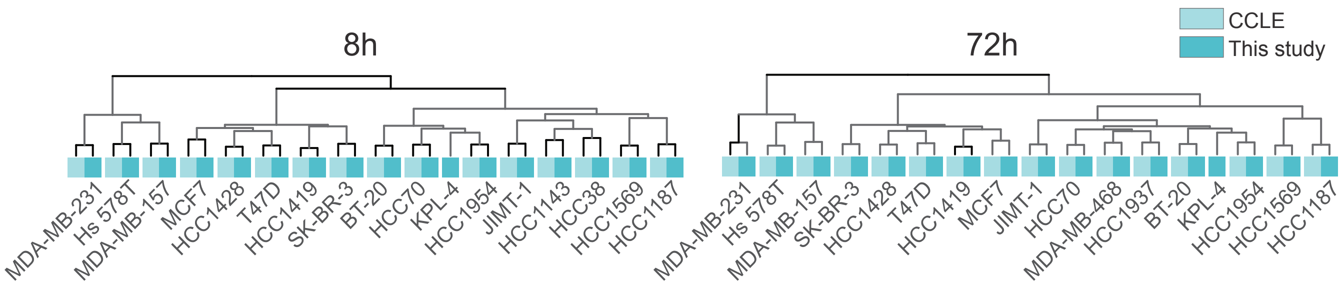


E

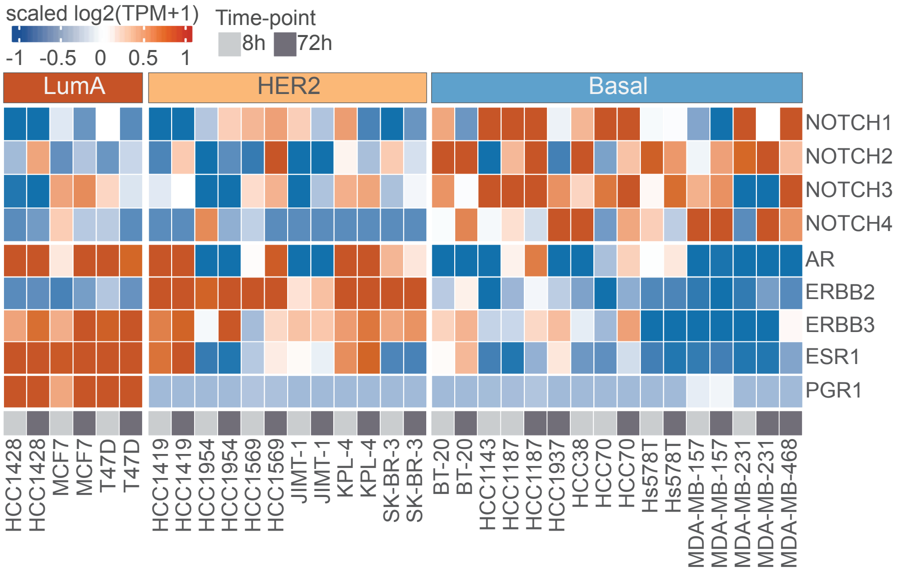

Fig. S3 continued

F

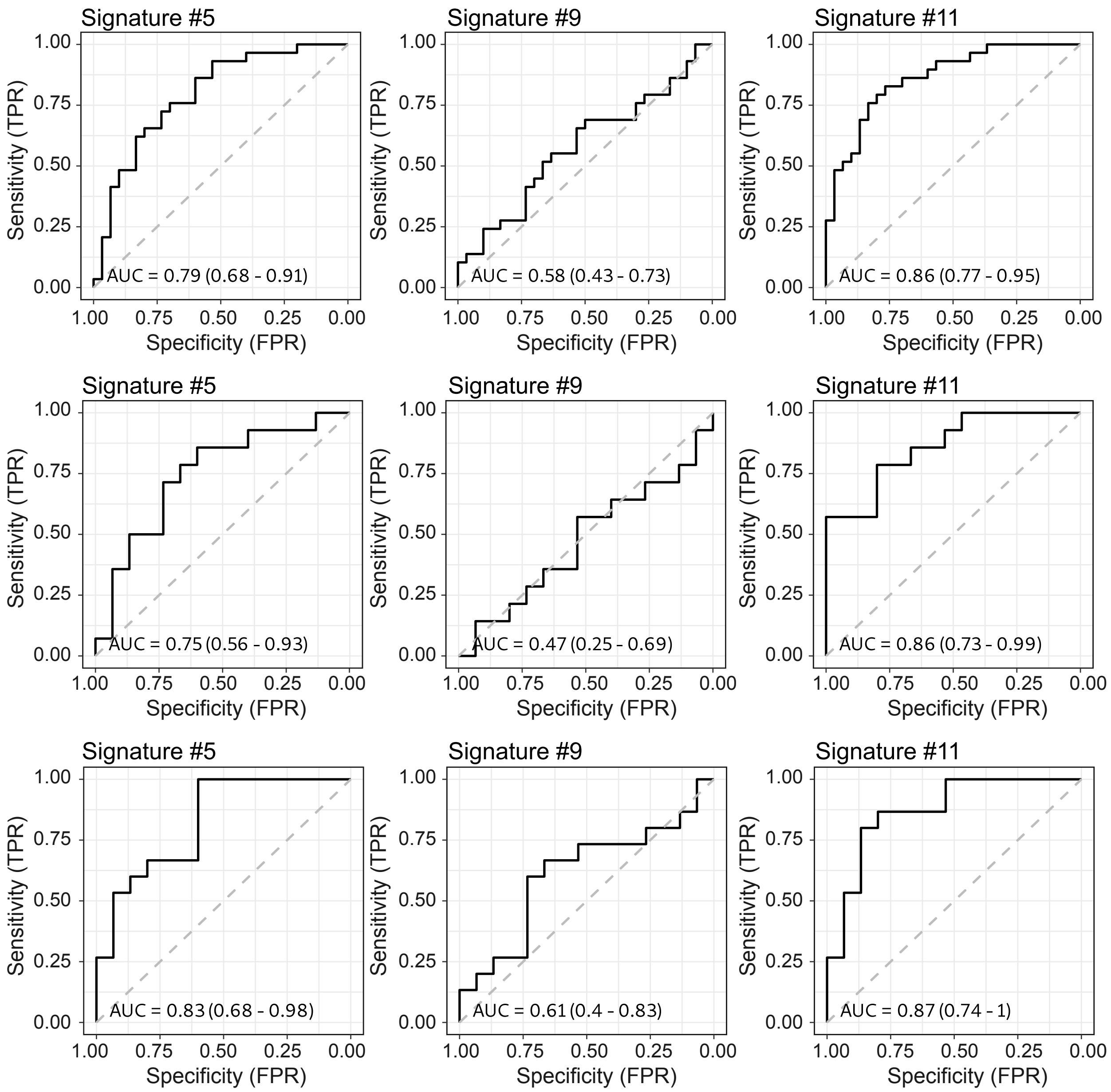


G

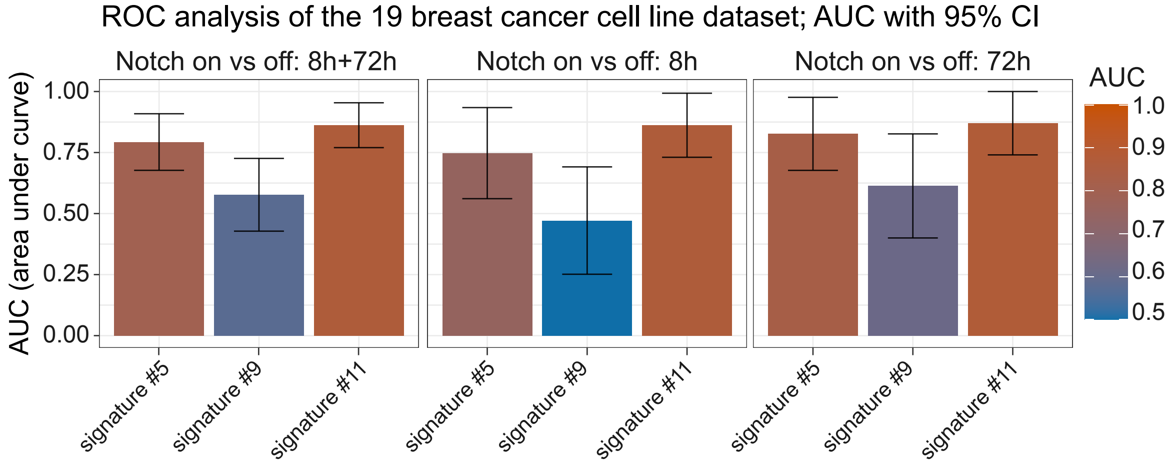

Fig. S4

A

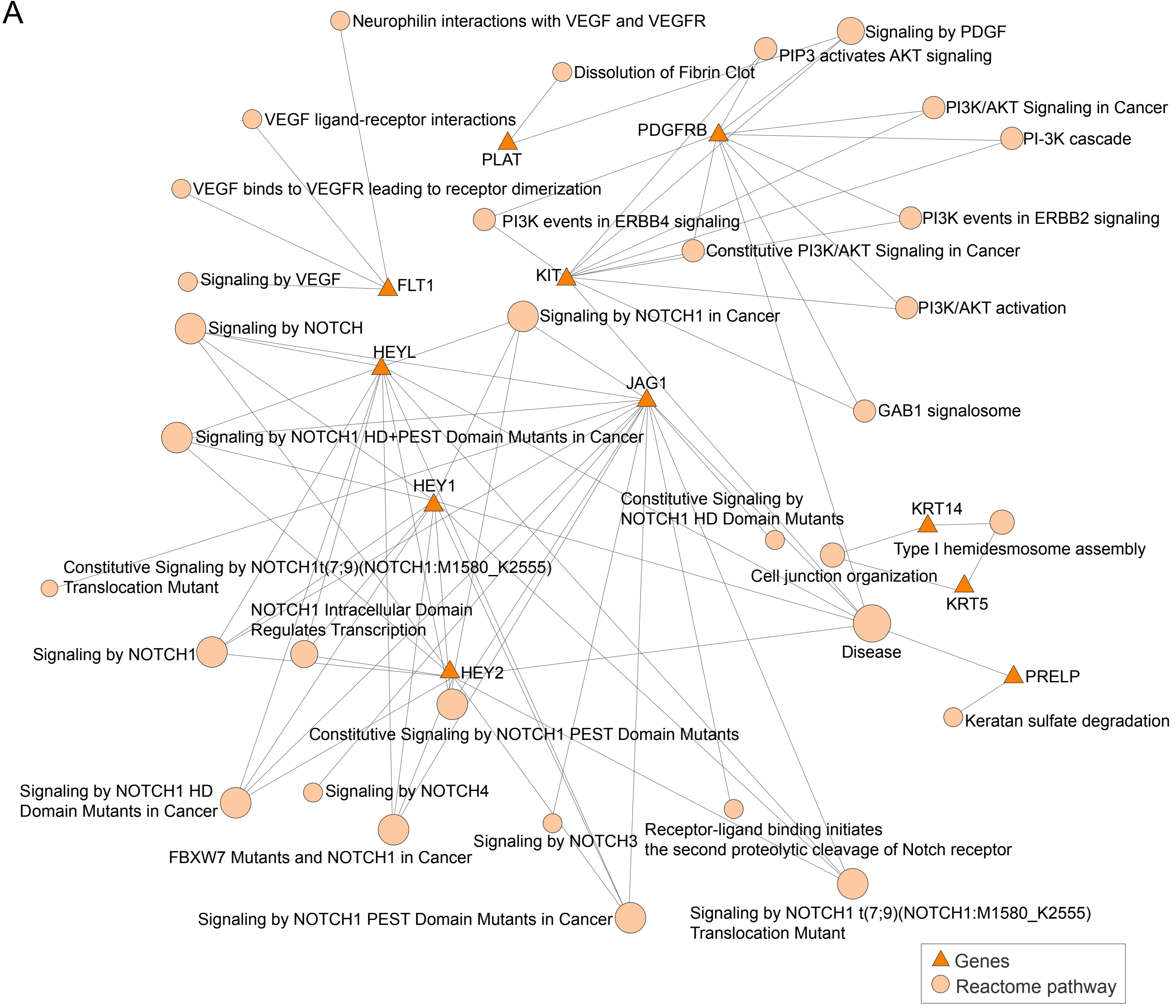

Neurophilin interactions with VEGF and VEGFR
Signaling by PDGF
PIP3 activates AKT signaling
Dissolution of Fibrin Clot
PI3K/AKT Signaling in Cancer
VEGF ligand-receptor interactions
PDGFRB
PI-3K cascade
PLAT
VEGF binds to VEGFR leading to receptor dimerization
PI3K events in ERBB4 signaling
PI3K events in ERBB2 signaling
Constitutive PI3K/AKT Signaling in Cancer
KIT
Signaling by VEGF
FLT1
PI3K/AKT activation
Signaling by NOTCH
Signaling by NOTCH1 in Cancer
HEYL
JAG1
GAB1 signalosome
Signaling by NOTCH1 HD+PEST Domain Mutants in Cancer
HEY1
Constitutive Signaling by
NOTCH1 HD Domain Mutants
KRT14
Type I hemidesmosome assembly
Constitutive Signaling by NOTCH1t(7;9)(NOTCH1:M1580_K2555)
Translocation Mutant
Cell junction organization
KRT5
NOTCH1 Intracellular Domain
Regulates Transcription
Signaling by NOTCH1
Disease
HEY2
PRELP
Keratan sulfate degradation
Constitutive Signaling by NOTCH1 PEST Domain Mutants
Signaling by NOTCH1 HD
Domain Mutants in Cancer
Signaling by NOTCH4
Receptor-ligand binding initiates
the second proteolytic cleavage of Notch receptor
Signaling by NOTCH3
FBXW7 Mutants and NOTCH1 in Cancer
Signaling by NOTCH1 PEST Domain Mutants in Cancer
Signaling by NOTCH1 t(7;9)(NOTCH1:M1580_K2555)
Translocation Mutant
Genes
Reactome pathway


B

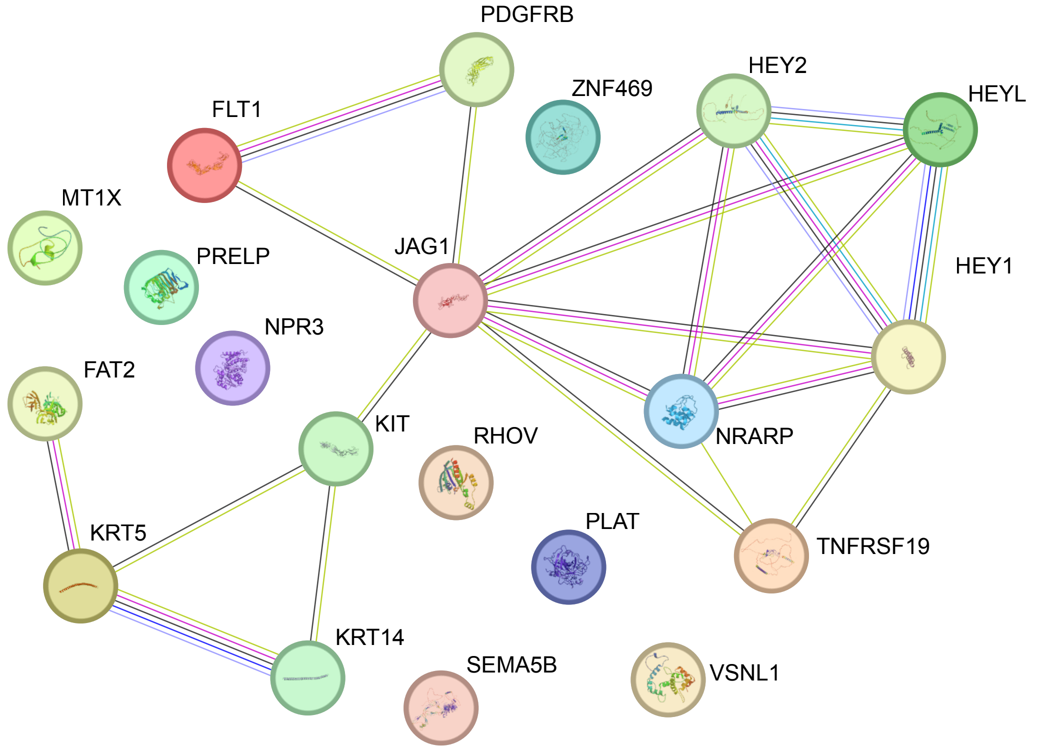

PDGFRB
ZNF469
HEY2
HEYL
FLT1
MT1X
PRELP
JAG1
HEY1
NPR3
FAT2
KIT
RHOV
NRARP
KRT5
PLAT
TNFRSF19
KRT14
SEMA5B
VSNL1

Fig. S5

A

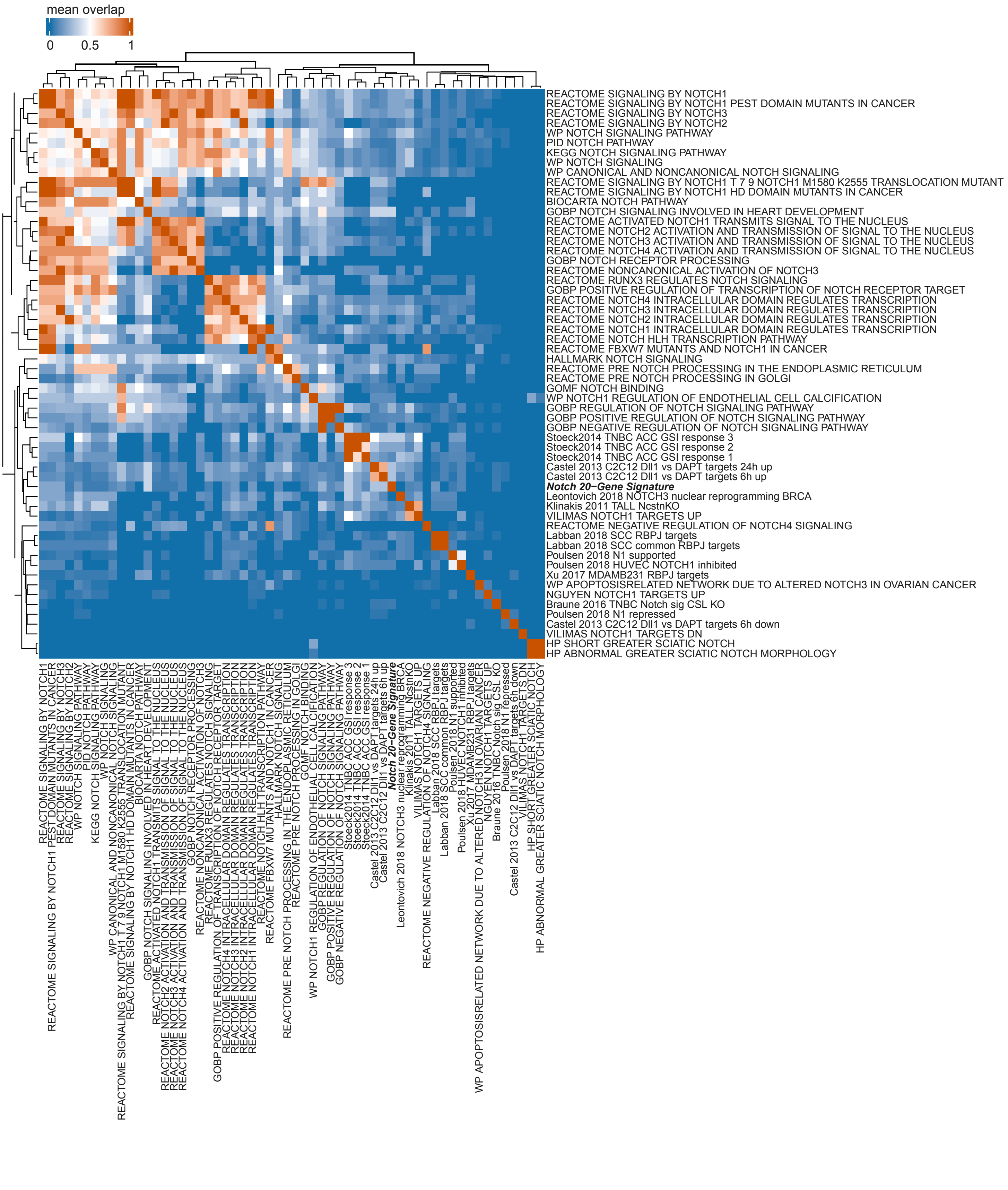
mean overlap
0
0.5
1
REACTOME SIGNALING BY NOTCH1
REACTOME SIGNALING BY NOTCH1 PEST DOMAIN MUTANTS IN CANCER
REACTOME SIGNALING BY NOTCH3
REACTOME SIGNALING BY NOTCH2
WP NOTCH SIGNALING PATHWAY
PID NOTCH PATHWAY
KEGG NOTCH SIGNALING PATHWAY
WP NOTCH SIGNALING
WP CANONICAL AND NONCANONICAL NOTCH SIGNALING
REACTOME SIGNALING BY NOTCH1 T 7 9 NOTCH1 M1580 K2555 TRANSLOCATION MUTANT
REACTOME SIGNALING BY NOTCH1 HD DOMAIN MUTANTS IN CANCER
BIOCARTA NOTCH PATHWAY
GOBP NOTCH SIGNALING INVOLVED IN HEART DEVELOPMENT
REACTOME ACTIVATED NOTCH1 TRANSMITS SIGNAL TO THE NUCLEUS
REACTOME NOTCH2 ACTIVATION AND TRANSMISSION OF SIGNAL TO THE NUCLEUS
REACTOME NOTCH3 ACTIVATION AND TRANSMISSION OF SIGNAL TO THE NUCLEUS
REACTOME NOTCH4 ACTIVATION AND TRANSMISSION OF SIGNAL TO THE NUCLEUS
GOBP NOTCH RECEPTOR PROCESSING
REACTOME NONCANONICAL ACTIVATION OF NOTCH3
REACTOME RUNX3 REGULATES NOTCH SIGNALING
GOBP POSITIVE REGULATION OF TRANSCRIPTION OF NOTCH RECEPTOR TARGET
REACTOME NOTCH4 INTRACELLULAR DOMAIN REGULATES TRANSCRIPTION
REACTOME NOTCH3 INTRACELLULAR DOMAIN REGULATES TRANSCRIPTION
REACTOME NOTCH2 INTRACELLULAR DOMAIN REGULATES TRANSCRIPTION
REACTOME NOTCH1 INTRACELLULAR DOMAIN REGULATES TRANSCRIPTION
REACTOME NOTCH HLH TRANSCRIPTION PATHWAY
REACTOME FBXW7 MUTANTS AND NOTCH1 IN CANCER
HALLMARK NOTCH SIGNALING
REACTOME PRE NOTCH PROCESSING IN THE ENDOPLASMIC RETICULUM
REACTOME PRE NOTCH PROCESSING IN GOLGI
GOMF NOTCH BINDING
WP NOTCH1 REGULATION OF ENDOTHELIAL CELL CALCIFICATION
GOBP REGULATION OF NOTCH SIGNALING PATHWAY
GOBP POSITIVE REGULATION OF NOTCH SIGNALING PATHWAY
GOBP NEGATIVE REGULATION OF NOTCH SIGNALING PATHWAY
Stoeck2014 TNBC ACC GSI response 3
Stoeck2014 TNBC ACC GSI response 2
Stoeck2014 TNBC ACC GSI response 1
Castel 2013 C2C12 Dll1 vs DAPT targets 24h up
Castel 2013 C2C12 Dll1 vs DAPT targets 6h up
Notch 20−Gene Signature
Leontovich 2018 NOTCH3 nuclear reprogramming BRCA
Klinakis 2011 TALL NcstnKO
VILIMAS NOTCH1 TARGETS UP
REACTOME NEGATIVE REGULATION OF NOTCH4 SIGNALING
Labban 2018 SCC RBPJ targets
Labban 2018 SCC common RBPJ targets
Poulsen 2018 N1 supported
Poulsen 2018 HUVEC NOTCH1 inhibited
Xu 2017 MDAMB231 RBPJ targets
WP APOPTOSISRELATED NETWORK DUE TO ALTERED NOTCH3 IN OVARIAN CANCER
NGUYEN NOTCH1 TARGETS UP
Braune 2016 TNBC Notch sig CSL KO
Poulsen 2018 N1 repressed
Castel 2013 C2C12 Dll1 vs DAPT targets 6h down
VILIMAS NOTCH1 TARGETS DN
HP SHORT GREATER SCIATIC NOTCH
HP ABNORMAL GREATER SCIATIC NOTCH MORPHOLOGY

Fig. S5 continued
B

Overlap Dendrogram

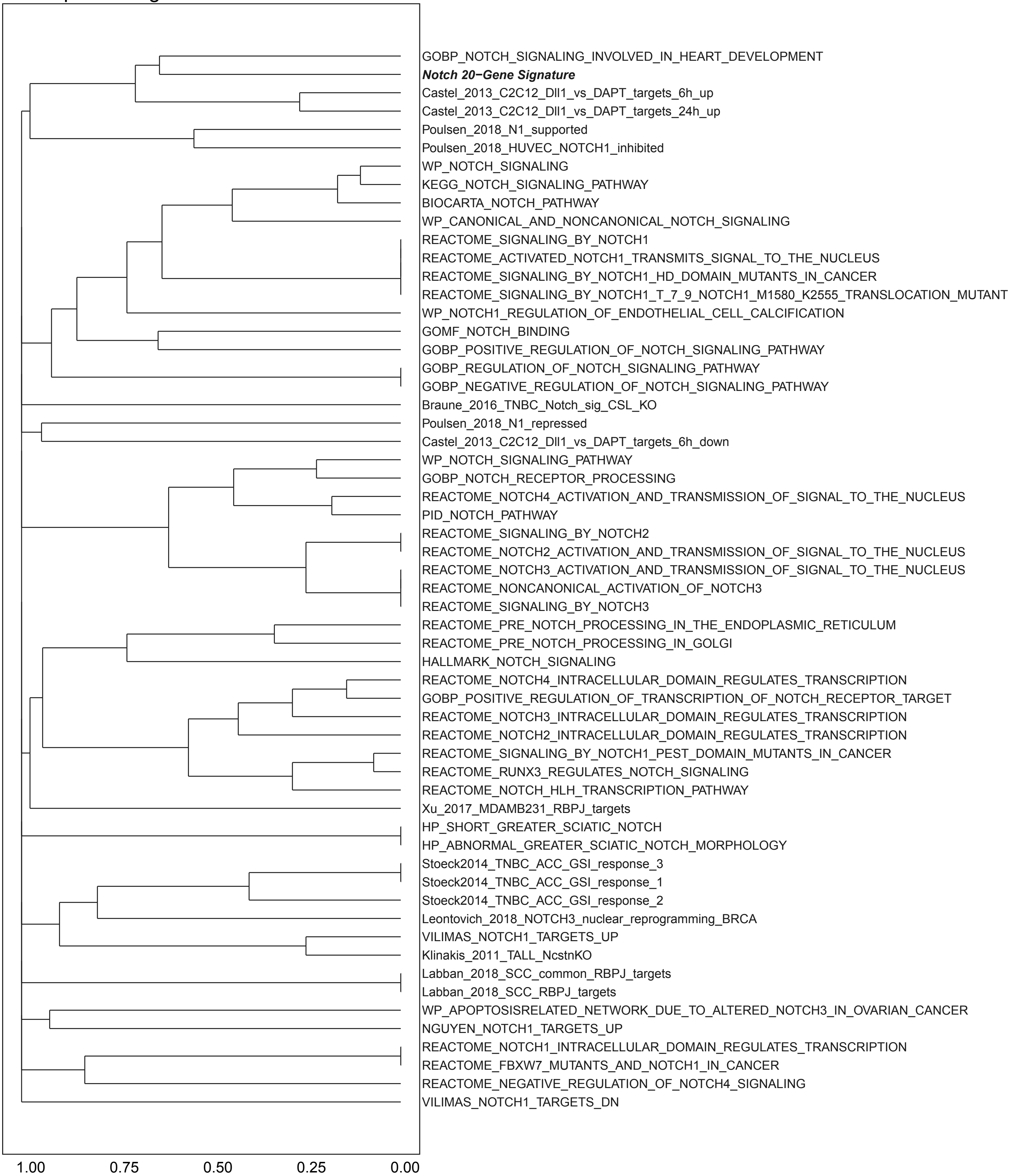

Fig. S5 continued
C

Jaccard Dendrogram

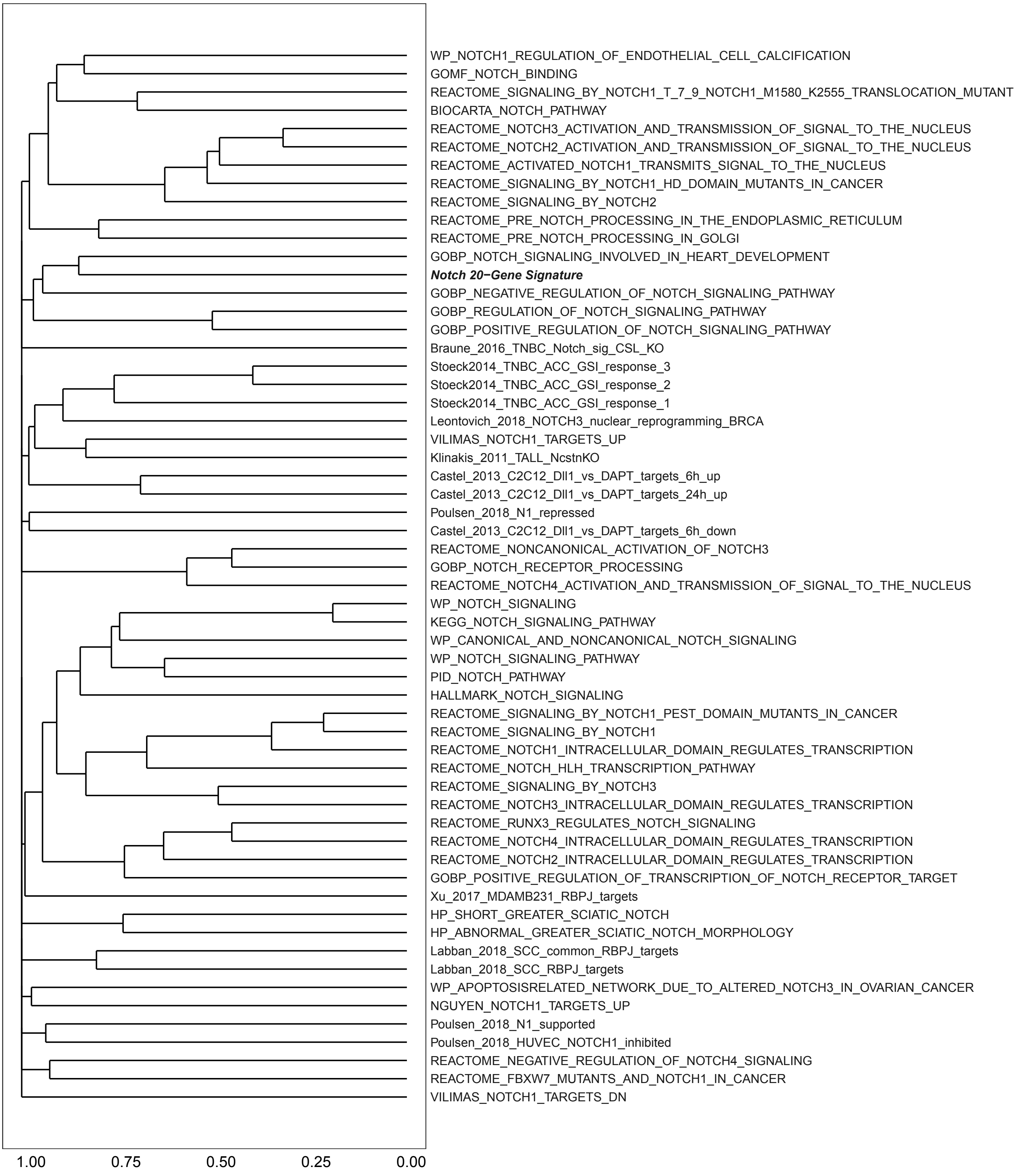

Fig. S5 continued

D

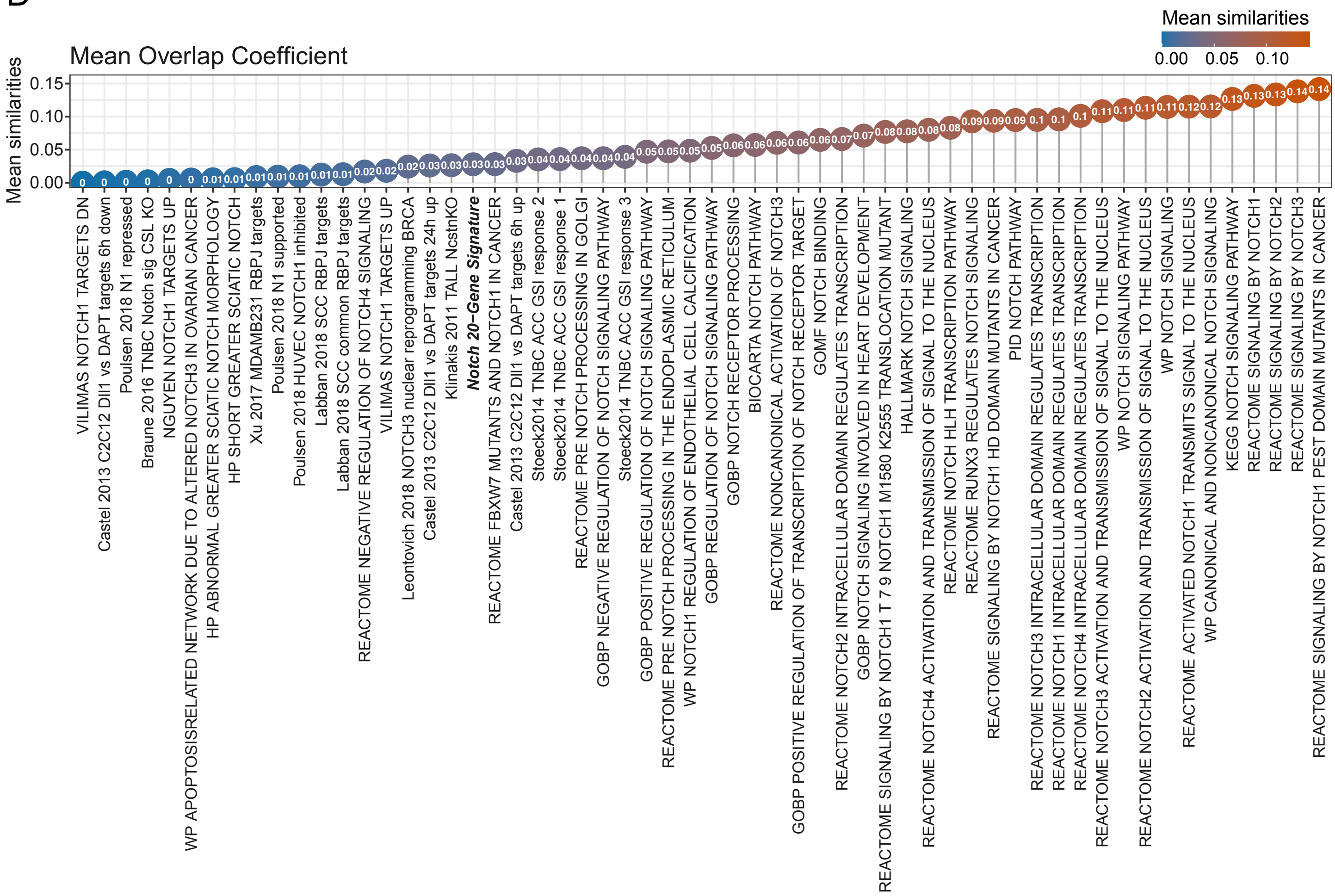


E

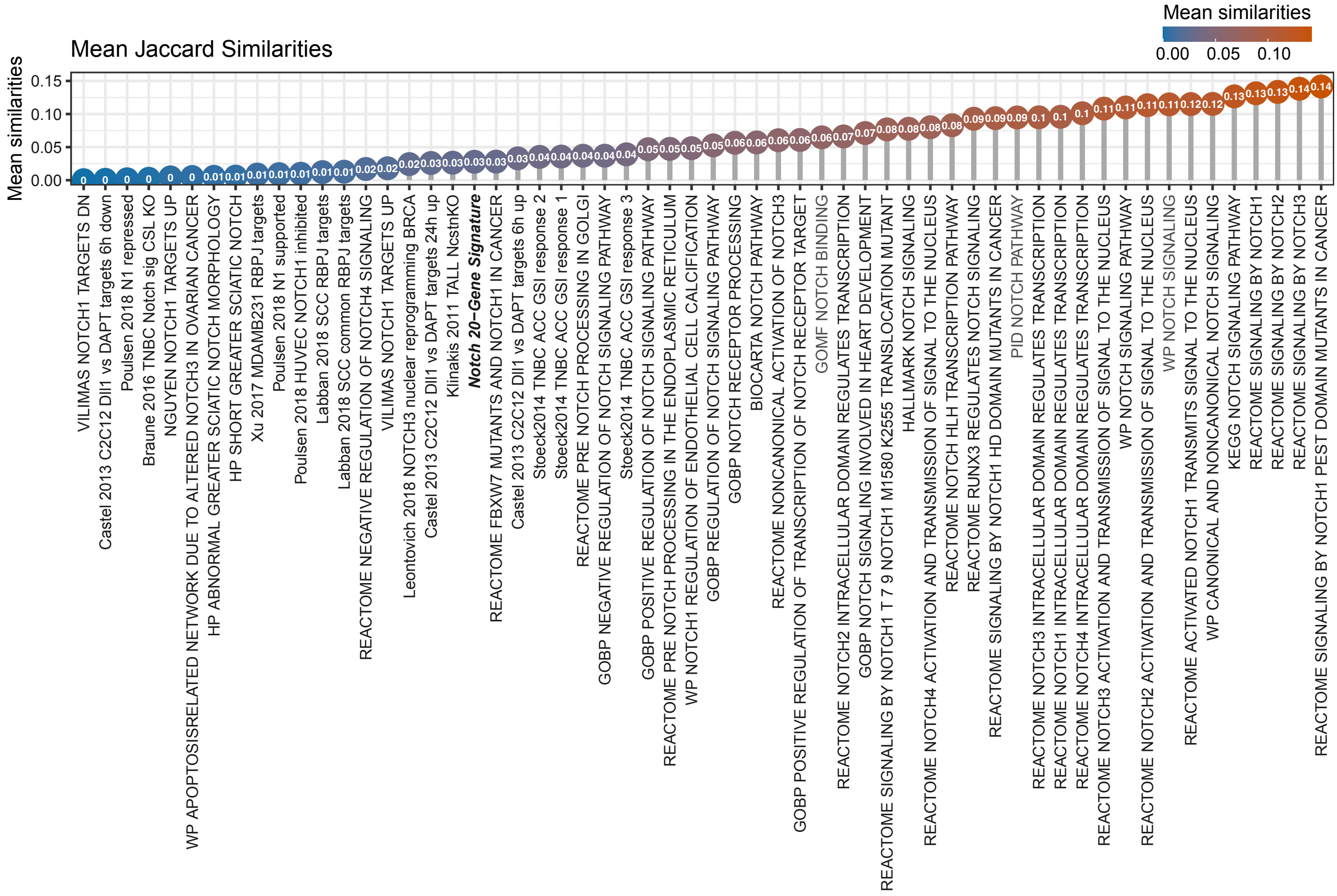

# Fig. S5 continued
F

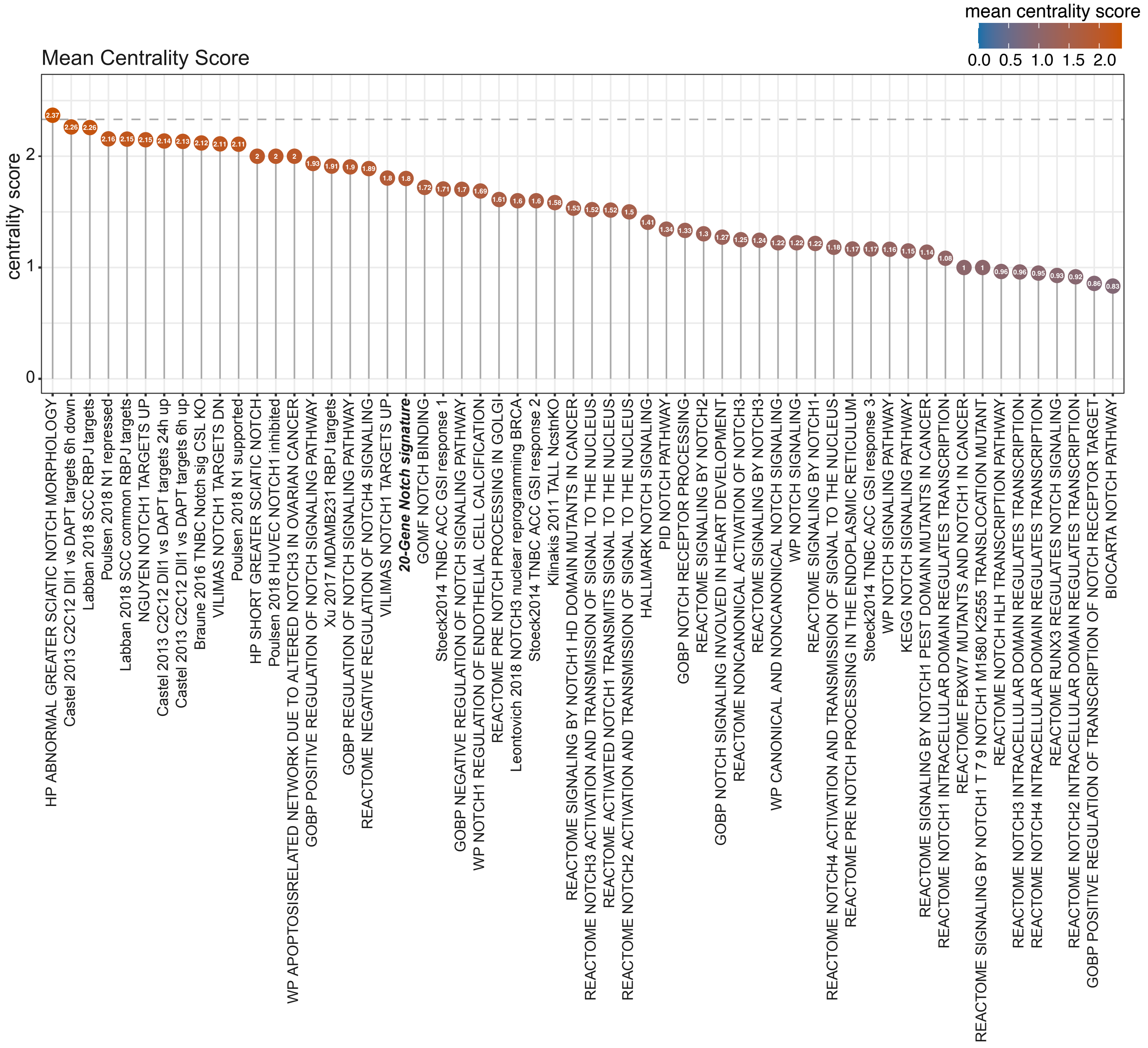

Fig. S6
A

ROC analysis of previously published Notch signatures analysed in the six cell line dataset; AUC with 95% CI

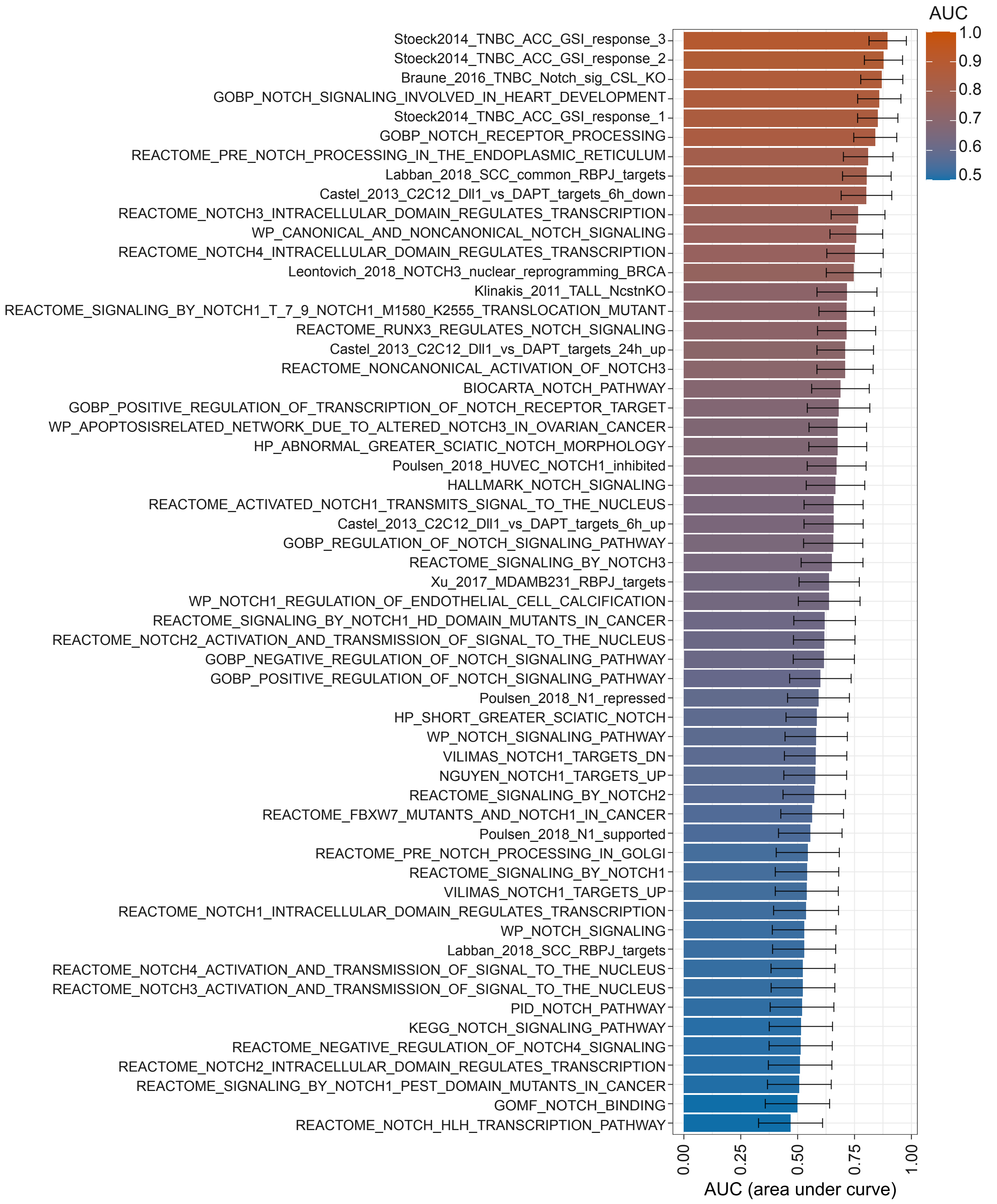

Fig. S6 continued
B

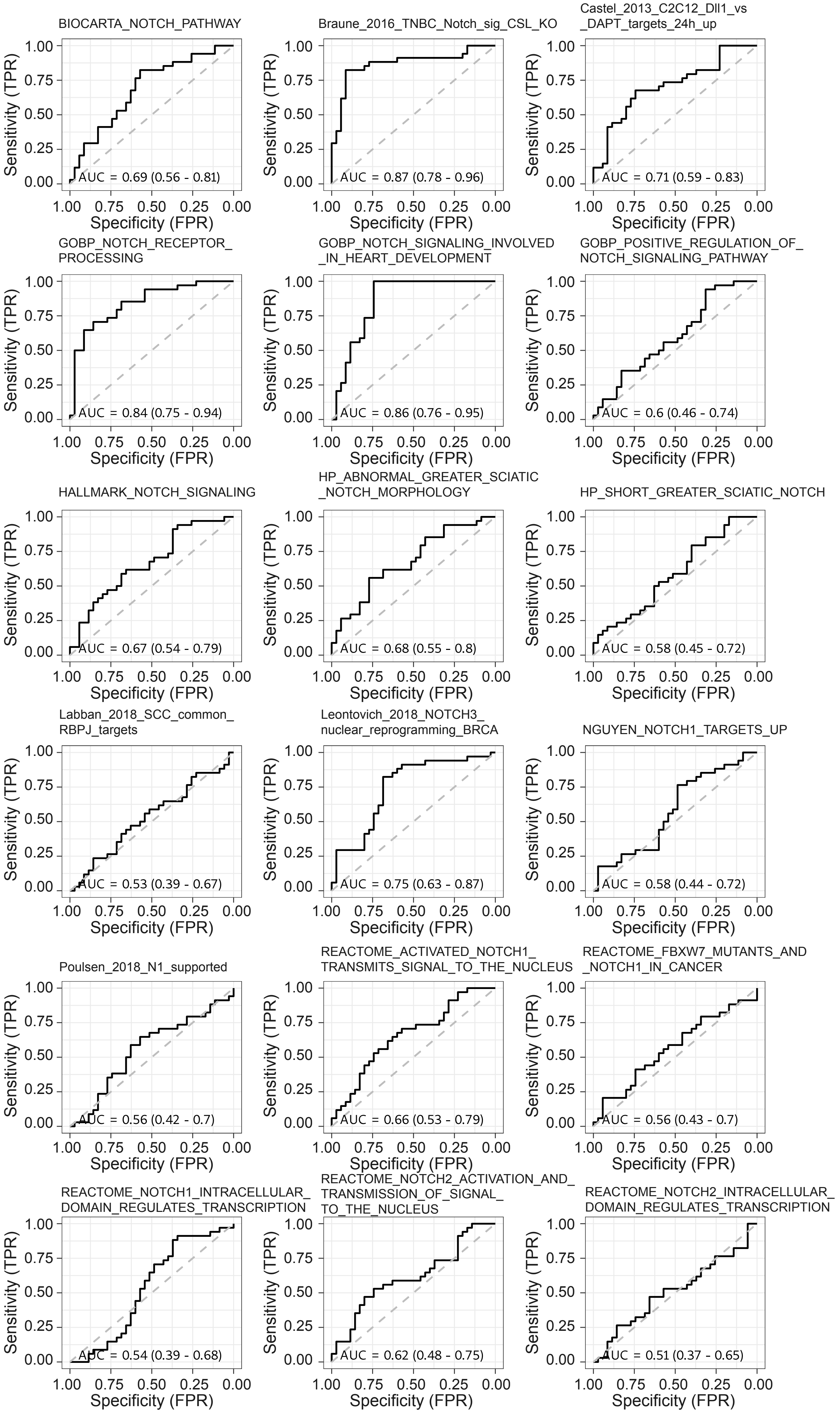

Fig. S6B continued

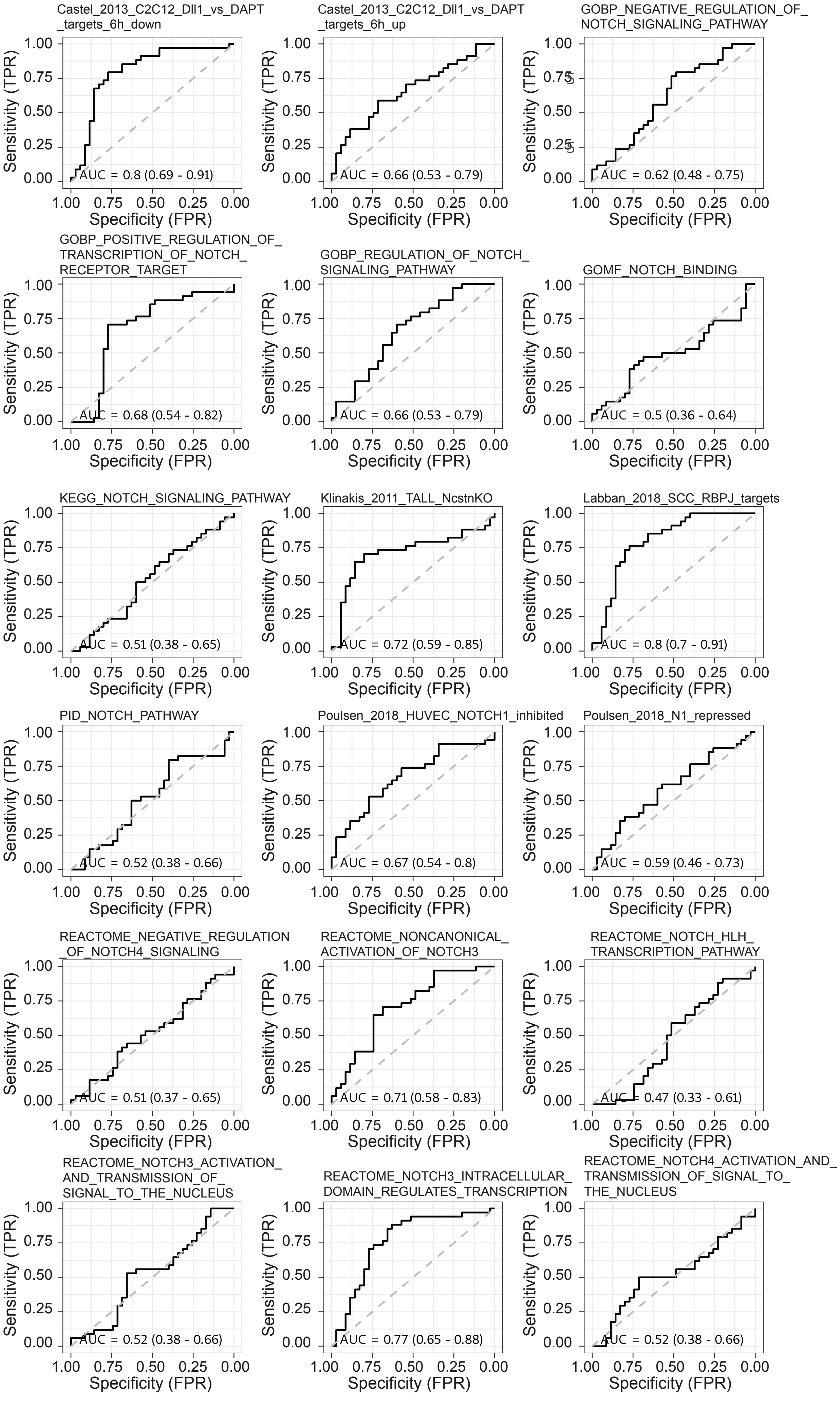

Fig. S6B continued

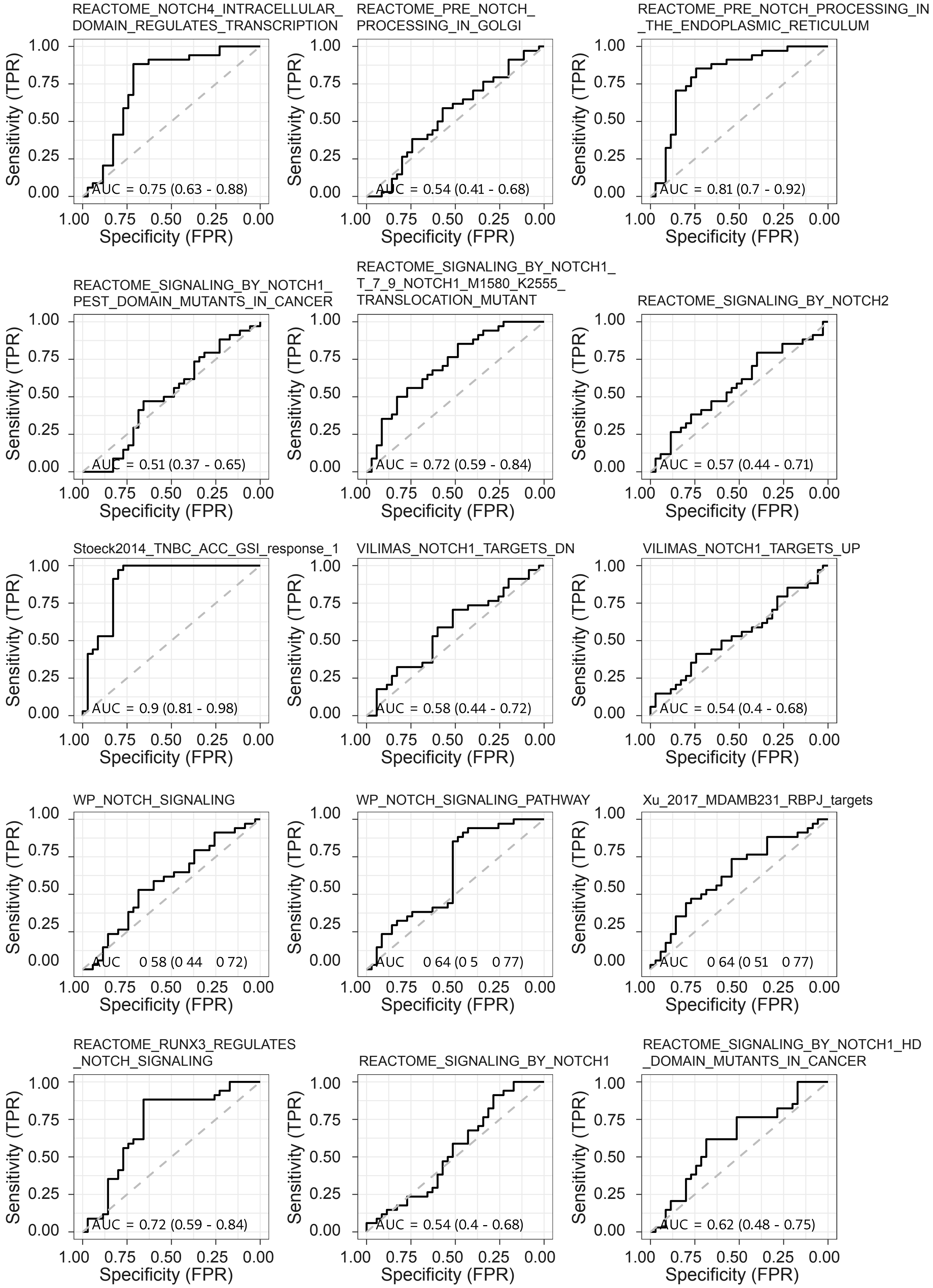

Fig. S6B continued

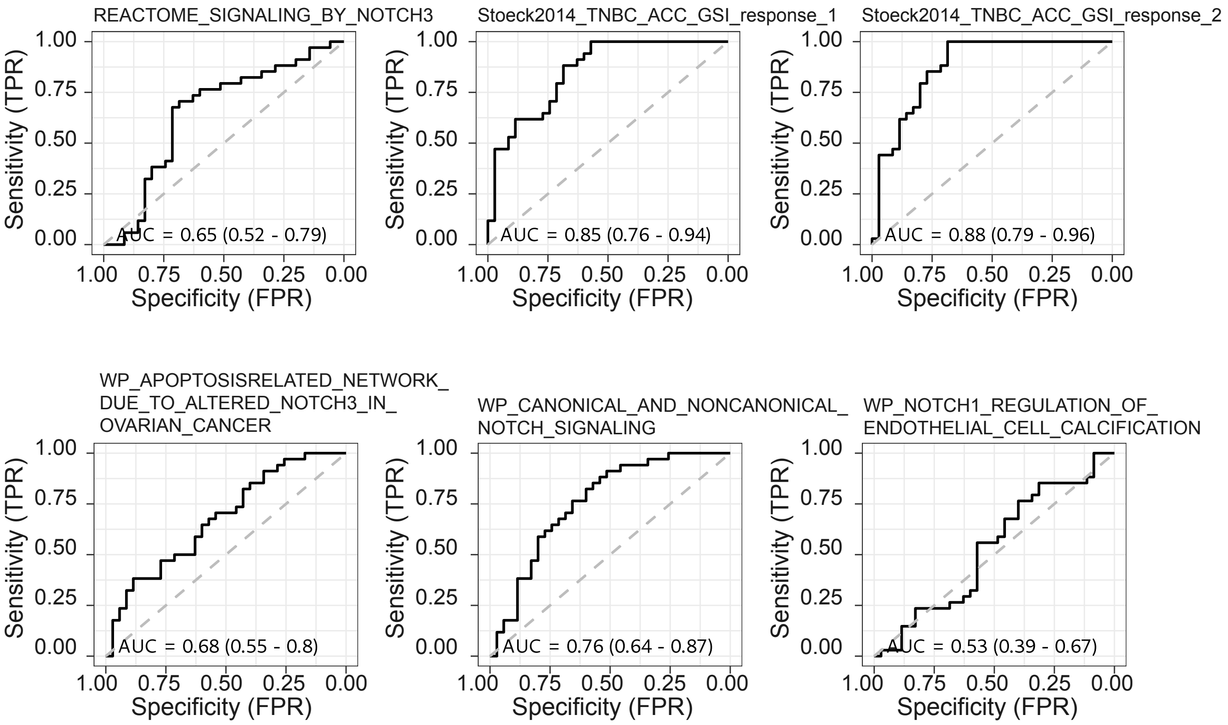

Fig. S6 continued
C

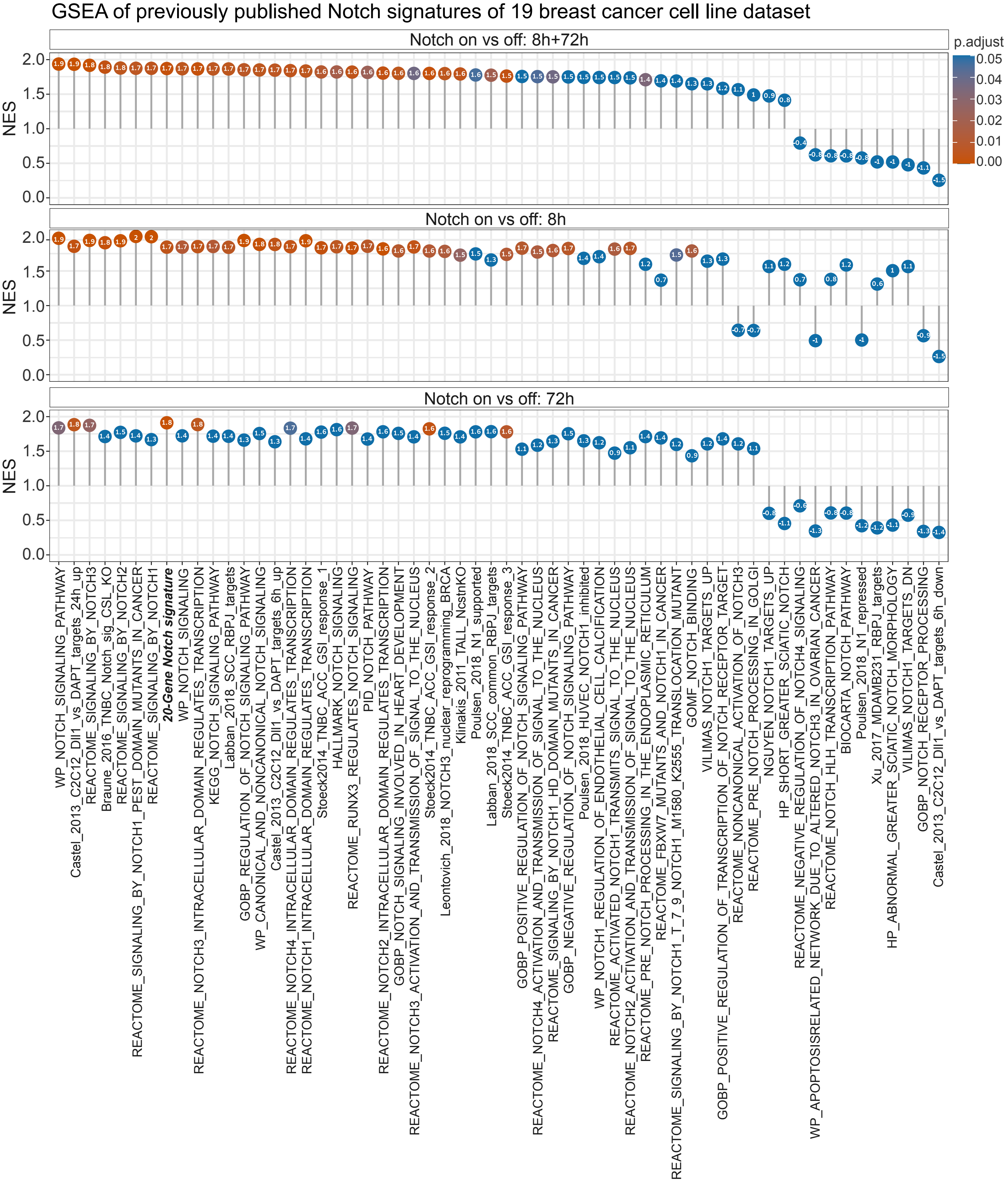

Fig. S6 continued

D

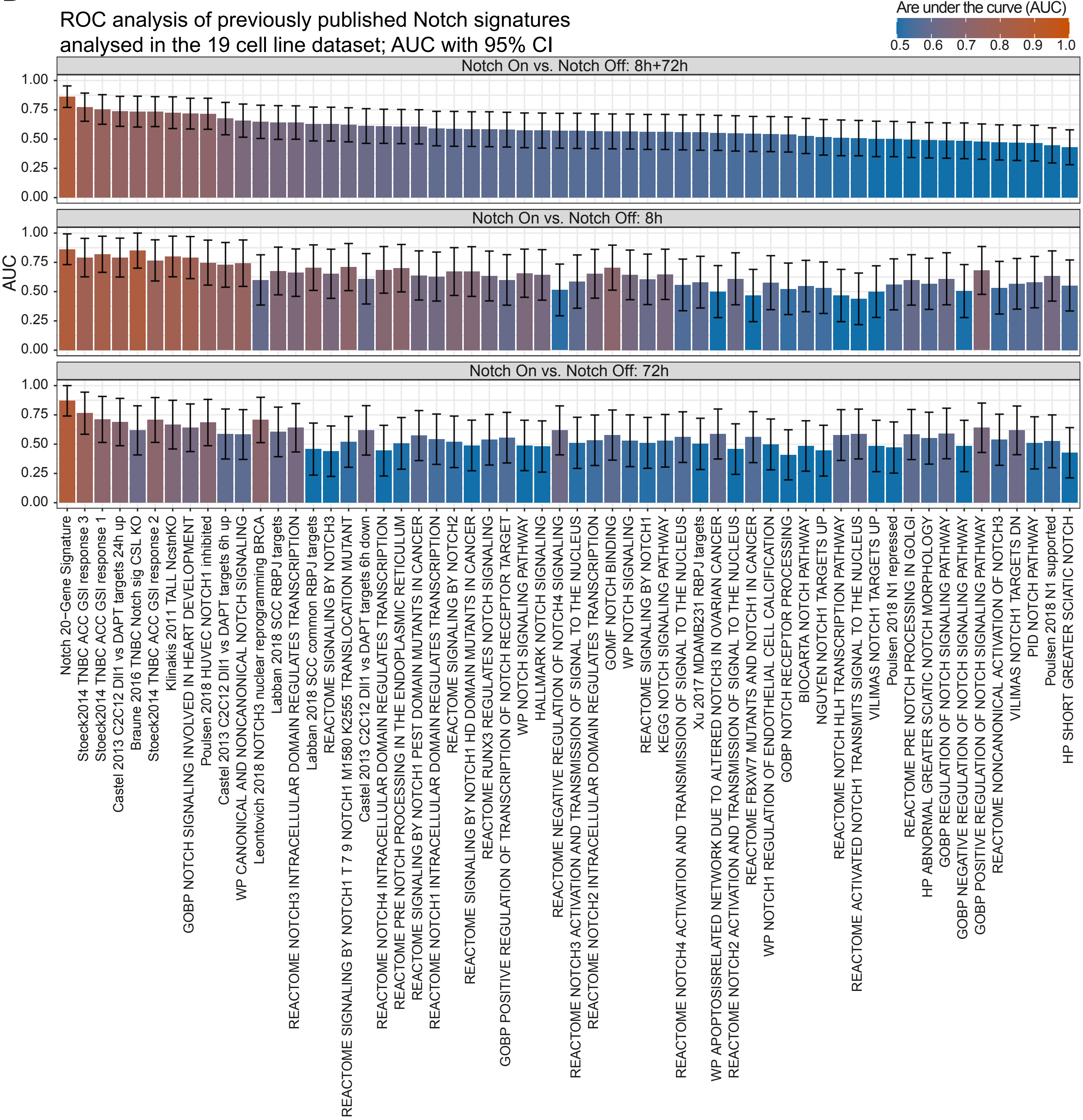

Fig. S6 continued

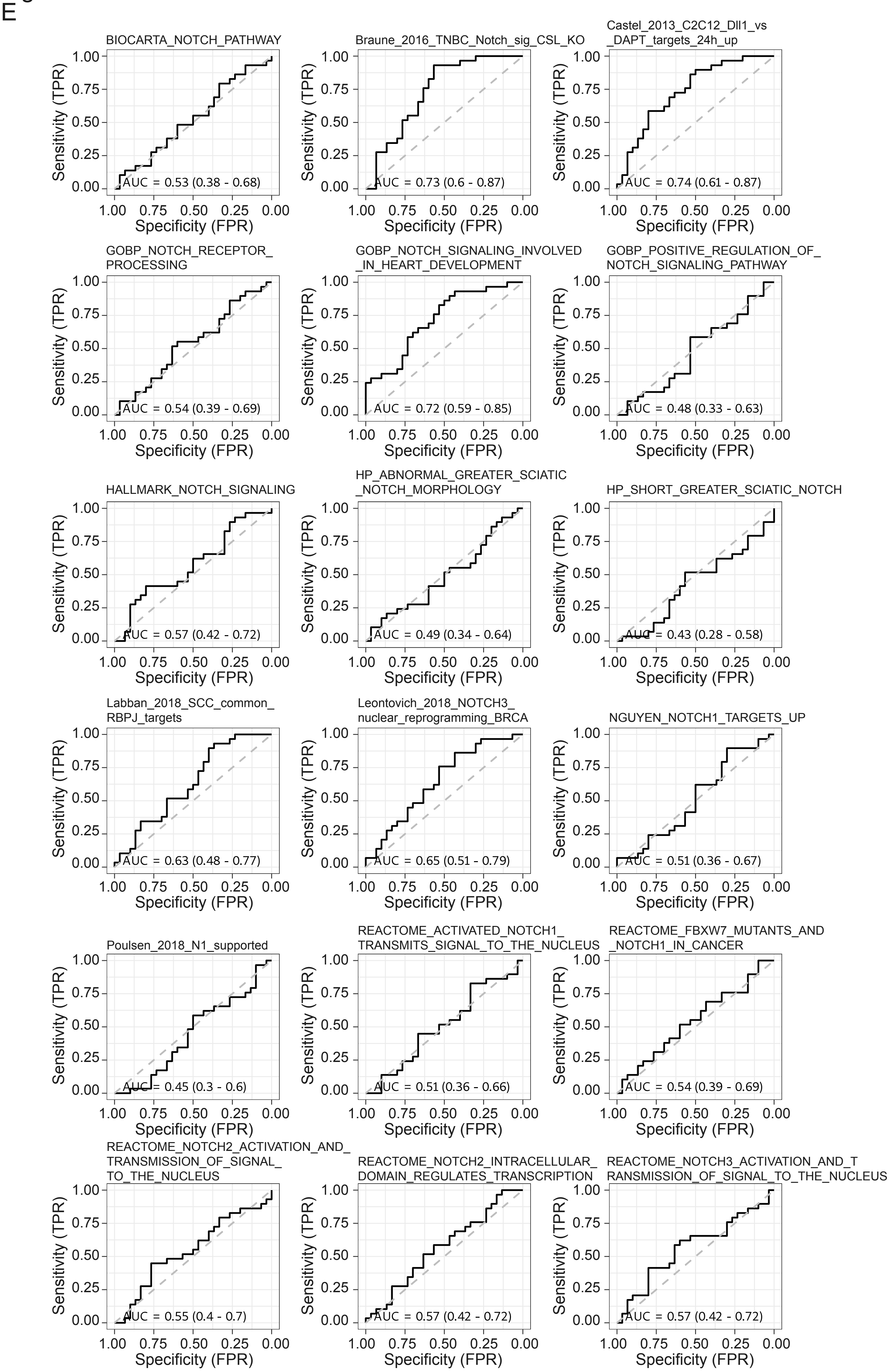

Fig. S6E continued

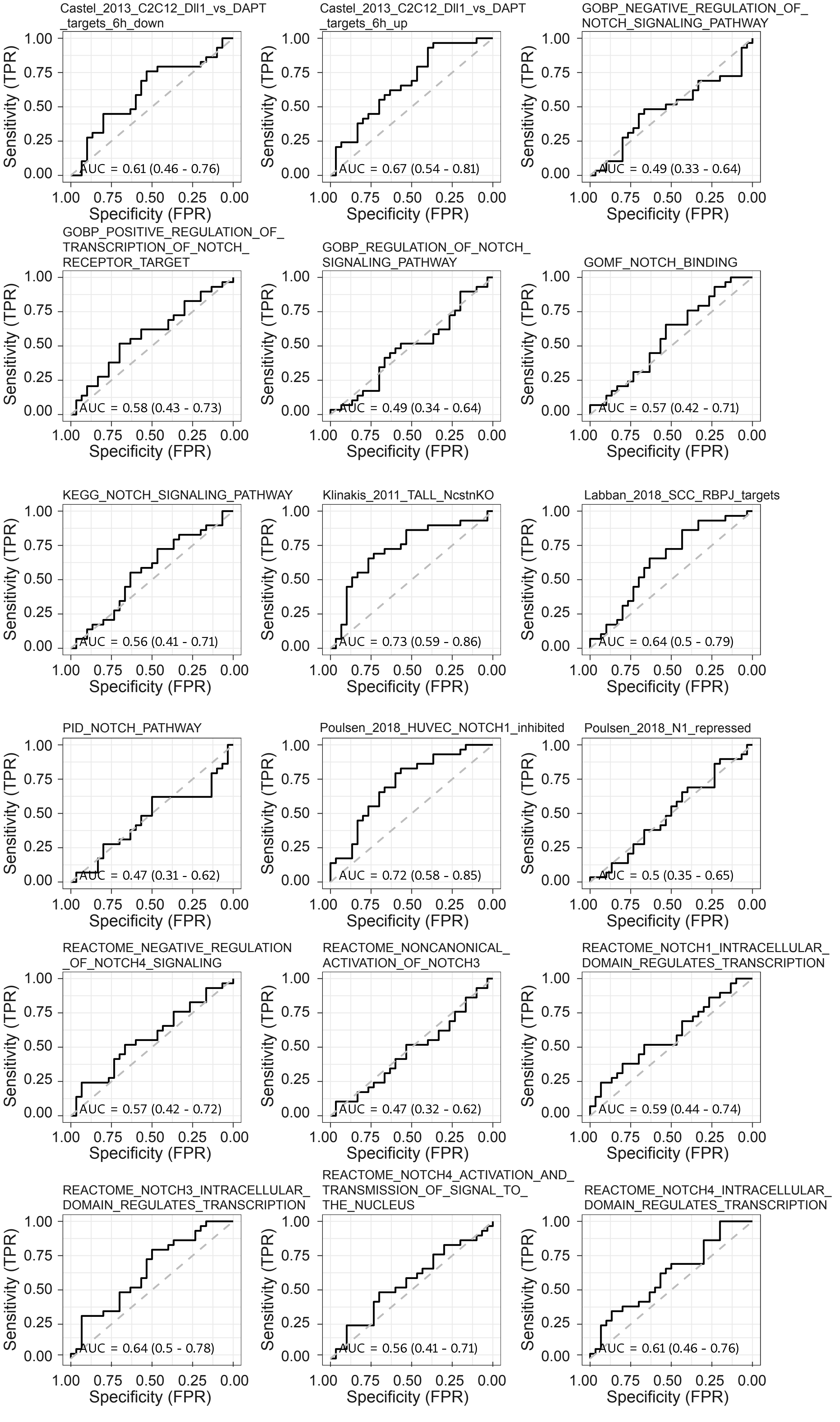

# Fig. S6E continued

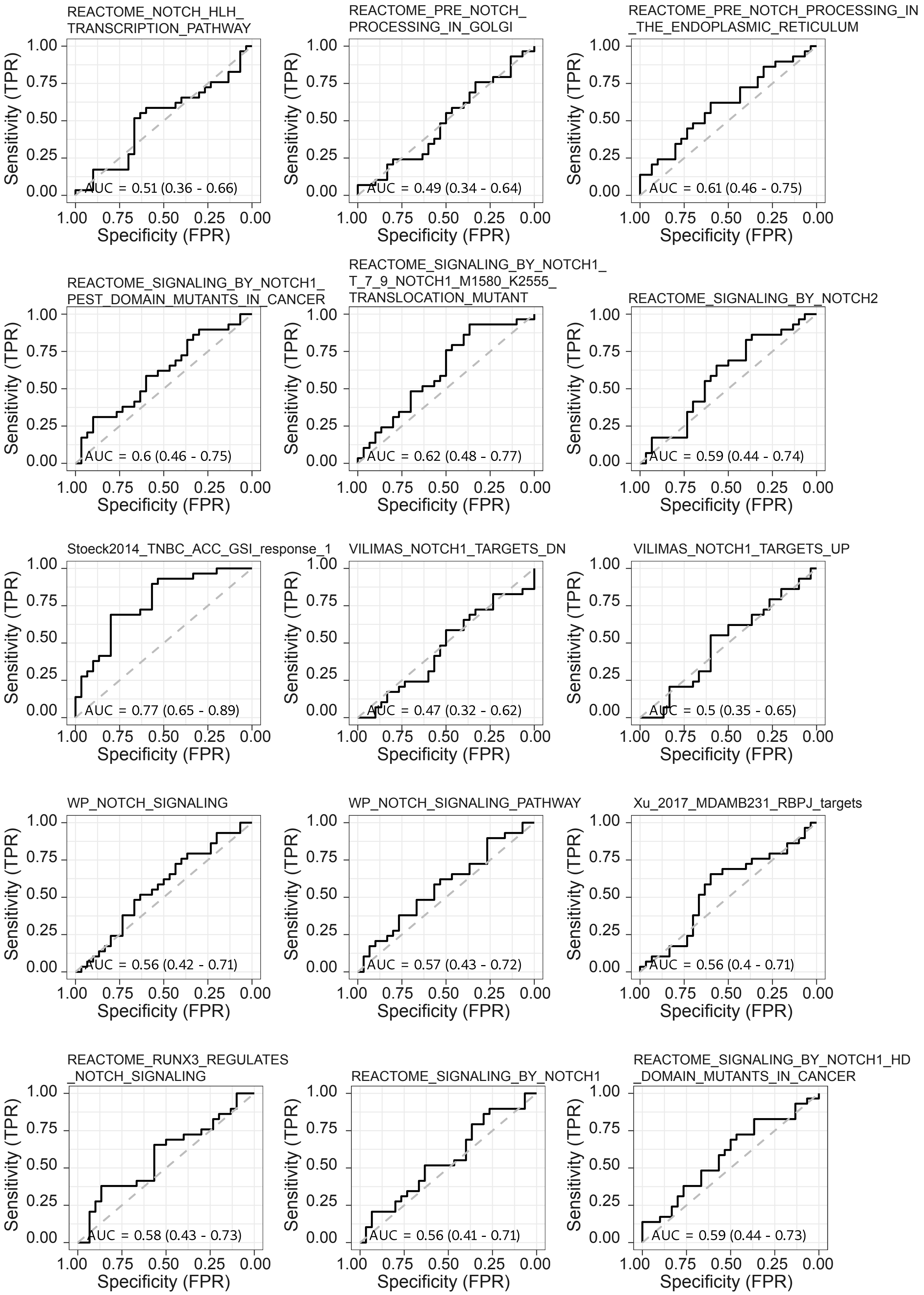

# Fig. S6E continued

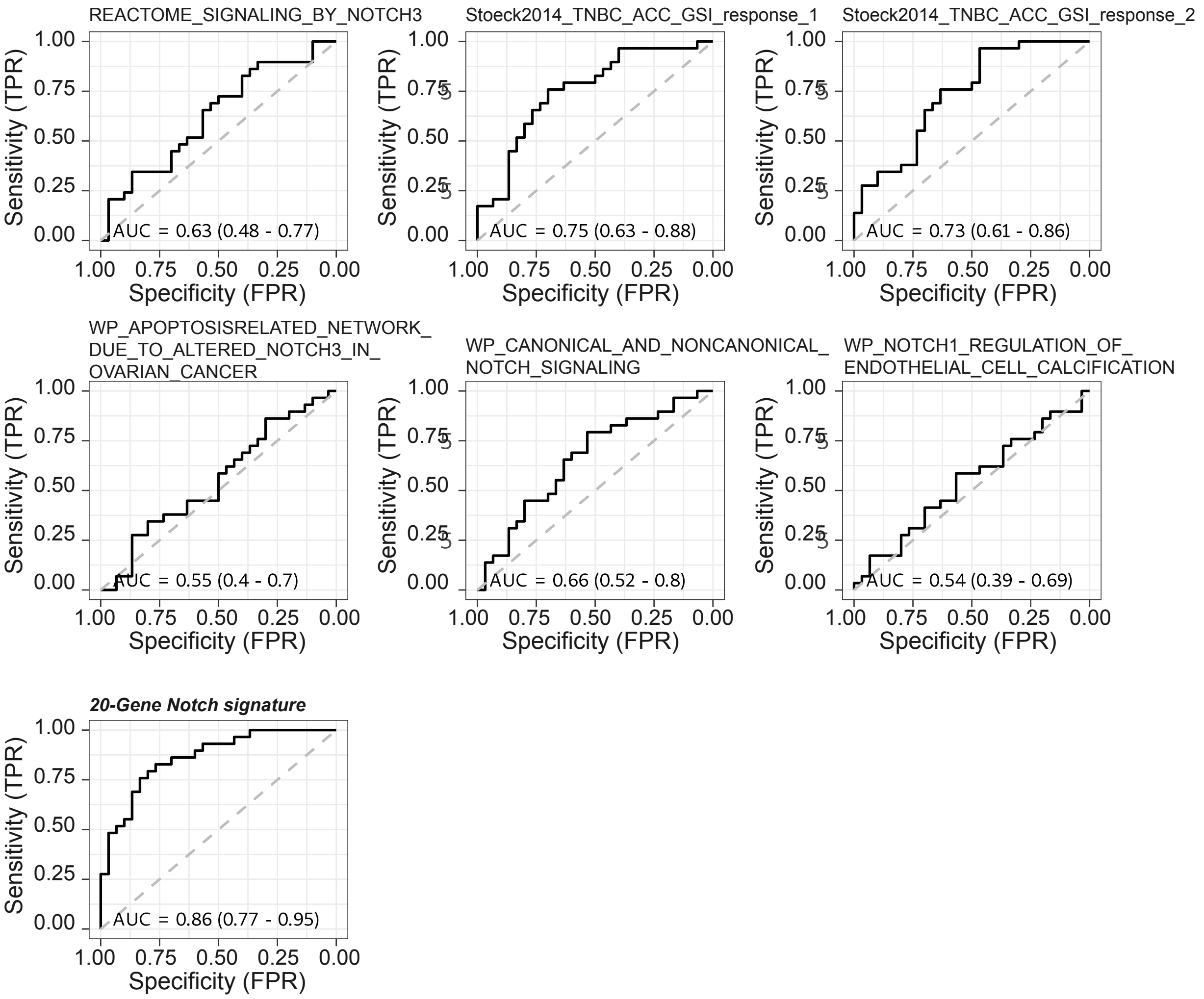

Fig. S7

A

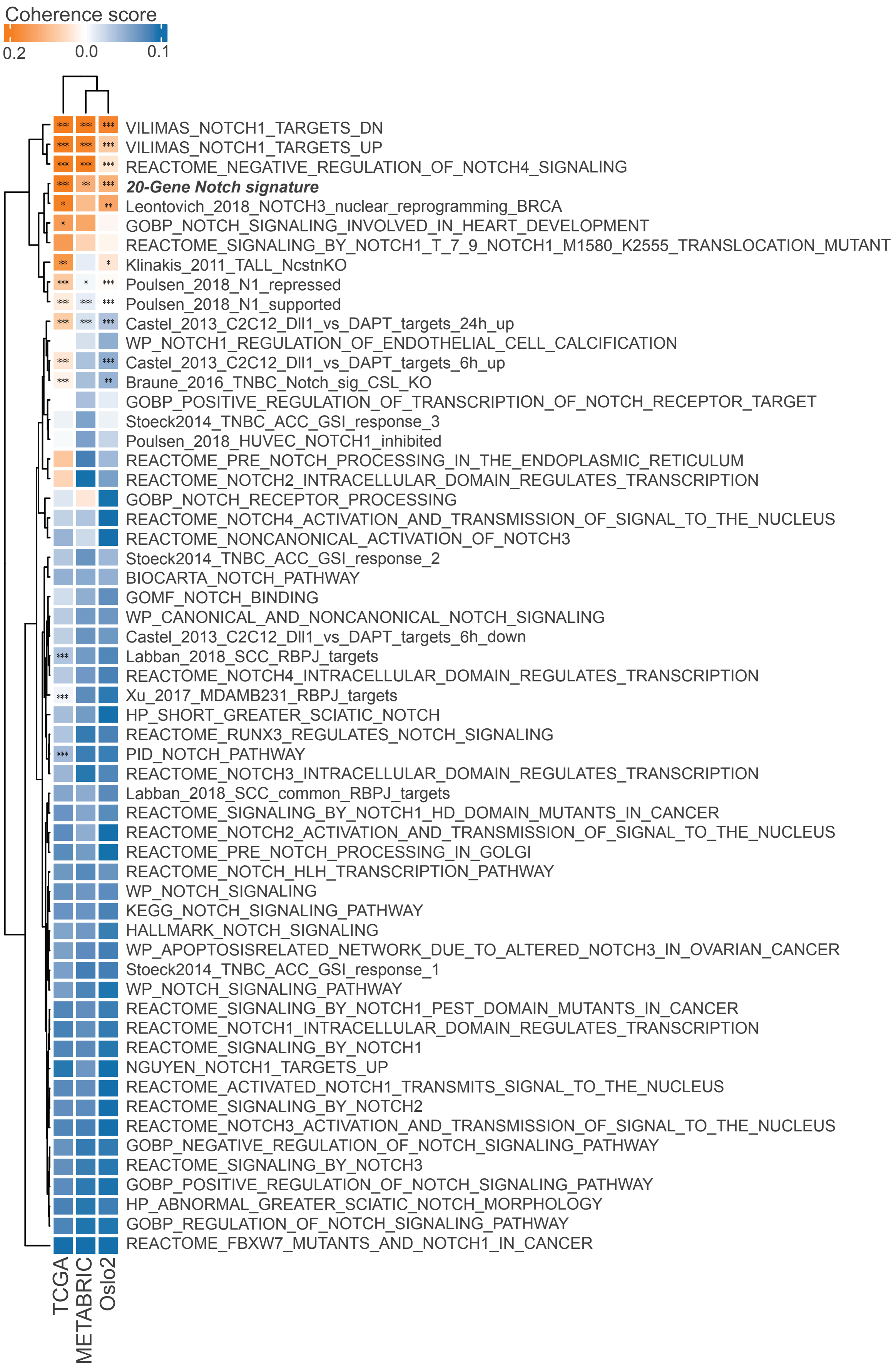

Coherence score
0.2
0.0
0.1
VILIMAS_NOTCH1_TARGETS_DN
VILIMAS_NOTCH1_TARGETS_UP
REACTOME_NEGATIVE_REGULATION_OF_NOTCH4_SIGNALING
20-Gene Notch signature
Leontovich_2018_NOTCH3_nuclear_reprogramming_BRCA
GOBP_NOTCH_SIGNALING_INVOLVED_IN_HEART_DEVELOPMENT
REACTOME_SIGNALING_BY_NOTCH1_T_7_9_NOTCH1_M1580_K2555_TRANSLOCATION_MUTANT
Klinakis_2011_TALL_NcstnKO
Poulsen_2018_N1_repressed
Poulsen_2018_N1_supported
Castel_2013_C2C12_Dll1_vs_DAPT_targets_24h_up
WP_NOTCH1_REGULATION_OF_ENDOTHELIAL_CELL_CALCIFICATION
Castel_2013_C2C12_Dll1_vs_DAPT_targets_6h_up
Braune_2016_TNBC_Notch_sig_CSL_KO
GOBP_POSITIVE_REGULATION_OF_TRANSCRIPTION_OF_NOTCH_RECEPTOR_TARGET
Stoeck2014_TNBC_ACC_GSI_response_3
Poulsen_2018_HUVEC_NOTCH1_inhibited
REACTOME_PRE_NOTCH_PROCESSING_IN_THE_ENDOPLASMIC_RETICULUM
REACTOME_NOTCH2_INTRACELLULAR_DOMAIN_REGULATES_TRANSCRIPTION
GOBP_NOTCH_RECEPTOR_PROCESSING
REACTOME_NOTCH4_ACTIVATION_AND_TRANSMISSION_OF_SIGNAL_TO_THE_NUCLEUS
REACTOME_NONCANONICAL_ACTIVATION_OF_NOTCH3
Stoeck2014_TNBC_ACC_GSI_response_2
BIOCARTA_NOTCH_PATHWAY
GOMF_NOTCH_BINDING
WP_CANONICAL_AND_NONCANONICAL_NOTCH_SIGNALING
Castel_2013_C2C12_Dll1_vs_DAPT_targets_6h_down
Labban_2018_SCC_RBPJ_targets
REACTOME_NOTCH4_INTRACELLULAR_DOMAIN_REGULATES_TRANSCRIPTION
Xu_2017_MDAMB231_RBPJ_targets
HP_SHORT_GREATER_SCIATIC_NOTCH
REACTOME_RUNX3_REGULATES_NOTCH_SIGNALING
PID_NOTCH_PATHWAY
REACTOME_NOTCH3_INTRACELLULAR_DOMAIN_REGULATES_TRANSCRIPTION
Labban_2018_SCC_common_RBPJ_targets
REACTOME_SIGNALING_BY_NOTCH1_HD_DOMAIN_MUTANTS_IN_CANCER
REACTOME_NOTCH2_ACTIVATION_AND_TRANSMISSION_OF_SIGNAL_TO_THE_NUCLEUS
REACTOME_PRE_NOTCH_PROCESSING_IN_GOLGI
REACTOME_NOTCH_HLH_TRANSCRIPTION_PATHWAY
WP_NOTCH_SIGNALING
KEGG_NOTCH_SIGNALING_PATHWAY
HALLMARK_NOTCH_SIGNALING
WP_APOPTOSISRELATED_NETWORK_DUE_TO_ALTERED_NOTCH3_IN_OVARIAN_CANCER
Stoeck2014_TNBC_ACC_GSI_response_1
WP_NOTCH_SIGNALING_PATHWAY
REACTOME_SIGNALING_BY_NOTCH1_PEST_DOMAIN_MUTANTS_IN_CANCER
REACTOME_NOTCH1_INTRACELLULAR_DOMAIN_REGULATES_TRANSCRIPTION
REACTOME_SIGNALING_BY_NOTCH1
NGUYEN_NOTCH1_TARGETS_UP
REACTOME_ACTIVATED_NOTCH1_TRANSMITS_SIGNAL_TO_THE_NUCLEUS
REACTOME_SIGNALING_BY_NOTCH2
REACTOME_NOTCH3_ACTIVATION_AND_TRANSMISSION_OF_SIGNAL_TO_THE_NUCLEUS
GOBP_NEGATIVE_REGULATION_OF_NOTCH_SIGNALING_PATHWAY
REACTOME_SIGNALING_BY_NOTCH3
GOBP_POSITIVE_REGULATION_OF_NOTCH_SIGNALING_PATHWAY
HP_ABNORMAL_GREATER_SCIATIC_NOTCH_MORPHOLOGY
GOBP_REGULATION_OF_NOTCH_SIGNALING_PATHWAY
REACTOME_FBXW7_MUTANTS_AND_NOTCH1_IN_CANCER
TCGA
METABRIC
Oslo2

Fig. S7 continued

B

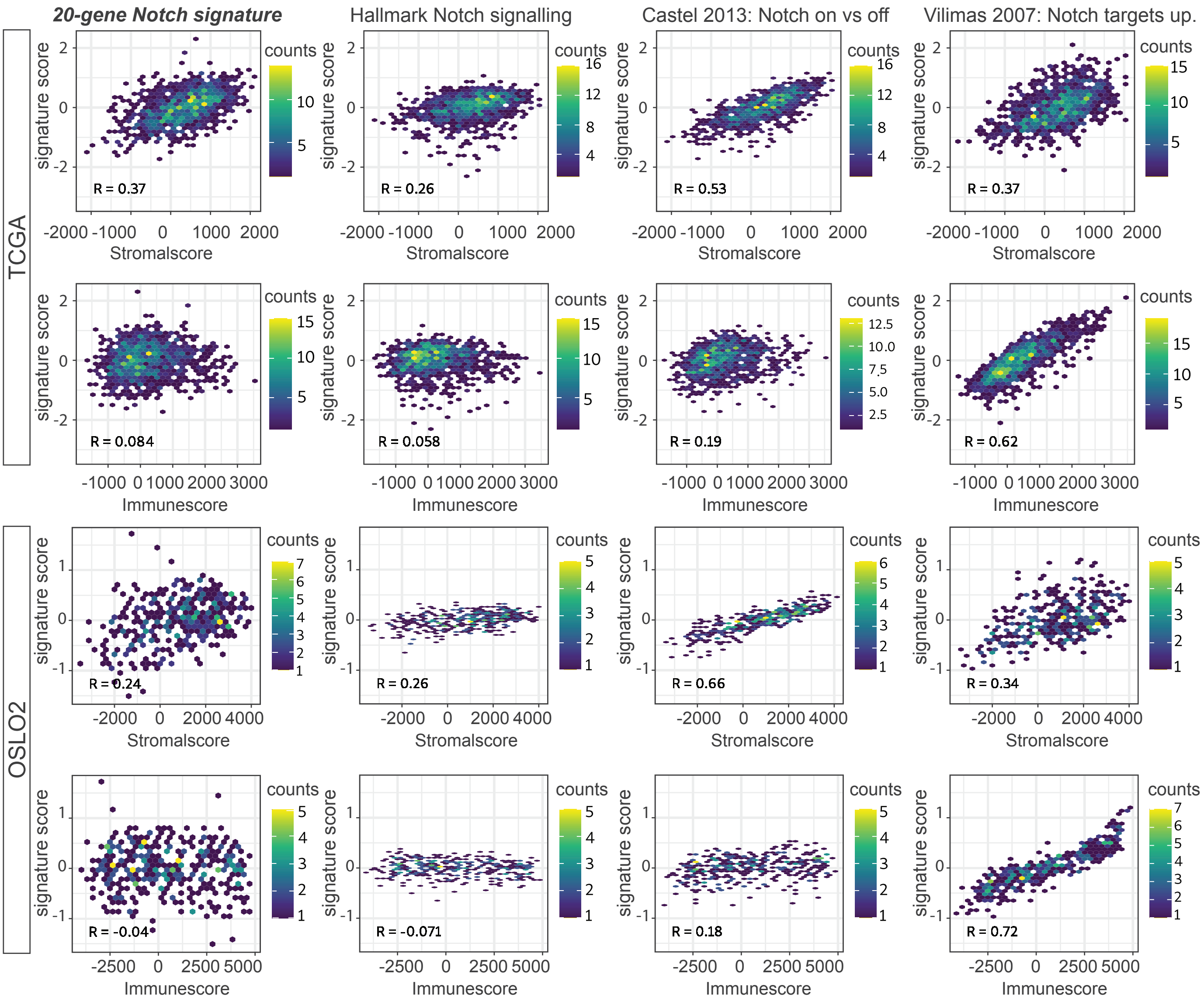

Fig. S8

A Correlation of NOTCH signatures in breast cancer cell lines at ground state to the NOTCH Core Signature

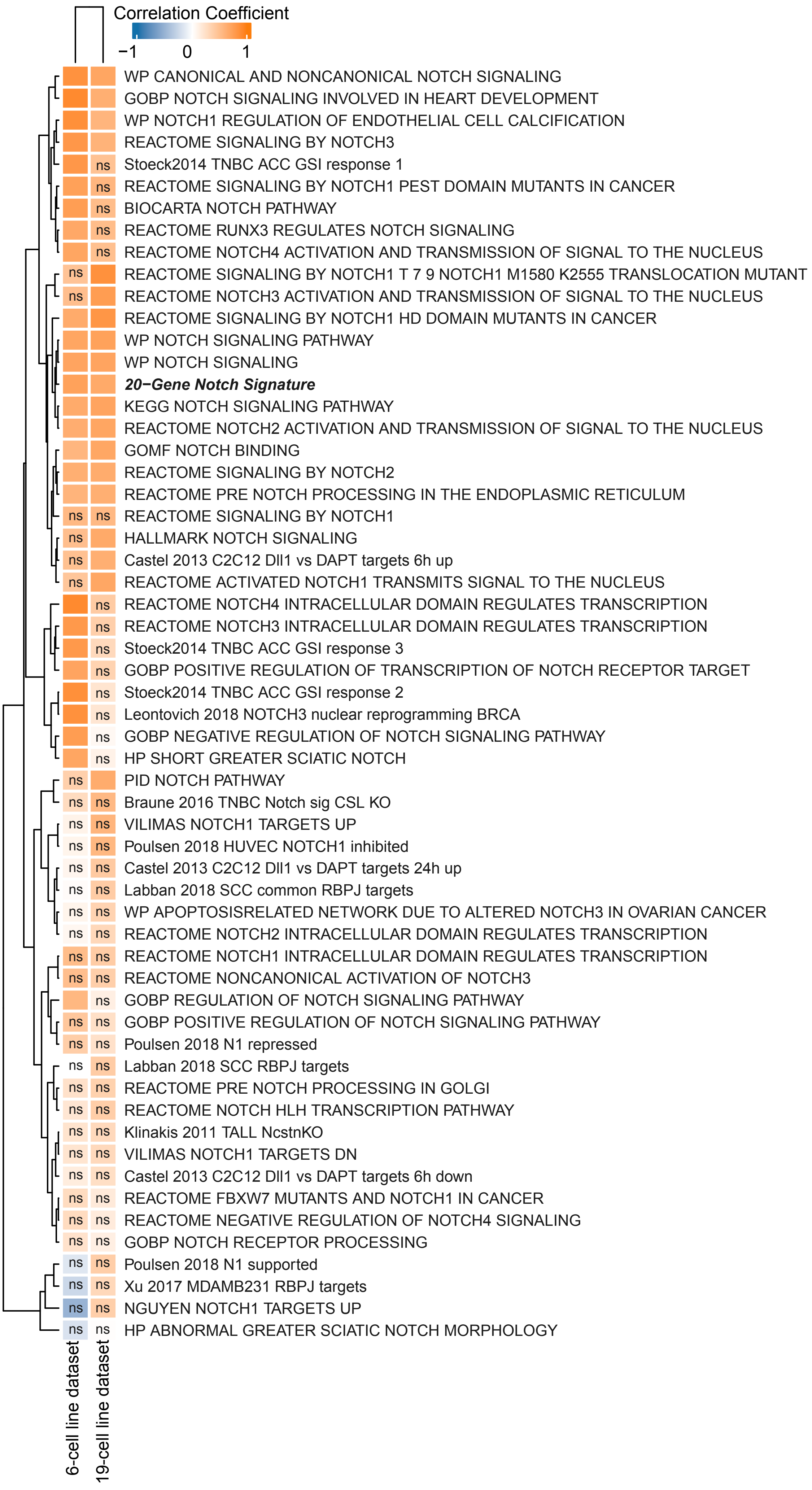

Fig. S8 continued

B

Correlation of NOTCH signatures in patient cohorts to the NOTCH Core Signature

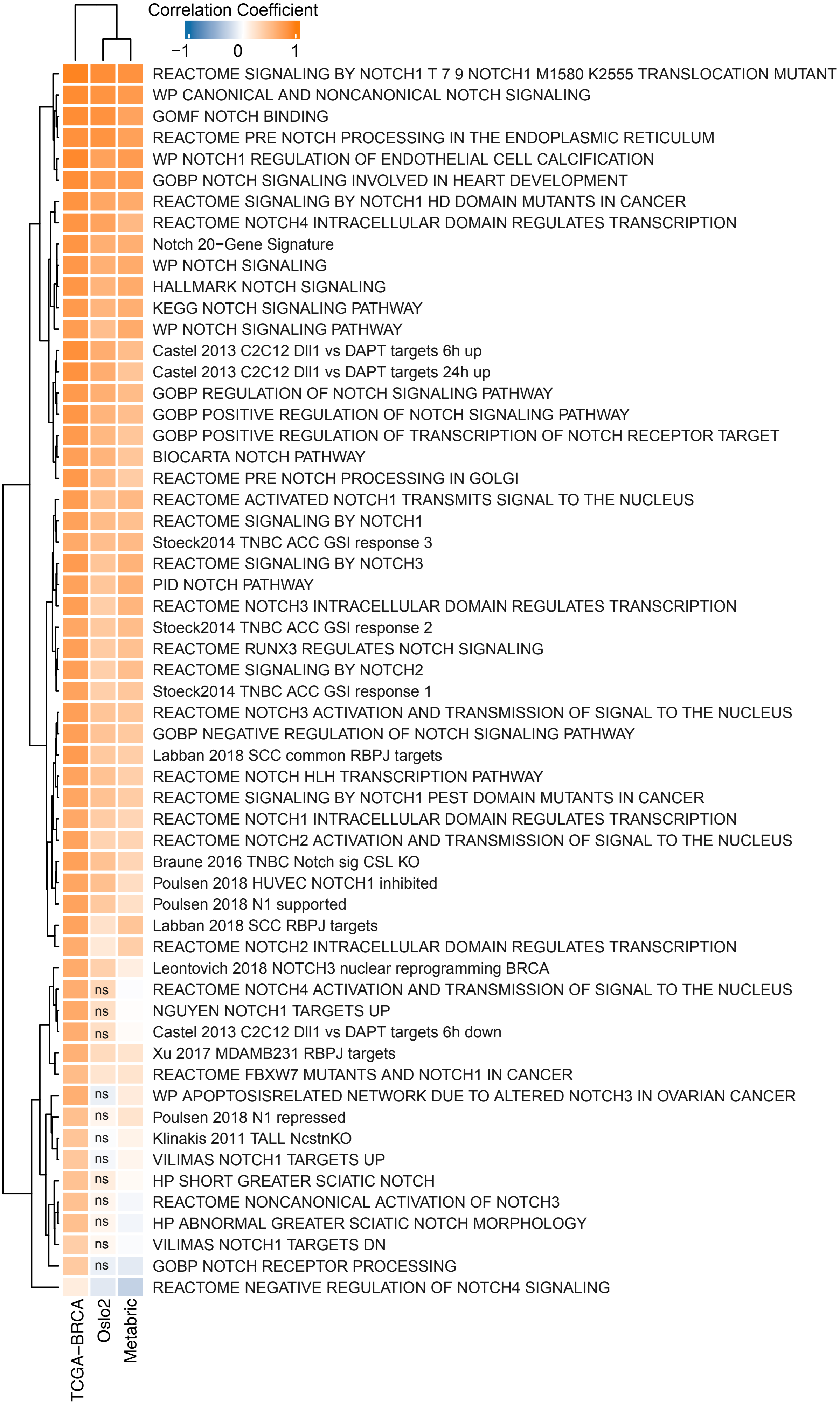

Fig. S8 continued
C

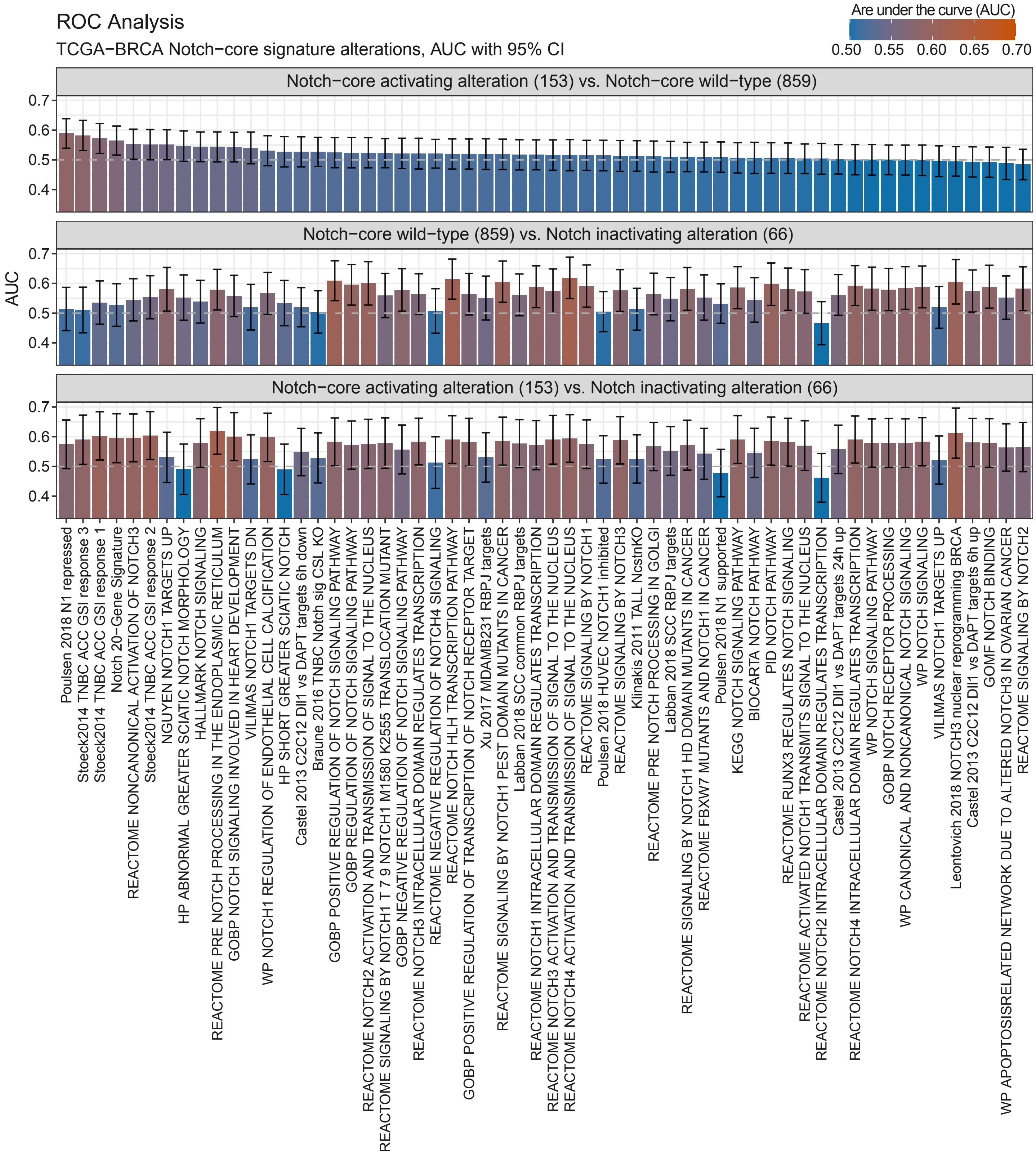

Fig. S8 continued

D

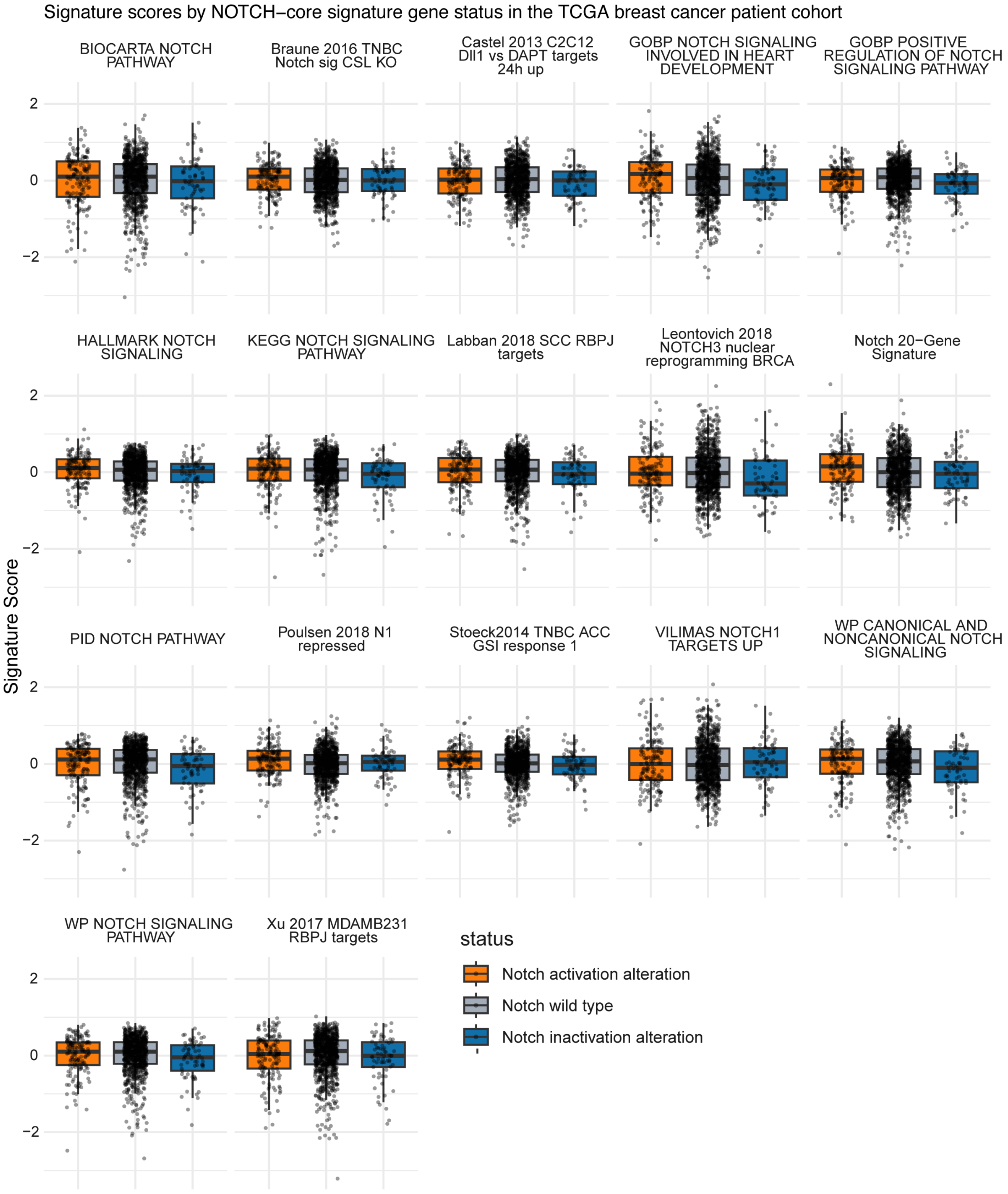

Fig. S9

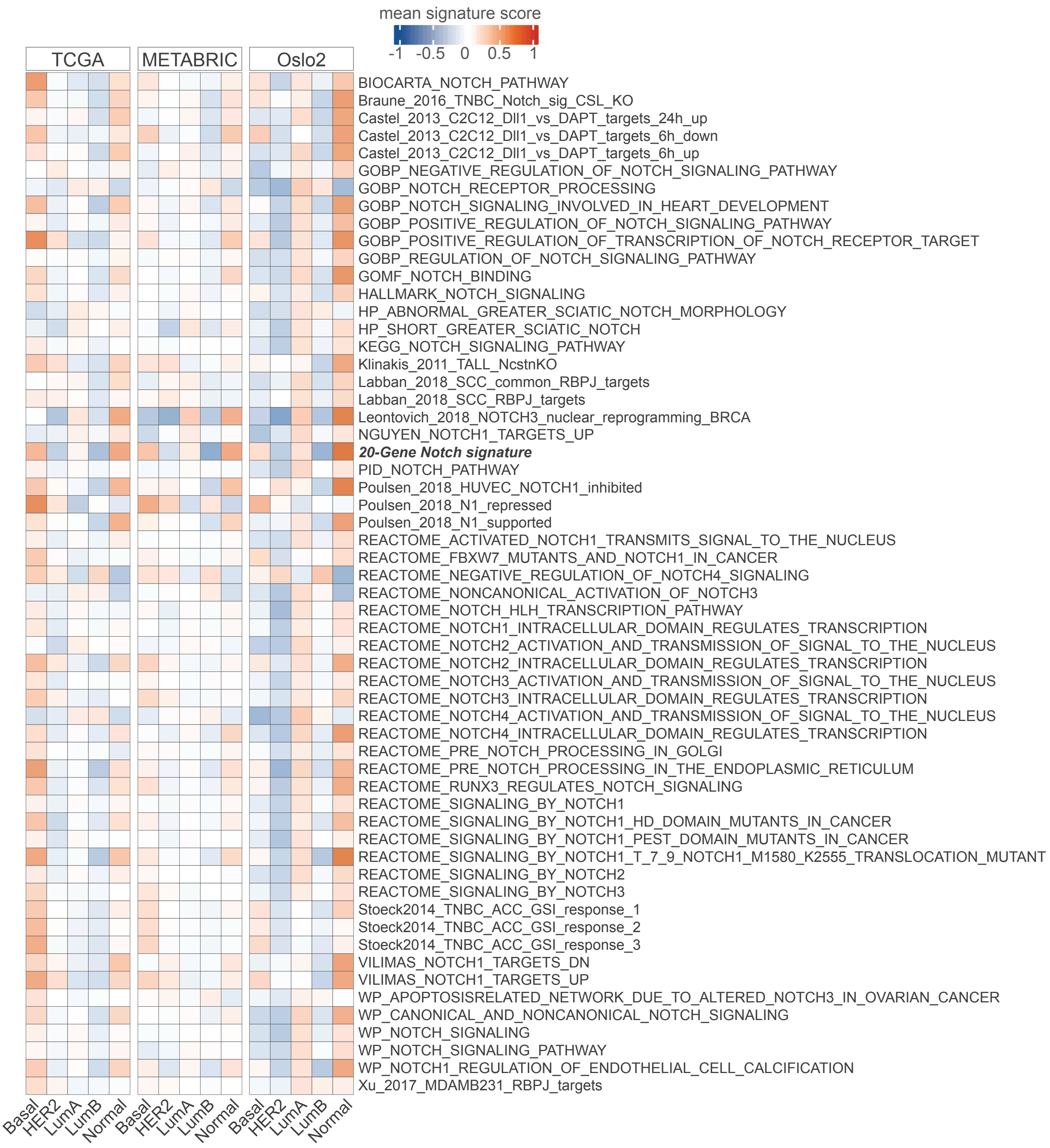
mean signature score
-1 -0.5 0 0.5 1
TCGA
METABRIC
Oslo2
BIOCARTA_NOTCH_PATHWAY
Braune_2016_TNBC_Notch_sig_CSL_KO
Castel_2013_C2C12_Dll1_vs_DAPT_targets_24h_up
Castel_2013_C2C12_Dll1_vs_DAPT_targets_6h_down
Castel_2013_C2C12_Dll1_vs_DAPT_targets_6h_up
GOBP_NEGATIVE_REGULATION_OF_NOTCH_SIGNALING_PATHWAY
GOBP_NOTCH_RECEPTOR_PROCESSING
GOBP_NOTCH_SIGNALING_INVOLVED_IN_HEART_DEVELOPMENT
GOBP_POSITIVE_REGULATION_OF_NOTCH_SIGNALING_PATHWAY
GOBP_POSITIVE_REGULATION_OF_TRANSCRIPTION_OF_NOTCH_RECEPTOR_TARGET
GOBP_REGULATION_OF_NOTCH_SIGNALING_PATHWAY
GOMF_NOTCH_BINDING
HALLMARK_NOTCH_SIGNALING
HP_ABNORMAL_GREATER_SCIATIC_NOTCH_MORPHOLOGY
HP_SHORT_GREATER_SCIATIC_NOTCH
KEGG_NOTCH_SIGNALING_PATHWAY
Klinakis_2011_TALL_NcstnKO
Labban_2018_SCC_common_RBPJ_targets
Labban_2018_SCC_RBPJ_targets
Leontovich_2018_NOTCH3_nuclear_reprogramming_BRCA
NGUYEN_NOTCH1_TARGETS_UP
20-Gene Notch signature
PID_NOTCH_PATHWAY
Poulsen_2018_HUVEC_NOTCH1_inhibited
Poulsen_2018_N1_repressed
Poulsen_2018_N1_supported
REACTOME_ACTIVATED_NOTCH1_TRANSMITS_SIGNAL_TO_THE_NUCLEUS
REACTOME_FBXW7_MUTANTS_AND_NOTCH1_IN_CANCER
REACTOME_NEGATIVE_REGULATION_OF_NOTCH4_SIGNALING
REACTOME_NONCANONICAL_ACTIVATION_OF_NOTCH3
REACTOME_NOTCH_HLH_TRANSCRIPTION_PATHWAY
REACTOME_NOTCH1_INTRACELLULAR_DOMAIN_REGULATES_TRANSCRIPTION
REACTOME_NOTCH2_ACTIVATION_AND_TRANSMISSION_OF_SIGNAL_TO_THE_NUCLEUS
REACTOME_NOTCH2_INTRACELLULAR_DOMAIN_REGULATES_TRANSCRIPTION
REACTOME_NOTCH3_ACTIVATION_AND_TRANSMISSION_OF_SIGNAL_TO_THE_NUCLEUS
REACTOME_NOTCH3_INTRACELLULAR_DOMAIN_REGULATES_TRANSCRIPTION
REACTOME_NOTCH4_ACTIVATION_AND_TRANSMISSION_OF_SIGNAL_TO_THE_NUCLEUS
REACTOME_NOTCH4_INTRACELLULAR_DOMAIN_REGULATES_TRANSCRIPTION
REACTOME_PRE_NOTCH_PROCESSING_IN_GOLGI
REACTOME_PRE_NOTCH_PROCESSING_IN_THE_ENDOPLASMIC_RETICULUM
REACTOME_RUNX3_REGULATES_NOTCH_SIGNALING
REACTOME_SIGNALING_BY_NOTCH1
REACTOME_SIGNALING_BY_NOTCH1_HD_DOMAIN_MUTANTS_IN_CANCER
REACTOME_SIGNALING_BY_NOTCH1_PEST_DOMAIN_MUTANTS_IN_CANCER
REACTOME_SIGNALING_BY_NOTCH1_T_7_9_NOTCH1_M1580_K2555_TRANSLOCATION_MUTANT
REACTOME_SIGNALING_BY_NOTCH2
REACTOME_SIGNALING_BY_NOTCH3
Stoeck2014_TNBC_ACC_GSI_response_1
Stoeck2014_TNBC_ACC_GSI_response_2
Stoeck2014_TNBC_ACC_GSI_response_3
VILIMAS_NOTCH1_TARGETS_DN
VILIMAS_NOTCH1_TARGETS_UP
WP_APOPTOSISRELATED_NETWORK_DUE_TO_ALTERED_NOTCH3_IN_OVARIAN_CANCER
WP_CANONICAL_AND_NONCANONICAL_NOTCH_SIGNALING
WP_NOTCH_SIGNALING
WP_NOTCH_SIGNALING_PATHWAY
WP_NOTCH1_REGULATION_OF_ENDOTHELIAL_CELL_CALCIFICATION
Xu_2017_MDAMB231_RBPJ_targets
Basal
HER2
LumA
LumB
Normal
Basal
HER2
LumA
LumB
Normal
Basal
HER2
LumA
LumB
Normal

Fig. S10

A

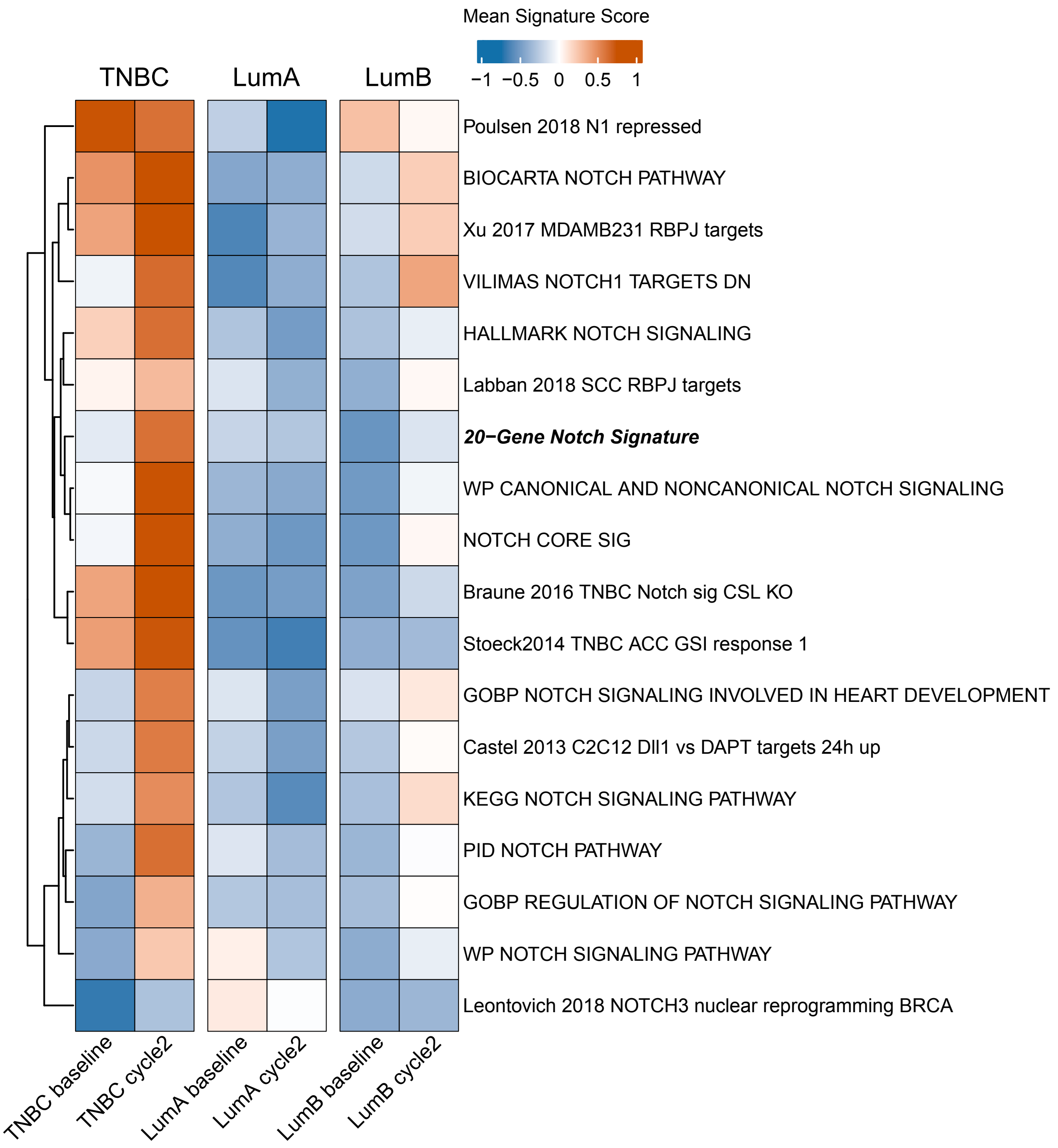

Mean Signature Score
−1 −0.5 0 0.5 1
TNBC
LumA
LumB
Poulsen 2018 N1 repressed
BIOCARTA NOTCH PATHWAY
Xu 2017 MDAMB231 RBPJ targets
VILIMAS NOTCH1 TARGETS DN
HALLMARK NOTCH SIGNALING
Labban 2018 SCC RBPJ targets
20−Gene Notch Signature
WP CANONICAL AND NONCANONICAL NOTCH SIGNALING
NOTCH CORE SIG
Braune 2016 TNBC Notch sig CSL KO
Stoeck2014 TNBC ACC GSI response 1
GOBP NOTCH SIGNALING INVOLVED IN HEART DEVELOPMENT
Castel 2013 C2C12 Dll1 vs DAPT targets 24h up
KEGG NOTCH SIGNALING PATHWAY
PID NOTCH PATHWAY
GOBP REGULATION OF NOTCH SIGNALING PATHWAY
WP NOTCH SIGNALING PATHWAY
Leontovich 2018 NOTCH3 nuclear reprogramming BRCA
TNBC baseline
TNBC cycle2
LumA baseline
LumA cycle2
LumB baseline
LumB cycle2


B

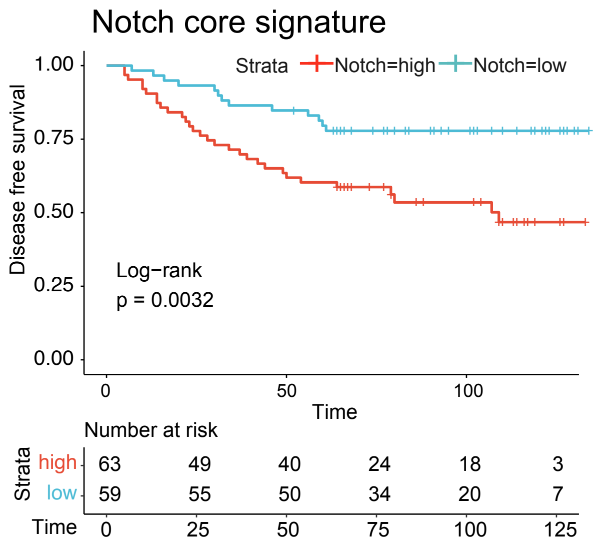

Notch core signature
Strata
Notch=high
Notch=low
1.00
0.75
0.50
0.25
0.00
Disease free survival
Log−rank
p = 0.0032
0
50
100
Time
Number at risk
Strata
high
low
63 49 40 24 18 3
59 55 50 34 20 7
Time
0 25 50 75 100 125


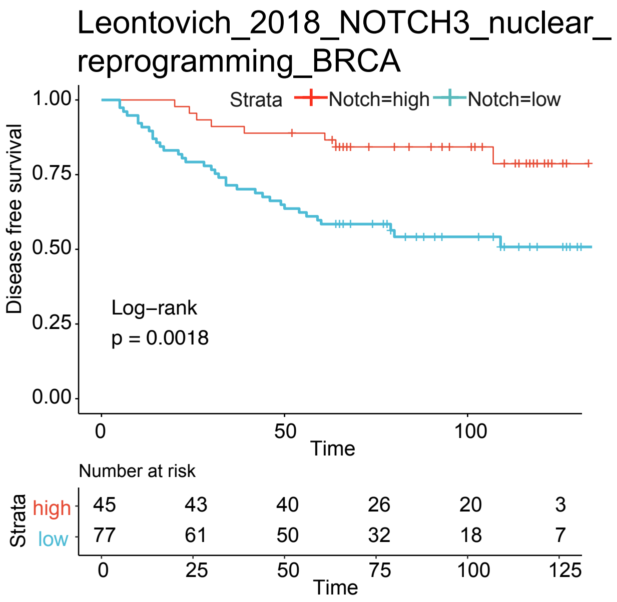

Leontovich_2018_NOTCH3_nuclear_
reprogramming_BRCA
Strata
Notch=high
Notch=low
1.00
0.75
0.50
0.25
0.00
Disease free survival
Log−rank
p = 0.0018
0
50
100
Time
Number at risk
Strata
high
low
45 43 40 26 20 3
77 61 50 32 18 7
0 25 50 75 100 125
Time

Fig. S10B continued

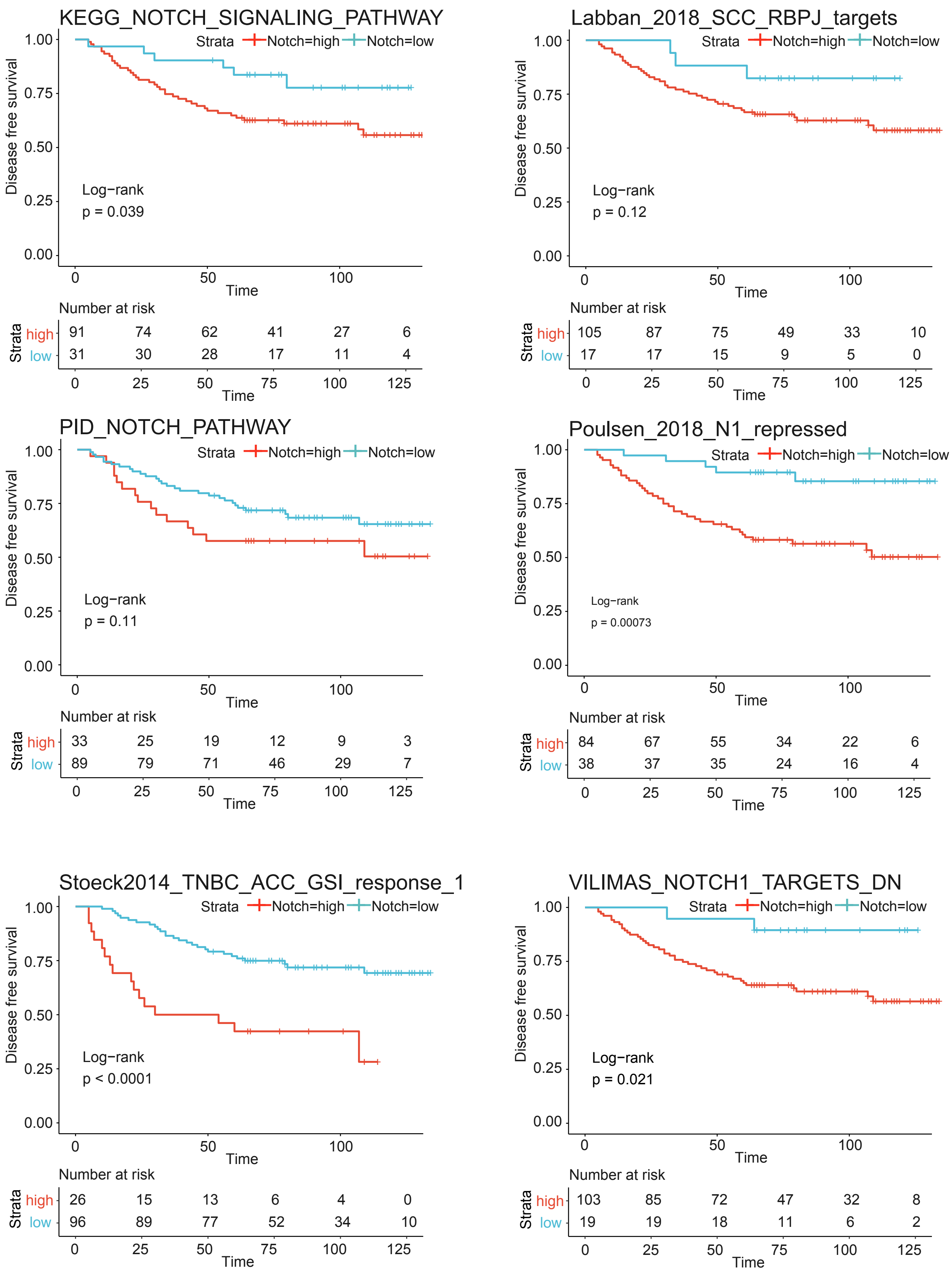

Fig. S10B continued

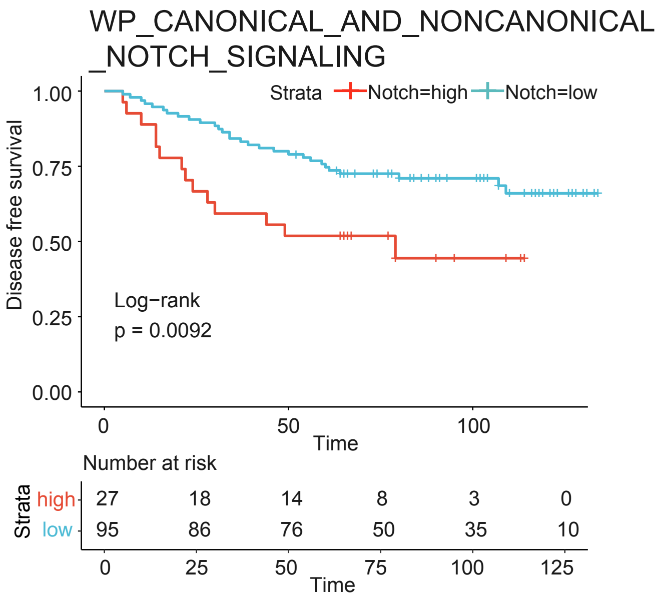


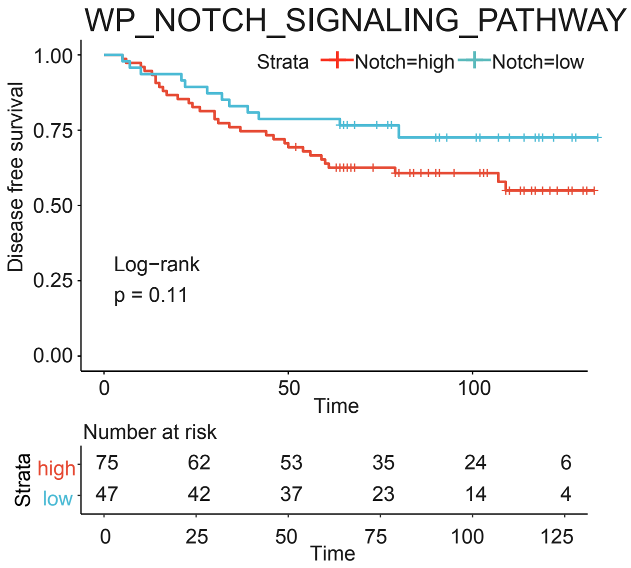


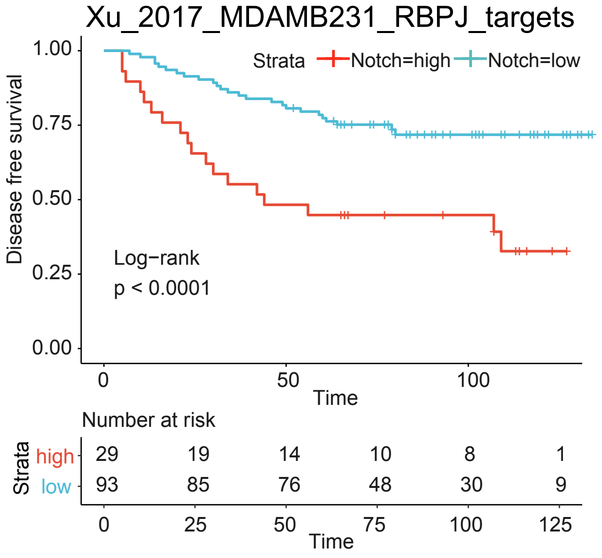

# Fig. S10B continued

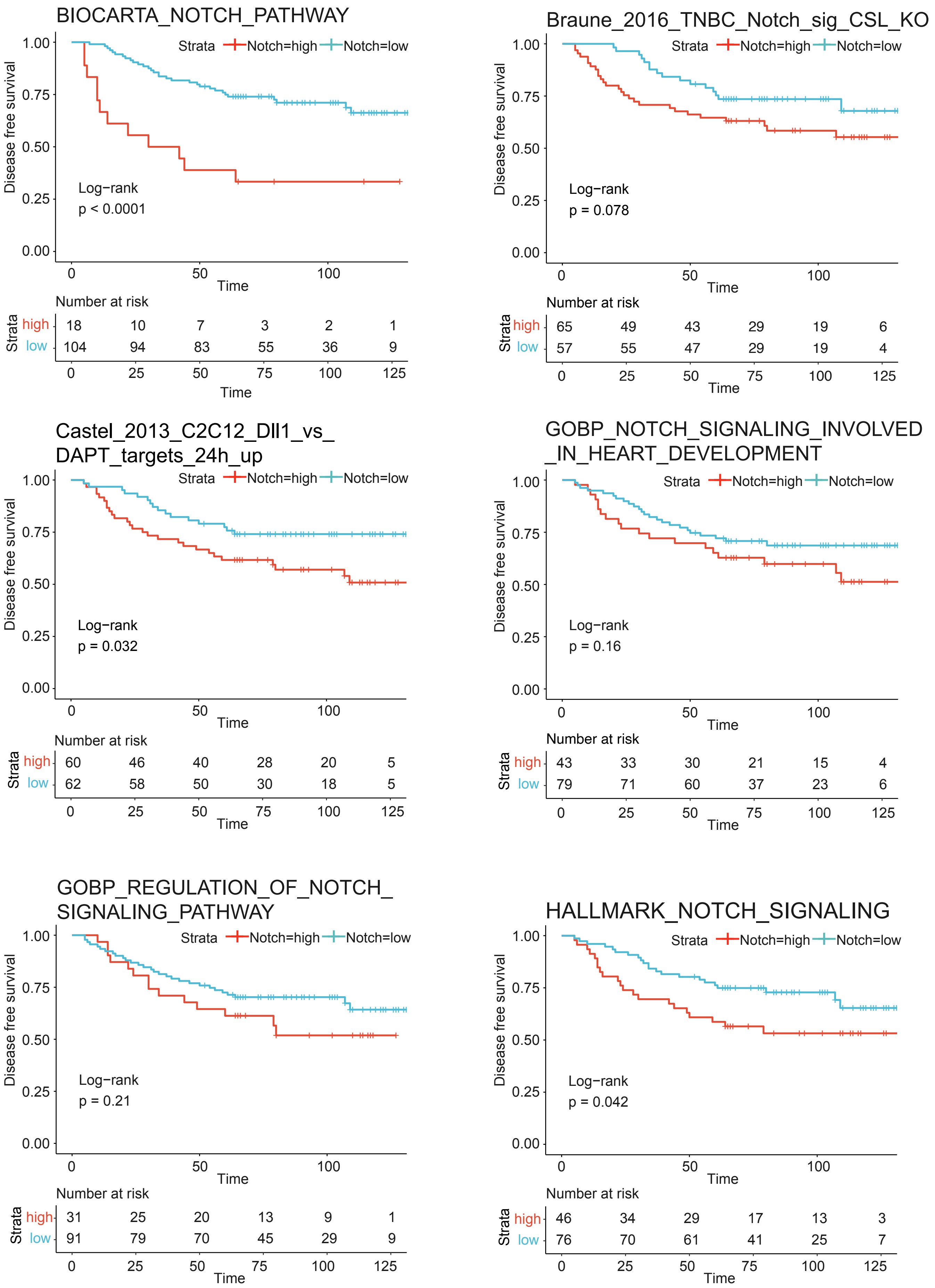

Fig. S10 continued

C

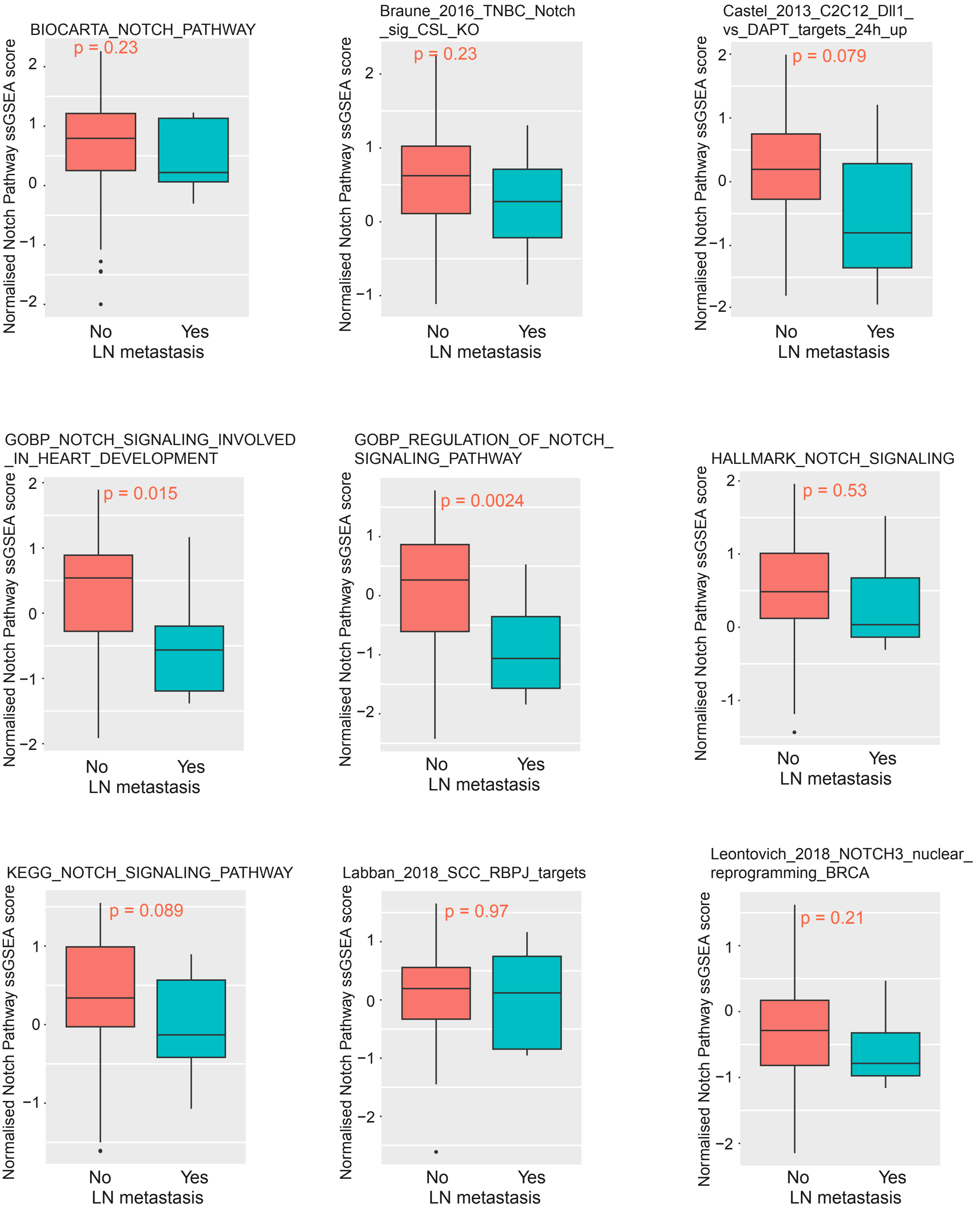

Fig. S10C continued

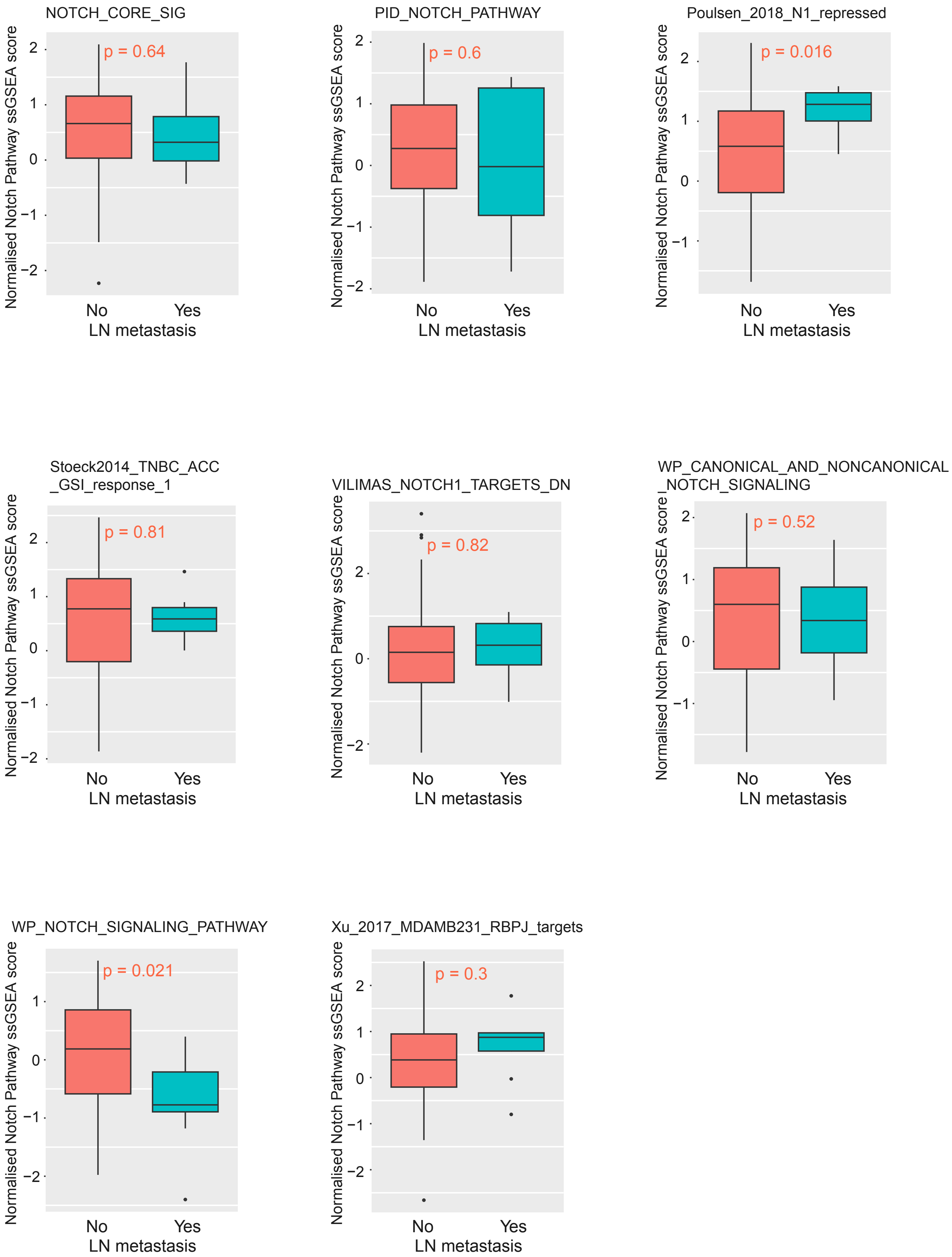

Fig. S10 continued

D

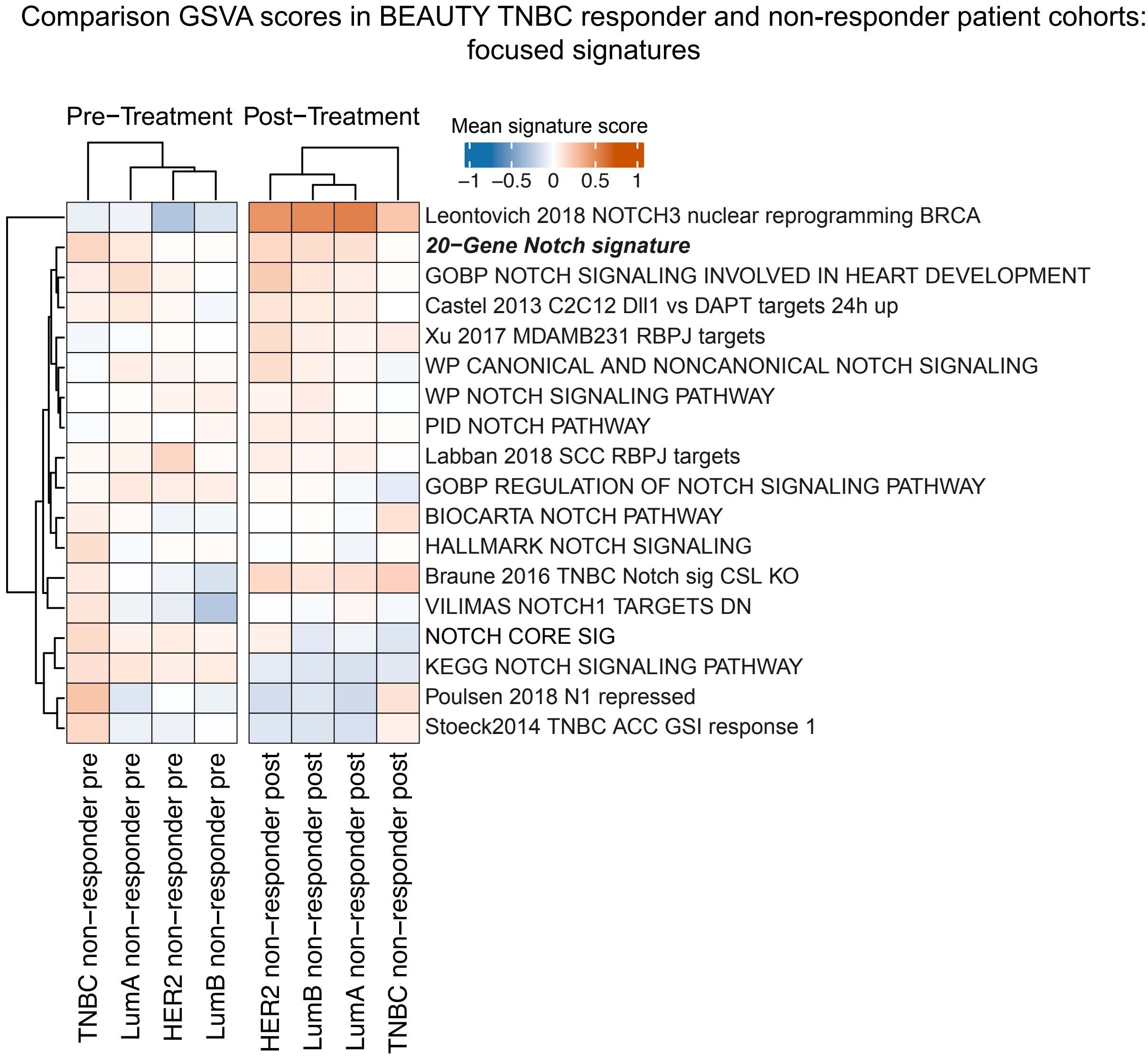


E

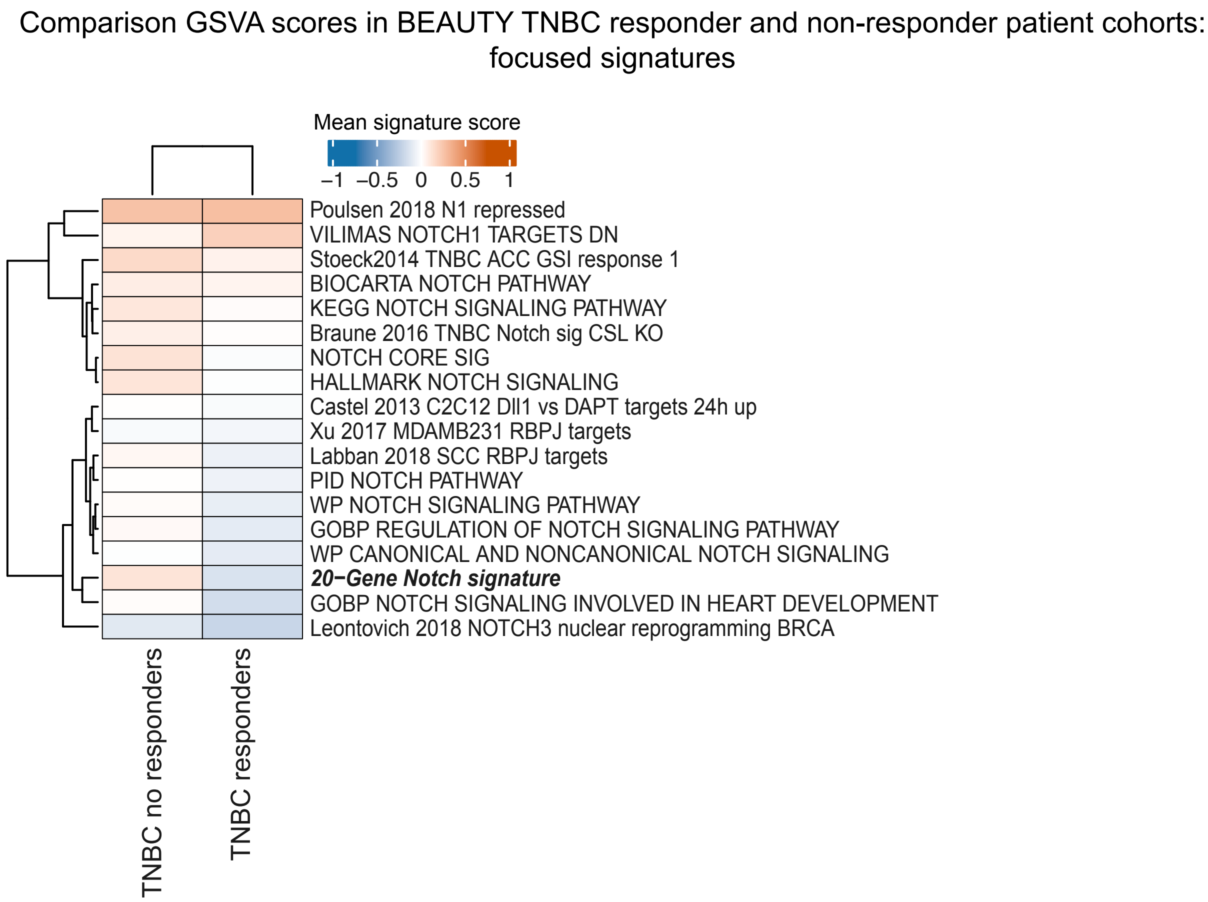

Supplement: Supplementary file 1 — Additional file 1. Fig. S1. A schematic pipeline of data processing for the 20-gene transcriptomic Notch signature. The code for the pipeline is provided at GitHub at the link: https://github.com/KarolinskaMerck/NotchBRCASignature. Figure S2. A. Expression of the four NOTCH receptors (NOTCH1-4), the androgen receptor (AR), ERBB2 (HER2), ERBB3 (HER3), ESR1 and pGR1 in the six basal-like cell lines. B. Expression of HES1 and NRARP in the six basal-like cell lines under “Notch on” and “Notch off” conditions. C. Principal component analysis (P1-20) of the transcriptomic data from the six cell lines under “Notch on”, “Notch off” or “ground state”, as indicated for 8-h (upper) and 72-h (lower) treatment. (*p < 0.05, **p < 0.01, ***p < 0.001 and ****p < 0.0001). Data for PC6 versus PC7 and PC7 versus PC10 PCA combinations are also shown. D. Euclidian distances between the Notch-on, ground-state and Notch-off conditions for each of the six cell lines and biological replicates. Figure S3. A. Analysis of left skewed P value distribution of the 14 candidate signatures from Fig. 1B. The coherence scores for each signature length in the TCGA-BRCA patient cohort for the candidate signatures are also presented. B. Selection of optimal signatures based on both the largest coherence score as well as smallest empirical P value with the smallest gene set size in the range between 10 and 30 genes. C. Euclidian distances between the Notch-on, ground-state and Notch-off conditions for each of the six cell lines. D. Comparison of the transcriptomes from the 19 cell lines in ground-state conditions to previously established transcriptomes from the same cell lines. E. Expression of at least one of the four Notch receptor genes in the 19 cell lines. F. The performance of the candidate signatures to classify Notch activation status in the 19-cell line dataset was evaluated by ROC curves and their AUC (+/− 95% Confidence Interval). G. ROC analysis of the 19 breast cancer cell line dataset for [file 13058_2023_1757_MOESM1_ESM.pdf]
